# Semisynthesis, characterisation, and antibacterial evaluation of a novel lecanoric acid-derived amide library

**DOI:** 10.3762/bjoc.22.81

**Published:** 2026-07-01

**Authors:** Ethan D Abbott, Sasha Hayes, Jonathan M White, Bernd H A Rehm, Rohan A Davis

**Affiliations:** 1 Institute for Biomedicine and Glycomics, Griffith University, Brisbane, QLD 4111, Australia; 2 School of Environment and Science, Griffith University, Brisbane, QLD 4111, Australia; 3 School of Chemistry and Bio21 Institute, The University of Melbourne, Melbourne, VIC 3010, Australia; 4 Centre for Cell Factories and Biopolymers, Grifﬁth University, Brisbane, QLD 4111, Australia; 5 NatureBank, Griffith University, Brisbane, QLD 4111, Australia

**Keywords:** amide, biodiscovery, depside, lecanoric acid, natural products, *Parmotrema tinctorum*, *Pseudomonas aeruginosa*, semisynthesis

## Abstract

The known lichen depside, lecanoric acid (**1**), was identified as a scaffold of interest for the generation of a unique semisynthetic biodiscovery screening library. Large-scale extraction and isolation on the Australian-sourced lichen *Parmotrema tinctorum* resulted in the purification of ≈1 g of the desired scaffold **1**, along with other known lichen metabolites that included divaricatic acid (**2**), orcinol (**3**), orsellinic acid (**4**), and methyl orsellinate (**5**). Parallel solution-phase synthesis using amidation chemistry on the abundant scaffold **1** afforded a series of novel amide derivatives **6**–**13** in high purity (>95%) and low to moderate yields (12–53%). All new semisynthetic compounds were fully characterised following 1D/2D NMR, MS and UV data analysis. Crystalline lecanoric acid was obtained during the chemical investigations of the lichen extract, enabling the first X-ray crystallographic analysis to be undertaken on this depside. Compounds **1**–**13** were evaluated for antibacterial activity against the human pathogen *Pseudomonas aeruginosa* using a biofilm inhibition assay. Of the new semisynthetics, amide analogue **12** showed the greatest planktonic cell growth inhibition (13% at 50 µM), whilst amide analogue **11** was the most active at inhibiting the formation of biofilm (21% at 50 µM).

## Introduction

Lichens are organisms resulting from the symbiotic relationship between a fungus (the mycobiont) and an alga (the photobiont) [1]. Through this partnership, the alga provides food for both itself and the fungal symbiont by reducing atmospheric CO_2_ into organic sugars. The fungal partner protects the organism from UV exposure, parasitic attacks, and animal predators by producing a diverse range of natural products [2–3]. Of the 17,000 lichen species that have been classified to date, 1,050 metabolites have been identified, half of which are unique to lichens [3–4]. Although only 10% of all lichen compounds have been subjected to bioactivity testing, several pharmacological properties have been documented [5]. Examples include the dibenzofuran derivative usnic acid that has been found to strongly inhibit cholinergic enzymes [e.g., AChE (IC_50_ 1.27 nM) and BChE (IC_50_ 0.24 nM)] that are drug targets for Alzheimer’s disease [6], and the depside atranorin, which has been shown to moderately inhibit the growth of the breast cancer cell line MDA-MB-231 (IC_50_ 5.36 µM) and the multidrug resistant W2mef strain of *Plasmodium falciparum* (IC_50_ 1.78 µM) [7–8]. Several endeavours have sought to enhance the bioactivity of known lichen metabolites through semisynthesis and medicinal chemistry. For example, chemical elaboration of the depside diffractaic acid yielded analogues with increased activity against a colorectal cancer stem cell [9]. Collectively, these promising results have inspired drug discovery researchers from around the world to pursue extraction, purification, characterisation, and bioactivity testing of lichen natural products and their semisynthetic derivatives.

**Figure 1:**
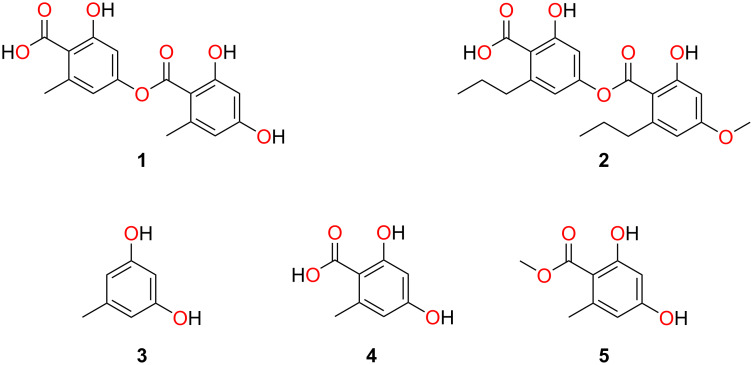
Chemical structures of the known lichen natural products lecanoric acid (**1**), divaricatic acid (**2**), orcinol (**3**), orsellinic acid (**4**), and methyl orsellinate (**5**), which were isolated from *Parmotrema tinctorum* CH_2_Cl_2_/MeOH extracts.

Owing to the Davis group’s ongoing interest in the semisynthesis of unique and rare biodiscovery screening libraries based on Australian natural products [10–14], a locally collected lichen, *Parmotrema tinctorum,* was selected for chemical investigations. Initial assessment (^1^H NMR spectroscopy and UHPLC–MS) of the crude CH_2_Cl_2_/MeOH extracts of *P. tinctorum* revealed the presence of a highly abundant natural product previously described as the depside, lecanoric acid (**1**, Figure 1). Several publications have outlined the antibiotic [3], anticancer [15], and antioxidant [16] properties of this compound, albeit with low to moderate bioactivity observed. Nevertheless, we considered this compound a desirable chemical scaffold for semisynthetic studies due to its high natural abundance in the lichen species and the compound’s phenolic, ester, and carboxylic acid moieties, which could all potentially serve as chemical handles for structural modifications via medicinal chemistry. Herein, we detail the large-scale extraction and purification of the targeted lichen natural product, lecanoric acid, along with the subsequent semisynthesis and characterisation of a novel biodiscovery screening library. Additionally, we report the antibacterial evaluation of the isolated natural products and semisynthetics against the Gram-negative human pathogen, *Pseudomonas aeruginosa*.

## Results and Discussion

Air-dried and ground *Parmotrema tinctorum* specimens were sequentially extracted with CH_2_Cl_2_ and MeOH, with the resulting extracts separately evaporated under reduced pressure. The CH_2_Cl_2_ extract was first fractionated using silica gel flash column chromatography, employing a stepwise solvent gradient from 100% *n*-hexane to 100% EtOAc. Each fraction was analysed by TLC, with fractions showing a similar profile combined to afford five fractions. These fractions were subsequently purified by semipreparative reversed-phase HPLC (RP-HPLC) using a linear gradient of MeOH/H_2_O/0.1% TFA. The resulting fractions were analysed by ^1^H NMR and UHPLC–MS, affording the known lichen metabolite divaricatic acid (**2**, Figure 1) [17]. The MeOH extract was also purified using RP-HPLC (MeOH/H_2_O/0.1% TFA) to afford the previously described lichen natural products lecanoric acid (**1**) [18–20], orcinol (**3**) [21], orsellinic acid (**4**) [22], and methyl orsellinate (**5**) [23].

**Figure 2:**
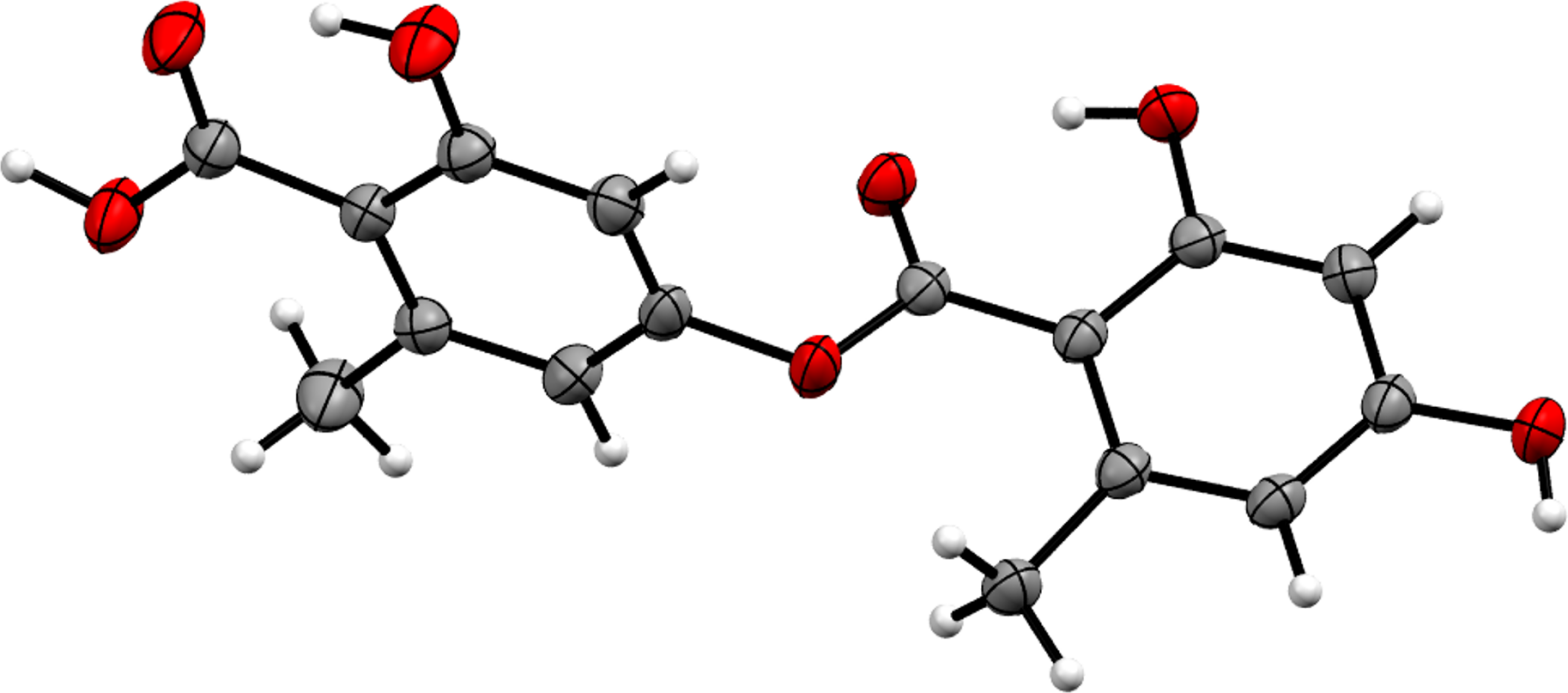
ORTEP drawing of lecanoric acid (**1**).

Spectroscopic and spectrometric literature values were compared with our data in order to identify the five known lichen compounds [17–23]. Crystalline lecanoric acid (**1**) was fortuitously obtained during our isolation studies, permitting the first X-ray crystallographic structure of this natural product to be obtained; the ORTEP drawing of **1** is shown in Figure 2.

The abundant depside scaffold **1** (970 mg) was subsequently utilised to generate a novel series of semisynthetic amides. This particular scaffold is known to undergo ester hydrolysis in basic environments (i.e., pH > 7), resulting in decomposition to orsellinic acid (**4**) [24]. Recognising the reactivity of the scaffold’s ester group, we sought to exploit this by reacting **1** with a series of commercially available primary amines to generate a series of chemically unique amides. Prior to the reaction of scaffold **1** with the eight commercially available primary amines, several trial amidation reactions were undertaken. This involved scaffold **1** (20 mg) being reacted with the abundant and moderately volatile isopentylamine (bp 95–97 °C) at three different reaction times (i.e., 1 h, 2 h, and 4 h), with temperature and stirring conditions kept consistent. Work-up and purification of each reaction involved the utilisation of semipreparative RP-HPLC (MeOH/H_2_O/0.1% TFA). ^1^H NMR and UHPLC–MS of all UV-active HPLC fractions from the three trial reactions confirmed that lecanoric acid had reacted as expected, with nucleophilic attack of the ester carbonyl by the primary amine (i.e., isopentylamine) taking place, thus forming the desired amide analogue **6** along with orsellinic acid (**4**).

**Scheme 1:**
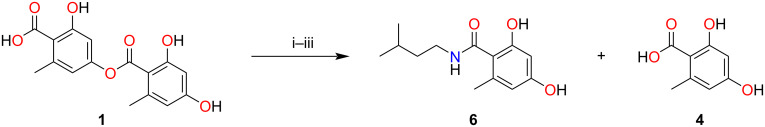
Reagents and conditions for the amidation optimisation study. i) Isopentylamine, rt, 1 h, yield 13%; ii) isopentylamine, rt, 2 h, yield 10%; iii) isopentylamine, rt, 4 h, yield 6%.

**Figure 3:**
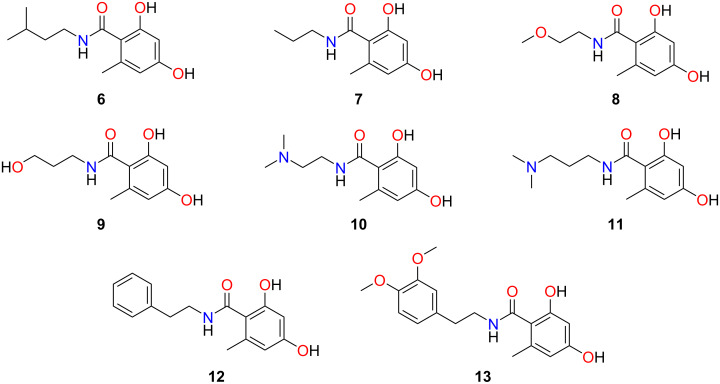
Chemical structures of the new semisynthetic amide analogues **6**–**13** generated from the purified lichen depside lecanoric acid (**1**).

The 1 h reaction was shown to produce the highest yield (i.e., 13%), generating 2.7 mg of the amide product **6** (Scheme 1) in high purity (>95%) following C_18_ RP-HPLC (MeOH/H_2_O/0.1% TFA). Large quantities of complex side-product mixtures were observed when the reaction time exceeded 1 h; orsellinic acid (**4**) was identified as one of the amidation degradation products. However, attempts to purify other reaction side-products from the unknown mixtures have proven unsuccessful to date. These inseparable mixtures were deemed to account for the low yield obtained for the amide derivative **6** from each of the three trial reactions. Despite the low yield of the targeted amide analogue **6**, the high purity and sufficient quantity for characterisation studies and biological testing encouraged us to complete the synthesis of the desired amide series. A further seven new amide analogues **7**–**13** were ultimately synthesised (Figure 3). These amidation reactions were all performed using 20 mg of **1**, with excess primary amine (500 µL) at rt and 1 h. Analogues were all purified using semipreparative C_18_ RP-HPLC (MeOH/H_2_O/0.1% TFA) with yields ranging from 12% to 53% and purities exceeding 95% (determined by 1D/2D NMR and UHPLC–MS analysis). All structures were fully confirmed and characterised using 1D/2D NMR, UV and IR spectroscopy, LRESIMS, and HRESIMS.

**Figure 4:**
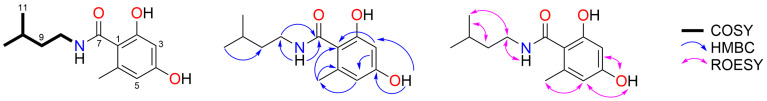
Key COSY, HMBC and ROESY correlations for *N*-isopentyl-2,4-dihydroxy-6-methylbenzamide (**6**).

An overview of the full characterisation of one amide analogue, 2,4-dihydroxy-*N*-isopentyl-6-methylbenzamide (**6**), is detailed below. Compound **6** was purified as a stable brown gum and was assigned the molecular formula C_13_H_19_NO_3_, following interpretation of the HRESIMS data (*m/z* 260.1259 [M + Na]^+^, calcd for C_13_H_19_NNaO_3_, 260.1257). The ^1^H NMR spectrum of **6** in DMSO-*d*_6_ (Table 1) revealed signals corresponding to three *C*-methyl groups (δ_H_ 2.11, 0.89, 0.87), two aromatic protons (δ_H_ 6.11 and 6.04), two methylene groups (δ_H_ 3.17, 1.35), one upfield methine proton (δ_H_ 1.64), and three exchangeable protons (δ_H_ 9.61, 9.35, 7.79). The exchangeable protons were confirmed through a D_2_O exchange ^1^H NMR experiment (see Supporting Information File 1, p. S26). The ^13^C NMR spectrum and HSQC spectra indicated a total of 13 carbons, including three *C*-methyl groups (δ_C_ 22.5, 22.5, 19.7), six aromatic carbons (δ_C_ 100.1–158.0), two methylene groups (δ_C_ 38.1, 37.1), one aliphatic methine carbon (δ_C_ 25.2), and one carbonyl carbon (δ_C_ 167.7). The presence of phenols in **6** was supported by a bathochromic shift of the UV spectrum upon addition of base (NaOH) [25–26]. One spin system was identified from the COSY spectrum, which was the isopentyl chain. The exchangeable proton δ_H_ 7.79 (1H, t, *J* = 5.6 Hz) indicated the existence of an amide moiety in **6**, which showed strong COSY correlations to one of the methylenes [δ_H_ 3.17 (2H, dt, *J* = 5.6, 6.6 Hz)] of the isopentyl chain. Furthermore, the substitution position of the isopentyl chain to the aromatic moiety was established through HMBC and ROESY correlations. Key COSY, HMBC, and ROESY correlations are shown below in Figure 4.

**Table 1:** NMR data of *N*-isopentyl-2,4-dihydroxy-6-methylbenzamide (**6**) in DMSO-*d*_6_.^a^

Position	δ_H_ (mult., *J* in Hz)	δ_C_, type	COSY	HMBC	ROESY

1		116.7, C			
2		156.1, C			
2-OH	9.61 (brs)				3
3	6.11 (d, 2.2)	100.1, CH	5,6-Me^w^	1,4,5,7^w^	2-OH,4-OH
4		158.0, C			
4-OH	9.35 (brs)			3,4,5	3,5
5	6.04 (d, 2.2)	108.1, CH	3,6-Me^w^	1,3,4,6-Me,7^w^	4-OH,6-Me
6		137.0, C			
6-Me	2.11 (s)	19.7, CH_3_	3^w^,5^w^	1^w^,2^w^,3^w^,4,5,6,7^w^	5,7-NH
7		167.7, C			
7-NH	7.79 (t, 5.6)		8	7,8,9	6-Me,8,9
8	3.17 (dt, 5.6, 6.6)	37.1, CH_2_	7-NH,9	7,8,9,10	7-NH,9,11,12
9	1.35 (dt, 6.6, 7.1)	38.1, CH_2_	8,10	8,10,11,12	8,11,12
10	1.64 (m)	25.2, CH	9,11,12	9,11,12	9,11,12
11	0.87 (d, 6.6)	22.5, CH_3_	10	9,10,12	8,9,10
12	0.89 (d, 6.6)	22.5, CH_3_	10	9,10,11	8,9,10

^a^Spectra recorded at 25 °C (500 MHz for ^1^H NMR and 125 MHz for ^13^C NMR). ^w^Weak.

Owing to the Davis group’s interest in discovering new natural products or derivatives with antibacterial properties, all compounds **1**–**13** were assessed via a biofilm inhibition assay that used the Gram-negative pathogen, *Pseudomonas aeruginosa* [27]. *P. aeruginosa* is an opportunistic and drug resistant nosocomial pathogen, which is responsible for an increasing number of fatal infections in critically-ill individuals [28]. The pathogen’s increasing resistance to most antibiotics has triggered drug discovery efforts to identify more effective compounds [29–30]. Hence, cell growth was monitored, and a resazurin metabolic assay was performed to determine the effect of both the natural products **1**–**5** and the new semisynthetic amide analogues **6**–**13** on biofilm formation. Overall, no significant activity was exhibited by any of the compounds at 50 µM. The natural products orcinol (**3**) and orsellinic acid (**4**), along with the new semisynthetic amide **11**, were shown to be the three most active compounds in the biofilm assay with inhibition values of 25%, 11%, and 21%, respectively (Table 2). The new semisynthetic amide analogue **12** was the most active compound at inhibiting the growth inhibition of *P. aeruginosa* with only 13% inhibition at 50 µM. Lecanoric acid (**1**) showed no biofilm or growth inhibition towards *P. aeruginosa* at 50 µM. Contrary to our data, Zhang et al. [31] reported that lecanoric acid weakly inhibited *P. aeruginosa* growth*.* These antibacterial data discrepancies could potentially be explained by differences in the assay methodologies (e.g., bacteria strain used, time points, etc), which are unfortunately not detailed by Zhang et al. [31].

**Table 2:** Inhibition of planktonic cell growth and biofilm inhibition of *Pseudomonas aeruginosa* for lichen natural products **1**–**5** and semisynthetic amide analogues **6**–**13**.

Compound	OD_600_ growth inhibition (%) at 50 µM ± SD	Biofilm inhibition (%) at 50 µM ± SD

**1**	−4.9 ± 2.5	−6.5 ± 0.5
**2**	2.4 ± 1.1	−6.6 ± 1.7
**3**	−0.2 ± 8.6	25.0 ± 27.9
**4**	0.5 ± 4.8	10.8 ± 8.1
**5**	−2.0 ± 2.5	−4.3 ± 4.2
**6**	−2.4 ± 1.1	−6.0 ± 5.7
**7**	−1.8 ± 7.1	−12.7 ± 0.5
**8**	−5.3 ± 3.1	−3.4 ± 0.6
**9**	6.4 ± 6.0	7.5 ± 5.7
**10**	3.1 ± 7.7	6.8 ± 10.0
**11**	−2.0 ± 1.7	20.6 ± 12.8
**12**	13.2 ± 3.2	−10.4 ± 8.2
**13**	2.8 ± 5.8	9.1 ± 23.8
tobramycin^a^	116.3 ± 0.6	96.8 ± 1.3
1% DMSO^a^	−11.2 ± 3.5	−5.2 ± 2.8

^a^Tobramycin and DMSO were used as positive and negative controls, respectively.

## Conclusion

Eight new semisynthetic amide analogues were generated from the previously reported and abundant depside, lecanoric acid (**1**), which was isolated from the lichen *Parmotrema tinctorum*. The new analogues were fully characterised using spectroscopic and spectrometric techniques. All natural products and semisynthetics were evaluated for their growth inhibition and antibiofilm potential against the nosocomial bacterium, *Pseudomonas aeruginosa*. A few compounds displayed weak inhibition in the bioassays, but most of the library exhibited no significant activity at 50 µM. The new amide analogues and lichen metabolites reported here have been added to the Davis Open Access Compound Library (DOACL), which currently consists of >1000 natural products, derivatives, and synthetics [32–34]. This unique collection of small molecules is screened regularly by Davis collaborators, therefore the compounds from this *Parmotrema tinctorum* project will be tested for novel bioactivities in the future.

## Experimental

### General experimental procedures

Melting points were measured using a Cole-Parmer melting point apparatus and are uncorrected. NMR spectra were recorded at 25 °C on a Bruker AVANCE III HD 500 MHz NMR spectrometer equipped with a cryoprobe. The ^1^H and ^13^C NMR chemical shifts were referenced to the solvent peak for DMSO-*d*_6_ at δ_H_ 2.50 and δ_C_ 39.52, CD_3_OD at δ_H_ 3.31 and δ_C_ 49.0, and CDCl_3_ at δ_H_ 7.26 and δ_C_ 77.00, respectively. LRESIMS data was recorded on an Thermo Fisher Scientific™ Dionex UltiMate™ 3000 RS UHPLC coupled to a Thermo Fisher Scientific™ ISQEC single quadruple ESI mass spectrometer using an analytical Thermo Scientific Accucore C_18_-bonded silica column (2.6 μm, 80 Å, 150 × 2.1 mm). HRESIMS data was acquired on a Bruker maXis II ETD ESI-qTOF. UV spectra were recorded using the Ocean Optics USB2000 Spectrometer coupled to a USB-ISS-UV–vis integrated sampling system. FTIR spectra were recorded on a Perkin Elmer Spectrum Two FTIR spectrophotometer equipped with Perkin Elmer’s UATR-TWO diamond ATR. Isolute^®^ silica SPE columns (5 g or 10 g; 50 µm, 60 Å) were utilised for flash-column chromatography. Alltech C_8_-bonded silica (30–40 µm, 60 Å) was used to pre-adsorb the extraction and reaction samples. The pre-adsorbed material was subsequently packed into an Alltech stainless steel guard cartridge (10 × 30 mm) then attached to a HPLC column prior to fractionation. A Thermo Fisher Scientific™ Electron Betasil phenyl-bonded silica column (5 μm, 100 Å, 150 × 21.2 mm) was used for RP-HPLC separations on the natural products. The column was fitted to a Thermo Fisher Scientific™ Dionex UltiMate™ 3000 UHPLC. A Thermo Fisher Scientific™ Electron Betasil C_18_-bonded silica column (5 μm, 100 Å, 150 × 21.2 mm) was used for RP-HPLC separations of the reaction products. The column was connected to a Waters 600 pump fitted with a Waters 996 photodiode array detector and a Gilson 717-plus autosampler. Merck silica gel 60 F_254_ pre-coated aluminium plates were used for thin-layer chromatography (TLC) and analysed under UV light at 254 nm. The raw lichen material was extracted with solvents at 200 rpm using an IKA™ KS 125 Basic^®^ orbital shaker operating at room temperature. Solvents were removed from lichen-derived crude extracts with a Büchi R-300 rotary evaporator. A GeneVac HT-4X centrifugal evaporator was used to remove solvents from HPLC fractions. Chemical reagents required for general experimentation and semisynthetic studies were purchased from Sigma-Aldrich. Honeywell Burdick & Jackson or Lab-Scan HPLC grade solvents were used for chromatography and MS. H_2_O was filtered using a Sartorius Stedium Arium^®^ Pro VF ultrapure system. All NMR spectra were processed using MestReNova version 14. Chemical structures were drawn using ChemDraw Ultra 12.0.2. All HPLC and UHPLC–MS results were analysed by Thermo Scientific™ Dionex™ Chromeleon™ 7.2.

### Lichen material

*Parmotrema tinctorum* was collected from a private property in Holland Park, Queensland, Australia on the 5th of April 2023. Patrick McCarthy and Jack Elix performed the taxonomic identification of the lichen. The freshly collected lichen was air-dried at room temperature for one month prior to the extraction and isolation chemistry. A voucher specimen has been deposited at the Institute for Biomedicine and Glycomics, Griffith University, Brisbane, QLD 4111, Australia.

### Extraction and isolation

The air-dried and ground specimen of *Parmotrema tinctorum* (10.0 g) was extracted sequentially with CH_2_Cl_2_ (250 mL, 2 h) and MeOH (250 mL, 2 h; 250 mL, 16 h). Each extract was filtered under gravity, with the CH_2_Cl_2_ and MeOH extracts dried under reduced pressure. The dried CH_2_Cl_2_ extract yielded a dark green amorphous powder (173.4 mg), whereas the MeOH extract produced a beige amorphous powder (2.1 g). The CH_2_Cl_2_ extract was pre-adsorbed onto silica (≈1 g) and then loaded onto an equilibrated Isolute^®^ SPE silica column (10 g, 50 µm, 60 Å) for flash chromatography. The column was subsequently flushed using a stepwise gradient solvent system of 10% EtOAc/90% *n*-hexane (100 mL), 15% EtOAc/85% *n*-hexane (100 mL), 20% EtOAc/80% *n*-hexane (100 mL), 30% EtOAc/70% *n*-hexane (100 mL), 40% EtOAc/60% *n*-hexane (100 mL), 50% EtOAc/50% *n*-hexane (100 mL), 60% EtOAc/40% *n*-hexane (100 mL), 75% EtOAc/25% *n*-hexane (100 mL), 100% EtOAc (100 mL), and 10% MeOH/90% CH_2_Cl_2_ (100 mL). All resulting fractions (81) were analysed by TLC using a MeOH/CH_2_Cl_2_ 10:90 solvent system, with fractions demonstrating a similar TLC profile combined to afford five fractions. Fractions 2 (36.2 mg), 3 (10.3 mg), and 4 (13.6 mg) were pre-adsorbed and subjected to semi-preparative RP-HPLC using a Betasil phenyl-bonded silica HPLC column. Isocratic conditions of 40% MeOH (0.1% TFA)/60% H_2_O (0.1% TFA) were initially run for 1 min, followed by a linear gradient to 100% MeOH (0.1% TFA) over 49 min, then isocratic conditions of 100% MeOH (0.1% TFA) were maintained for 10 min, all at a flow rate of 9 mL/min. Sixty fractions (60 × 1 min) were collected from time = 0 min. All resulting fractions were dried down, and UV-active fractions were analysed by ^1^H NMR and UHPLC–MS. This afforded the previously reported lichen metabolite, divaricatic acid (**2**, 15.2 mg, *t*_R_ 24–36 min, 0.15% dry wt).

The MeOH extract was pre-adsorbed to C_8_-bonded silica (≈1 g), packed into a stainless steel guard cartridge, and subjected to phenyl semi-preparative RP-HPLC separation. Isocratic conditions of 20% MeOH (0.1% TFA)/80% H_2_O (0.1% TFA) were initially run for 1 min, followed by a linear gradient to 90% MeOH (0.1% TFA)/10% H_2_O (0.1% TFA) for 119 min at a flow rate of 9 mL/min. One hundred and twenty fractions (120 × 1 min) were collected from time = 0 min. All resulting fractions were dried down and UV-active fractions were analysed by ^1^H NMR and UHPLC–MS. The known compounds lecanoric acid (**1**, 970.0 mg, *t*_R_ 63–100 min, 9.70% dry wt), orcinol (**3**, 13.2 mg, *t*_R_ 10–11 min, 0.13% dry wt), orsellinic acid (**4**, 40.2 mg, *t*_R_ 20–21 min, 0.40% dry wt), and methyl orsellinate (**5**, 63.0 mg, *t*_R_ 30–31 min, 0.63% dry wt) were isolated and subsequently identified following comparison of our data with literature values [17–23].

**Lecanoric acid (1):** beige needles [18–20]; mp 181–183 °C; UV (MeOH) λ_max_, nm (log ε): 221 (3.58), 270 (3.40), 306 (3.16); UV (MeOH + 1 drop of 1 M NaOH) λ_max_ , nm (log ε): 220 (3.76), 240 (3.30), 311 (3.65); IR (UATR) ν_max_: 3180, 2937, 1661, 1600, 1460, 1414, 1347, 1297, 1266, 1203, 1139, 1069, 901, 820, 692 cm^−1^; see Supporting Information File 1, pp S5–S12 for 1D/2D NMR data in DMSO-*d*_6_; LRMS–ESI(+) (*m/z*): 319 [M + H]^+^, 341 [M + Na]^+^; LRMS–ESI(−) (*m/z*): 317 [M − H]^−^.

**Divaricatic acid (2):** green gum [17]; see Supporting Information File 1, pp S13–S15 for 1D/2D NMR data in DMSO-*d*_6_; LRMS–ESI(−) (*m/z*): 387 [M −H]^−^.

**Orcinol (3):** brown gum [21]; see Supporting Information File 1, pp S16–S18 for 1D/2D NMR data in CD_3_OD; LRMS–ESI(+) (*m/z*): 125 [M + H]^+^.

**Orsellinic acid (4):** white amorphous powder [22]; see Supporting Information File 1, pp S19–S21 for 1D/2D NMR data in DMSO-*d*_6_; LRMS–ESI(+) (*m/z*): 169 [M + H]^+^.

**Methyl orsellinate (5):** brown gum [23]; see Supporting Information File 1, pp S22–S24 for 1D/2D NMR data in CDCl_3_; LRMS–ESI(+) (*m/z*): 183 [M + H]^+^.

### Synthesis of the amide library

***Trial amidation chemistry*.** Isopentylamine (72 equiv, 500 µL, 4.31 mmol) was added to the depside scaffold **1** (20 mg, 0.06 mmol) and the mixture was stirred at room temperature under three different time conditions: 1 h (reaction A), 2 h (reaction B), and 4 h (reaction C). The crude product from each reaction was pre-adsorbed to C_8_-bonded silica (≈1 g) and subsequently subjected to RP-HPLC using a semi-preparative C_18_-bonded silica Betasil column. Isocratic conditions of 10% MeOH (0.1% TFA)/90% H_2_O (0.1% TFA) were initially run for 10 min, followed by a linear gradient to 100% MeOH (0.1% TFA) over 40 min. Isocratic conditions of 100% MeOH (0.1% TFA) were maintained in the final 10 min of the run. Sixty fractions (60 × 1 min) were collected from time = 0 min. All resulting fractions were dried down, and UV-active fractions were analysed by ^1^H NMR and UHPLC–MS, with compound **6** eluting at 34–35 min. Yields for the intended analogue **6** across the three reactions were 13% (reaction A, 1 h), 10% (reaction B, 2 h), and 6% (reaction C, 4 h), respectively.

Due to the better yield of reaction A all other semisynthetic studies employed these optimised conditions. Thus, seven other commercially available primary amine reagents (500 µL) were reacted with the depside scaffold **1** (20 mg, 0.06 mmol) at room temperature for 1 h to form the new amide series **6**–**13**. All amides were purified using the C_18_ semi-preparative RP-HPLC conditions employed during the time-based optimisation studies. However, compounds **9** and **13** required further purification. Semi-pure compound **9** was subjected to C_18_ semi-preparative RP-HPLC using a Betasil C_18_-bonded silica HPLC column. Isocratic conditions of 10% MeOH (0.1% TFA)/90% H_2_O (0.1% TFA) were initially run for 10 min, followed by a linear gradient to 50% MeOH (0.1% TFA)/50% H_2_O (0.1% TFA) over 40 min, and lastly isocratic conditions of 50% MeOH (0.1% TFA)/50% H_2_O (0.1% TFA) were employed for 10 min, all at a flow rate of 9 mL/min. Sixty fractions (60 × 1 min) were collected from time = 0 min. All resulting fractions were dried down, and UV-active fractions were analysed by ^1^H NMR and UHPLC–MS, with compound **9** eluting at 9–10 min. Semi-pure compound **13** was also subjected to another round of purification using semi-preparative HPLC and a Betasil C_18_-bonded silica RP-HPLC column. Isocratic conditions of 10% MeOH (0.1% TFA)/90% H_2_O (0.1% TFA) were initially run for 10 min, followed by a linear gradient to 70% MeOH (0.1% TFA)/30% H_2_O (0.1% TFA) over 40 min, and lastly isocratic conditions of 70% MeOH (0.1% TFA)/30% H_2_O (0.1% TFA) for 10 min, all at a flow rate of 9 mL/min. Sixty fractions (60 × 1 min) were collected from time = 0 min. All resulting fractions were dried down, and UV-active fractions were analysed by ^1^H NMR and UHPLC–MS, with compound **13** eluting at 37–38 min.

***N*-Isopentyl-2,4-dihydroxy-6-methylbenzamide (6):** brown gum (2.7 mg, yield 13%); UV (MeOH) λ_max_, nm (log ε): 220 (3.38), 246 (2.99), 282 (2.76); UV (MeOH + 1 drop of 1 M NaOH) λ_max_, nm (log ε): 230 (3.60), 278 (3.34), 310 (3.24); IR (UATR) ν_max_: 3292, 2960, 1606, 1544, 1270, 1170, 1024, 845, 724 cm^−1^; see Supporting Information File 1, pp S25–S32 for 1D/2D NMR data in DMSO-*d*_6_; LRMS–ESI(+) (*m/z*): 238 [M + H]^+^, 260 [M + Na]^+^; HRMS–ESI(+) (*m/z*): [M + Na]^+^ calcd for C_13_H_19_NNaO_3_, 260.1257; found, 260.1259.

***N*-Propyl-2,4-dihydroxy-6-methylbenzamide (7):** brown gum (5.7 mg, yield 28%); UV (MeOH) λ_max_, nm (log ε): 220 (3.35), 249 (2.96), 282 (2.75); UV (MeOH + 1 drop of 1 M NaOH) λ_max_, nm (log ε): 230 (3.52), 279 (3.29), 312 (3.16); IR (UATR) ν_max_: 3288, 2967, 1606, 1544, 1463, 1271, 1171, 842 cm^−1^; see Supporting Information File 1, pp S34–S40 for 1D/2D NMR data in DMSO-*d*_6_; LRMS–ESI(+) (*m/z*) 210 [M + H]^+^, 232 [M + Na]^+^; HRMS–ESI(+) (*m/z*): [M + Na]^+^ calcd for C_11_H_15_NNaO_3_, 232.0944; found, 232.0946.

***N*-(2-Methoxyethyl)-2,4-dihydroxy-6-methylbenzamide (8):** brown gum (6.7 mg, yield 33%); UV (MeOH) λ_max_, nm (log ε): 222 (3.89), 250 (3.48), 282 (3.28); UV (MeOH + 1 drop of 1 M NaOH) λ_max_, nm (log ε): 231 (4.13), 280 (3.87), 314 (3.71); IR (UATR) ν_max_: 3287, 2941, 1686, 1608, 1544, 1462, 1336, 1273, 1171, 1017, 841, 707, 641, 524 cm^−1^; see Supporting Information File 1, pp S42–S48 for 1D/2D NMR data in DMSO-*d*_6_; LRMS–ESI(+) (*m/z*): 226 [M + H]^+^, 248 [M + Na]^+^; HRMS–ESI(+) (*m/z*): [M + Na]^+^ calcd for C_11_H_15_NNaO_4_, 248.0893; found, 248.0895.

***N*-(3-Hydroxypropyl)-2,4-dihydroxy-6-methylbenzamide (9):** brown gum (2.4 mg, yield 12%); UV (MeOH) λ_max_, nm (log ε): 220 (3.42), 250 (3.05), 282 (2.86); UV (MeOH + 1 drop of 1 M NaOH) λ_max_, nm (log ε): 231 (3.61), 279 (3.38), 311 (3.27); IR (UATR) ν_max_: 3342, 2981, 1606, 1547, 1331, 1170, 1059, 841 cm^−1^; see Supporting Information File 1, pp S50–S56 for 1D/2D NMR data in DMSO-*d*_6_; LRMS–ESI(+) (*m/z*): 226 [M + H]^+^, 248 [M + Na]^+^; HRMS–ESI(+) (*m/z*): [M + Na]^+^ calcd for C_11_H_15_NNaO_4_, 248.0893; found, 248.0893.

**TFA salt of *N*-(2-(dimethylamino)ethyl)-2,4-dihydroxy-6-methylbenzamide (10):** brown gum (7.1 mg, yield 35%); UV (MeOH) λ_max_, nm (log ε): 220 (3.27), 250 (2.87), 282 (2.63); UV (MeOH + 1 drop of 1 M NaOH) λ_max_, (log ε): 223 (3.46), 279 (3.17), 312 (3.05) nm; IR (UATR) ν_max_: 3252, 1681, 1608, 1468, 1338, 1204, 1177, 1135, 1021, 840, 801, 723 cm^−1^; see Supporting Information File 1, pp S58–S64 for 1D/2D NMR data in DMSO-*d*_6_; LRMS–ESI(+) (*m/z*): 239 [M + H]^+^, 261 [M + Na]^+^; HRMS–ESI(+) (*m/z*): [M + H]^+^ calcd for C_12_H_19_N_2_O_3_, 239.1390; found, 239.1391.

**TFA salt of *N*-(3-(dimethylamino)propyl)-2,4-dihydroxy-6-methylbenzamide (11):** brown gum (5.5 mg, yield 27%); UV (MeOH) λ_max_, nm (log ε): 220 (3.44), 249 (3.04), 282 (2.84); UV (MeOH + 1 drop of 1 M NaOH) λ_max_, nm (log ε): 229 (3.61), 266 (3.27), 301 (3.22) nm; IR (UATR) ν_max_: 3250, 2996, 1681, 1608, 1545, 1331, 1174, 1134, 840, 801, 723 cm^−1^; see Supporting Information File 1, pp S66–S72 for 1D/2D NMR data in DMSO-*d*_6_; LRMS–ESI(+) (*m/z*): 253 [M + H]^+^, 275 [M + Na]^+^; HRMS–ESI(+) (*m/z*): [M + H]^+^ calcd for C_13_H_21_N_2_O_3_, 253.1547; found, 253.1546.

**2,4-Dihydroxy-6-methyl-*N*-phenethylbenzamide (12):** brown gum (10.7 mg, yield 53%); UV (MeOH) λ_max_, nm (log ε): 220 (3.06), 250 (2.64), 282 (2.38); UV (MeOH + 1 drop of 1 M NaOH) λ_max_, nm (log ε): 225 (3.24), 279 (2.88), 308 (2.81); IR (UATR) ν_max_: 3269, 1604, 1536, 1497, 1455, 1337, 1268, 1170, 1136, 1004, 843, 750, 700 cm^−1^; see Supporting Information File 1, pp S74–S81 for 1D/2D NMR data in DMSO-*d*_6_; LRMS–ESI(+) (*m/z*): 272 [M + H]^+^, 294 [M + Na]^+^; HRMS–ESI(+) (*m/z*): [M + Na]^+^ calcd for C_16_H_17_NNaO_3_, 294.1101; found, 294.1098.

***N*-(3,4-Dimethoxyphenethyl)-2,4-dihydroxy-6-methylbenzamide (13):** brown gum (2.5 mg, yield 12%); UV (MeOH) λ_max_, nm (log ε): 220 (3.57), 279 (3.11); UV (MeOH + 1 drop of 1 M NaOH) λ_max_, nm (log ε): 230 (3.69), 279 (3.41), 313 (3.18); IR (UATR) ν_max_: 2981, 1708, 1594, 1516, 1331, 1265, 1158, 1024, 807 cm^−1^; see Supporting Information File 1, pp S83–S89 for 1D/2D NMR data in DMSO-*d*_6_; LRMS–ESI(+) (*m/z*): 332 [M + H]^+^, 354 [M + Na]^+^; HRMS–ESI(+) (*m/z*): [M + Na]^+^ calcd for C_18_H_21_NNaO_5_, 354.1312; found, 354.1308.

### X-ray crystallography analysis of lecanoric acid (**1**)

Intensity data were collected on a Rigaku XtalLAB Synergy diffractometer using Cu Kα radiation at 200.0(1) K using an Oxford Cryostream cooling device. The structures were solved by direct methods and difference Fourier synthesis [35]. Hydrogen atoms bound to the carbon atom were placed at their idealized positions and included in subsequent refinement cycles. The hydrogen atoms attached to heteroatoms were located from difference Fourier maps and refined freely with isotropic displacement parameters. A thermal ellipsoid plot was generated using the program Mercury integrated within the WINGX suite of programs [36–37]. Crystallographic data for **1** has been deposited with the Cambridge Crystallographic Data Centre and assigned CCDC deposit code 2541353. This data can be obtained free of charge from the Cambridge Crystallographic Data Centre via https://www.ccdc.cam.ac.uk/data_request/cif.

Crystal data for lecanoric acid (**1**): C_16_ H_14_ O_7_.(C_2_H_6_OS), *M* = 396.40, *T* = 200.0 K, λ = 1.54184 Å, monoclinic, space group *P*2_1_/*c*, *a =* 17.0659(2) Å, *b =* 7.45980(10) Å*, c =* 16.1534(2) Å, β = 117.493(2)°, *V =* 1824.22(5) Å*^3^*, *Z* = 4, *D*_c_ = 1.443 mg M^−3^, µ (Cu Kα) 1.981 mm^−1^, F(000) = 832, crystal size 0.33 × 0.27 × 0.06 mm^3^; 27980 reflections measured, θ_max_ = 776.98°, 3842 independent reflections (*R*_int_ = 0.0624); final *R* = 0.0348 [*I* > 2σ(*I*), 3459 data] and *wR*(*F*^2^) = 0.0971 (all data); GOF 1.067.

### Bacterial strains, chemicals, and media

Wild-type *Pseudomonas aeruginosa* strain PAO1 (prototrophic wild-type) was grown in Luria–Bertani (LB) medium (10 g/L tryptone, 10 g/L sodium chloride, and 5 g/L yeast extract) at 37 °C. Tobramycin and resazurin were obtained from Sigma-Aldrich.

### Biofilm inhibition assay

In a similar manner to Tran et al. and Hayes et al., the overnight cultures at 37 °C in LB medium were washed once with sterile saline 0.9% (w/v) and adjusted to an OD_600_ of 0.05, and 1% inoculum was transferred into fresh LB medium [38–39]. Following the incubation at 37 °C, at 200 rpm for 6–6.5 h to reach the mid-log phase, the cells were washed once with sterile saline 0.9% and diluted to an OD_600_ of 0.01. 135 µL aliquots were dispensed into 96-well plates, test compounds (15 µL) were loaded prior to the addition of bacteria. The plates were incubated for 24 h at 37 °C in static conditions. The effects of the compounds at 50 µM on bacterial growth and viability of biofilm bacteria were determined by the OD_600_ and resazurin metabolic assay, respectively. The final concentrations of DMSO in the assays was 1%. The negative control or untreated cultures consisted of inoculum and 1% DMSO. Antibiotic tobramycin (16 µg/mL) was used as a positive control. The initial OD_600_ and final OD_600_ were read before incubation at 37 °C and after 24 h incubation, respectively, followed by assessment of biofilm viability by resazurin metabolic assay. The experiments were carried out with three technical replicates.

Resazurin sodium salt was dissolved in Milli-Q water at 0.2% (w/v) and filter-sterilized. The solution was stored at −20 °C in the dark. The assay was performed as previously described. The cultures were withdrawn, and the plates were washed twice with sterile water. To remove the remaining water in the wells, the plates were tapped with autoclaved paper towels. 50 µL of the 0.02% diluted resazurin solution in LB medium was added into each well followed by incubation at 37 °C for 5–6 h. A microplate reader was used to measure the fluorescence intensity (excitation 530 nm, emission 590 nm). Data collected was determined as a function of fluorescence percentage. The biology data format for this paper is similar to previous Davis publications [40].

## Supporting Information

File 1 NMR data tables and 1D/2D NMR spectra for natural products **1**–**5** and semisynthetic amide analogues **6**–**13**, additionally, HRESIMS data for all semisynthetics.

## Data Availability

Data generated and analyzed during this study is available from the corresponding author upon reasonable request.
