# Supplementary material for: Semisynthesis, characterisation, and antibacterial evaluation of a novel lecanoric acid-derived amide library
Source: Beilstein J Org Chem. 2026 Jul 1;22:1023–32. doi: 10.3762/bjoc.22.81 (PMC13338597; doi:10.3762/bjoc.22.81)
Supplement: File 1 — NMR data tables and 1D/2D NMR spectra for natural products 1–5 and semisynthetic amide analogues 6–13, additionally, HRESIMS data for all semisynthetics. [file Beilstein_J_Org_Chem-22-1023-s001.pdf]

BEILSTEIN JOURNAL OF ORGANIC CHEMISTRY

Supporting Information

for

Semisynthesis, characterisation, and antibacterial evaluation of a novel lecanoric acid-derived amide library

Ethan D. Abbott, Sasha Hayes, Jonathan M. White, Bernd H. A. Rehm
and Rohan A. Davis

Beilstein J. Org. Chem. **2026,** *22,* 1023–1032. doi:10.3762/bjoc.22.81

NMR data tables and 1D/2D NMR spectra for natural products 1–5 and semisynthetic amide analogues 6–13, additionally, HRESIMS data for all semisynthetics

License and Terms: This is a supporting information file under the terms of the Creative Commons Attribution License (https://creativecommons.org/licenses/by/4.0). Please note that the reuse, redistribution and reproduction in particular requires that the author(s) and source are credited and that individual graphics may be subject to special legal provisions.
The license is subject to the *Beilstein Journal of Organic Chemistry* terms and conditions: (https://www.beilstein-journals.org/bjoc/terms)

Table of contents

^{1}H NMR spectrum of lecanoric acid (**1**) in DMSO-d_6 .... S5
^{1}H NMR spectrum of lecanoric acid (**1**) in DMSO-d_6 and D_2O S6
^{13}C NMR spectrum of lecanoric acid (**1**) in DMSO-d_6 .... S7
HSQC spectrum of lecanoric acid (**1**) in DMSO-d_6 .... S8
HMBC spectrum of lecanoric acid (**1**) in DMSO-d_6 .... S9
COSY spectrum of lecanoric acid (**1**) in DMSO-d_6 .... S10
ROESY spectrum of lecanoric acid (**1**) in DMSO-d_6 .... S11
NMR data table for lecanoric acid (**1**) in DMSO-d_6 .... S12
^{1}H NMR spectrum of divaricatic acid (**2**) in DMSO-d_6 .... S13
^{13}C NMR spectrum of divaricatic acid (**2**) in DMSO-d_6 .... S14
NMR data table for divaricatic acid (**2**) in DMSO-d_6 .... S15
^{1}H NMR spectrum of orcinol (**3**) in CD_3OD S16
^{13}C NMR spectrum of orcinol (**3**) in CD_3OD S17
NMR data table for orcinol (**3**) in CD_3OD S18
^{1}H NMR spectrum of orsellinic acid (**4**) in DMSO-d_6 .... S19
^{13}C NMR spectrum of orsellinic acid (**4**) in DMSO-d_6 .... S20
NMR data table for orsellinic acid (**4**) in DMSO-d_6 .... S21
^{1}H NMR spectrum of methyl orsellinate (**5**) in $CDCl_3$ S22
^{13}C NMR spectrum of methyl orsellinate (**5**) in $CDCl_3$ S23
NMR data table for methyl orsellinate (**5**) in $CDCl_3$ S24

^{1}H NMR spectrum of compound **6** in DMSO-d_6 .... S25

^{1}H NMR spectrum of compound **6** in DMSO-d_6 and D_2O S26

^{13}C NMR spectrum of compound **6** in DMSO-d_6 .... S27

HSQC spectrum of compound **6** in DMSO-d_6 .... S28

HMBC spectrum of compound **6** in DMSO-d_6 .... S29

COSY spectrum of compound **6** in DMSO-d_6 .... S30

ROESY spectrum of compound **6** in DMSO-d_6 .... S31

NMR data table for compound **6** in DMSO-d_6 .... S32

High resolution mass spectrum of compound **6** S33

^{1}H NMR spectrum of compound **7** in DMSO-d_6 .... S34

^{13}C NMR spectrum of compound **7** in DMSO-d_6 .... S35

HSQC spectrum of compound **7** in DMSO-d_6 .... S36

HMBC spectrum of compound **7** in DMSO-d_6 .... S37

COSY spectrum of compound **7** in DMSO-d_6 .... S38

ROESY spectrum of compound **7** in DMSO-d_6 .... S39

NMR data table for compound **7** in DMSO-d_6 .... S40

High resolution mass spectrum of compound **7** S41

^{1}H NMR spectrum of compound **8** in DMSO-d_6 .... S42

^{13}C NMR spectrum of compound **8** in DMSO-d_6 .... S43

HSQC spectrum of compound **8** in DMSO-d_6 .... S44

HMBC spectrum of compound **8** in DMSO-d_6 .... S45

COSY spectrum of compound **8** in DMSO-d_6 .... S46

ROESY spectrum of compound **8** in DMSO-d_6 .... S47

NMR data table for compound **8** in DMSO-d_6 .... S48
High resolution mass spectrum of compound **8** S49
^{1}H NMR spectrum of compound **9** in DMSO-d_6 .... S50
^{13}C NMR spectrum of compound **9** in DMSO-d_6 .... S51
HSQC spectrum of compound **9** in DMSO-d_6 .... S52
HMBC spectrum of compound **9** in DMSO-d_6 .... S53
COSY spectrum of compound **9** in DMSO-d_6 .... S54
ROESY spectrum of compound **9** in DMSO-d_6 .... S55
NMR data table for compound **9** in DMSO-d_6 .... S56
High resolution mass spectrum of compound **9** S57
^{1}H NMR spectrum of compound **10** in DMSO-d_6 .... S58
^{13}C NMR spectrum of compound **10** in DMSO-d_6 .... S59
HSQC spectrum of compound **10** in DMSO-d_6 .... S60
HMBC spectrum of compound **10** in DMSO-d_6 .... S61
COSY spectrum of compound **10** in DMSO-d_6 .... S62
ROESY spectrum of compound **10** in DMSO-d_6 .... S63
NMR data table for compound **10** in DMSO-d_6 .... S64
High resolution mass spectrum of compound **10** S65
^{1}H NMR spectrum of compound **11** in DMSO-d_6 .... S66
^{13}C NMR spectrum of compound **11** in DMSO-d_6 .... S67
HSQC spectrum of compound **11** in DMSO-d_6 .... S68
HMBC spectrum of compound **11** in DMSO-d_6 .... S69
COSY spectrum of compound **11** in DMSO-d_6 .... S70

ROESY spectrum of compound **11** in DMSO-d_6 .... S71
NMR data table for compound **11** in DMSO-d_6 .... S72
High resolution mass spectrum of compound **11** S73
^{1}H NMR spectrum of compound **12** in DMSO-d_6 .... S74
^{1}H NMR spectrum of compound **12** in DMSO-d_6 and D_2O S75
^{13}C NMR spectrum of compound **12** in DMSO-d_6 .... S76
HSQC spectrum of compound **12** in DMSO-d_6 .... S77
HMBC spectrum of compound **12** in DMSO-d_6 .... S78
COSY spectrum of compound **12** in DMSO-d_6 .... S79
ROESY spectrum of compound **12** in DMSO-d_6 .... S80
NMR data table for compound **12** in DMSO-d_6 .... S81
High resolution mass spectrum of compound **12** S82
^{1}H NMR spectrum of compound **13** in DMSO-d_6 .... S83
^{13}C NMR spectrum of compound **13** in DMSO-d_6 .... S84
HSQC spectrum of compound **13** in DMSO-d_6 .... S85
HMBC spectrum of compound **13** in DMSO-d_6 .... S86
COSY spectrum of compound **13** in DMSO-d_6 .... S87
ROESY spectrum of compound **13** in DMSO-d_6 .... S88
NMR data table for compound **13** in DMSO-d_6 .... S89
High resolution mass spectrum of compound **13** S90

^{1}H NMR spectrum of lecanoric acid (**1**) in DMSO-d_6

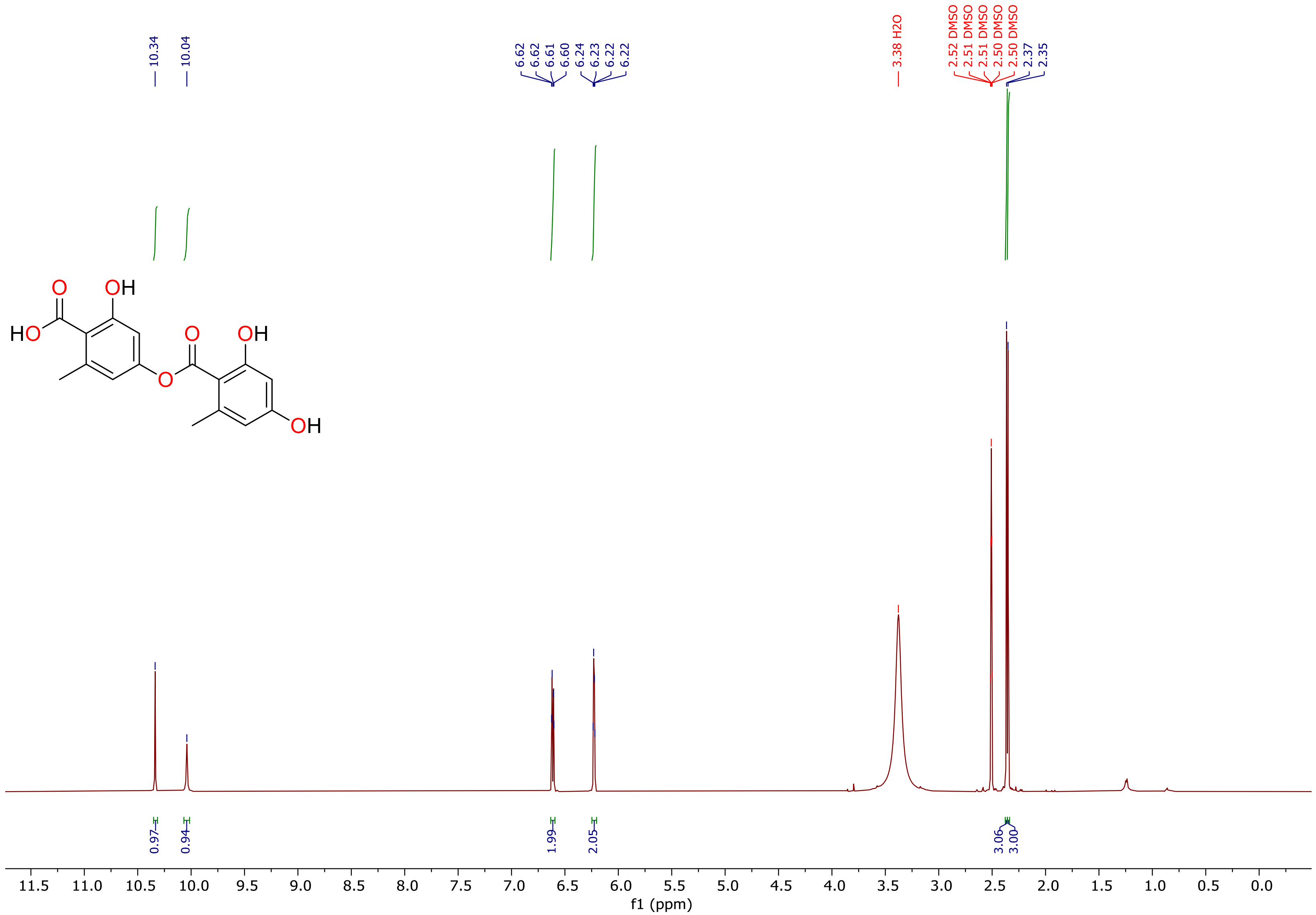

^{1}H NMR spectrum of lecanoric acid (**1**) in DMSO-d_6 and D_2O

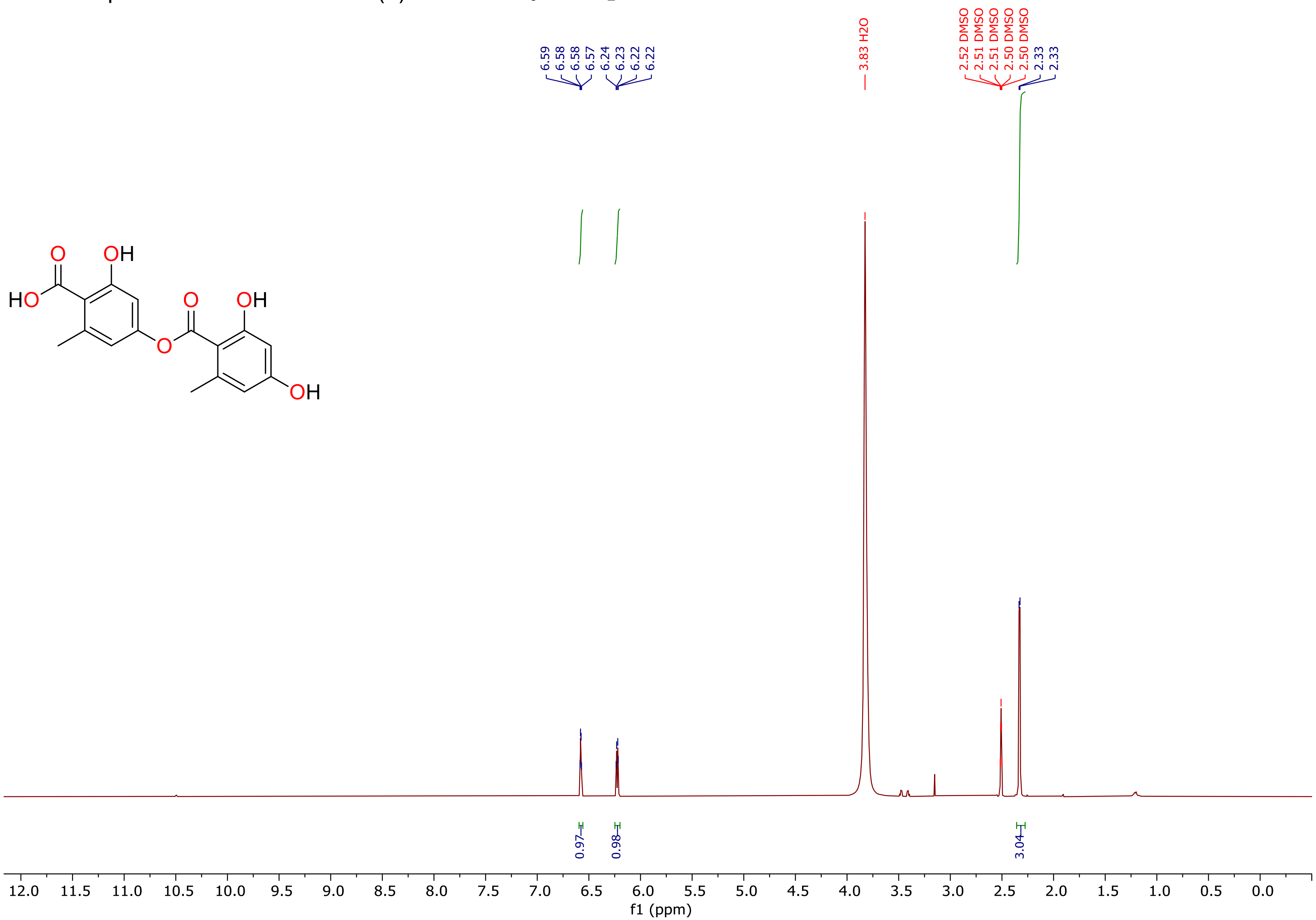

^{13}C NMR spectrum of lecanoric acid (**1**) in DMSO-d_6

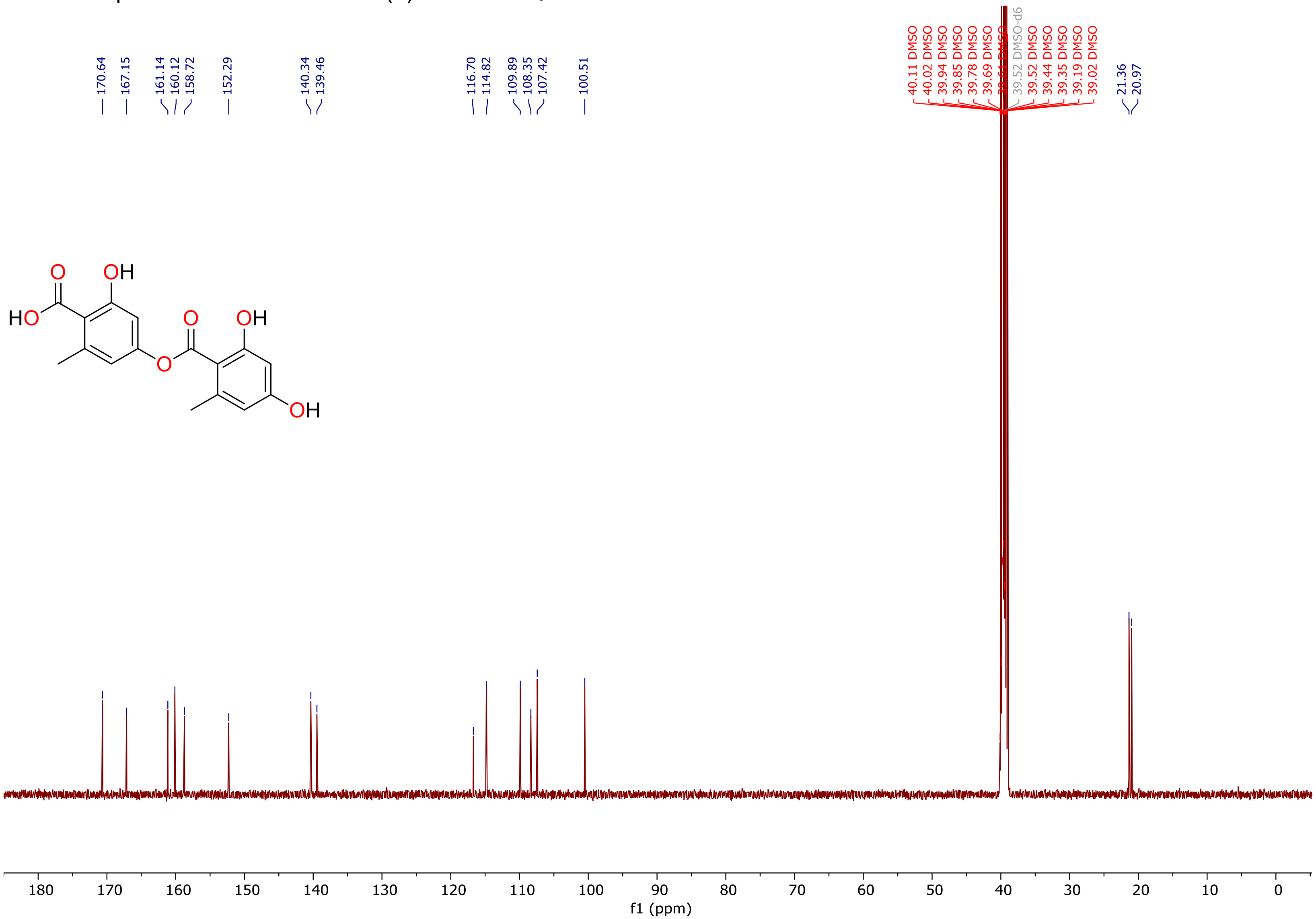

HSQC spectrum of lecanoric acid (**1**) in DMSO-d_6

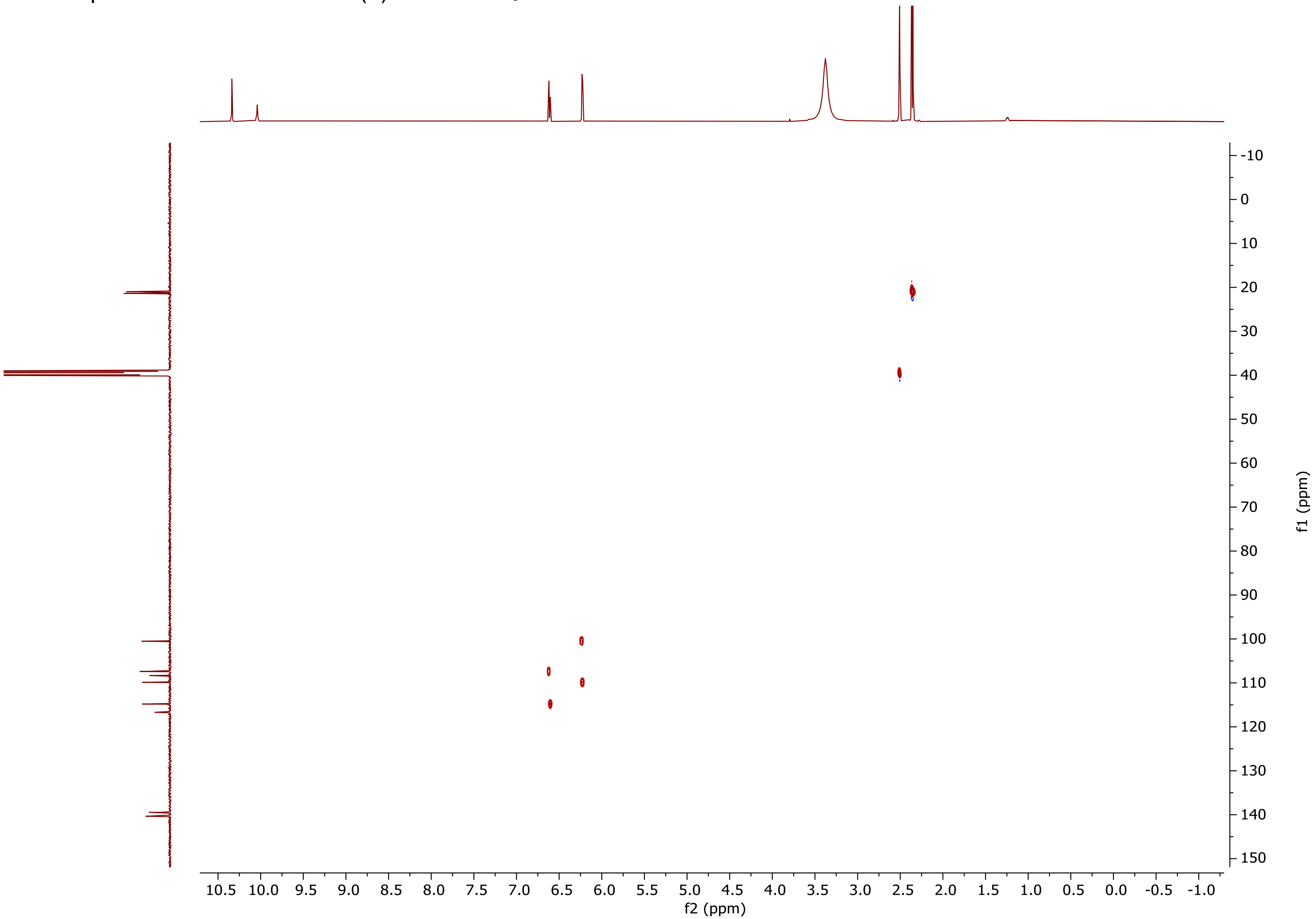

HMBC spectrum of lecanoric acid (**1**) in DMSO-d_6

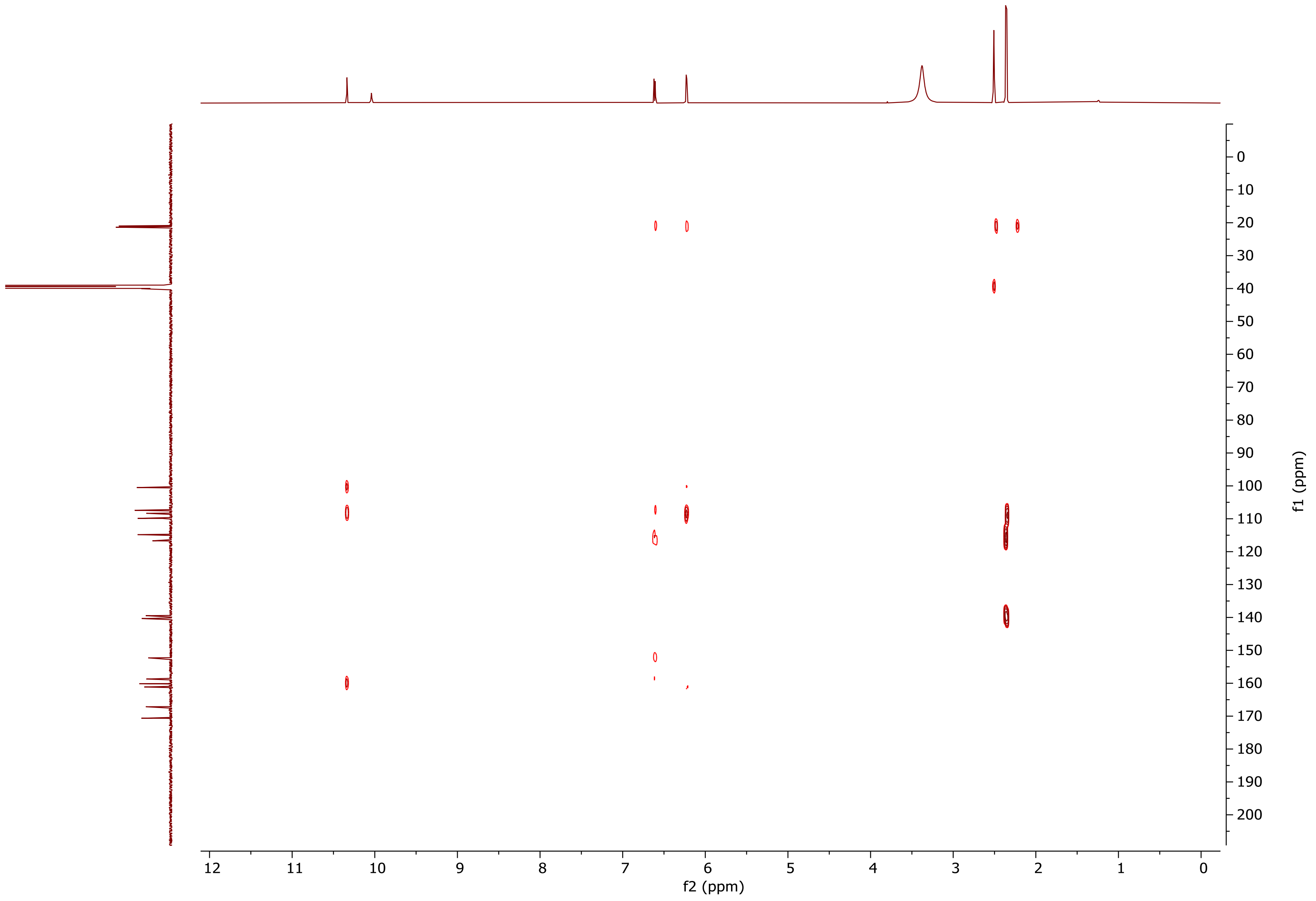

COSY spectrum of lecanoric acid (**1**) in DMSO-d_6

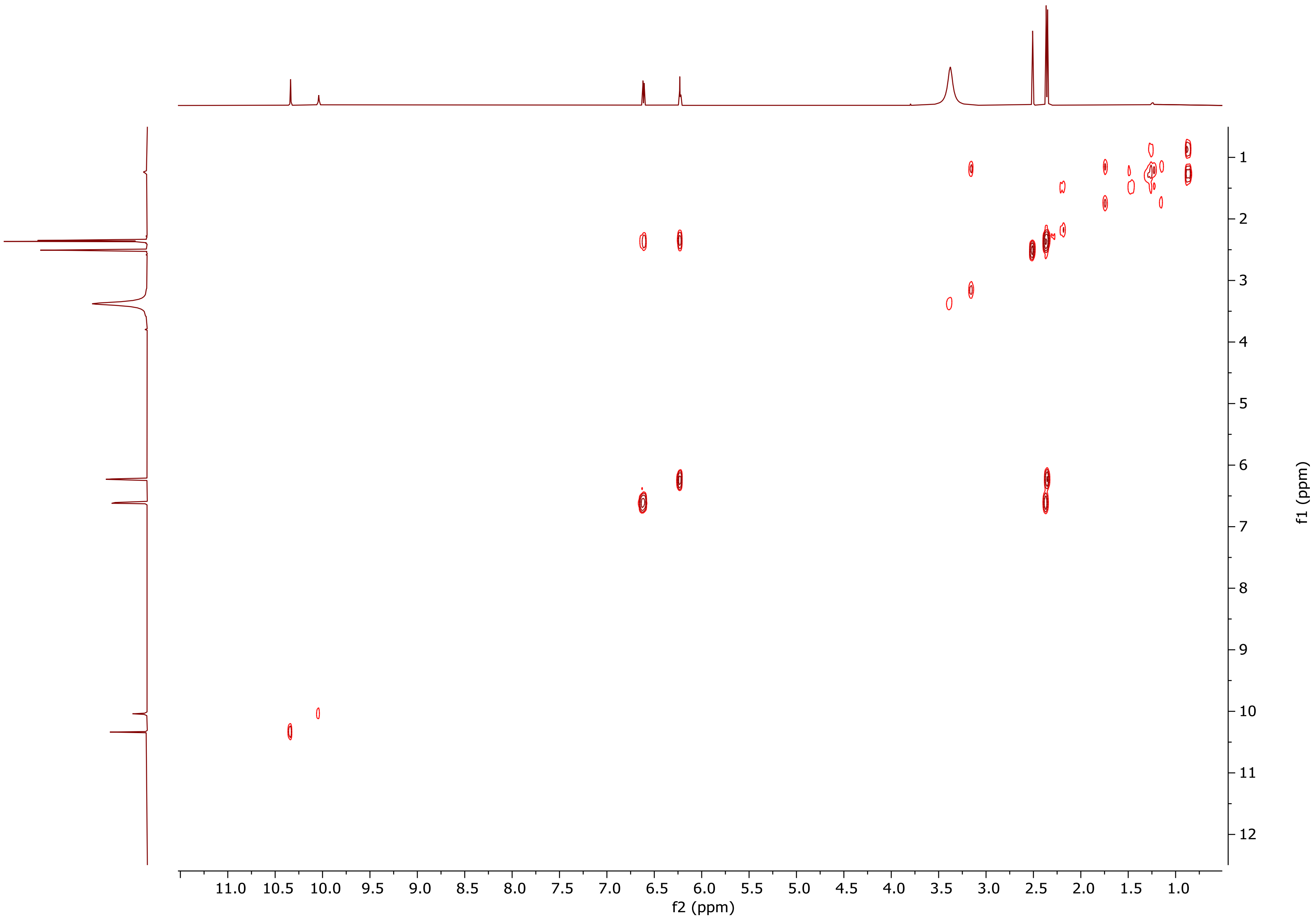

ROESY spectrum of lecanoric acid (**1**) in DMSO-d_6

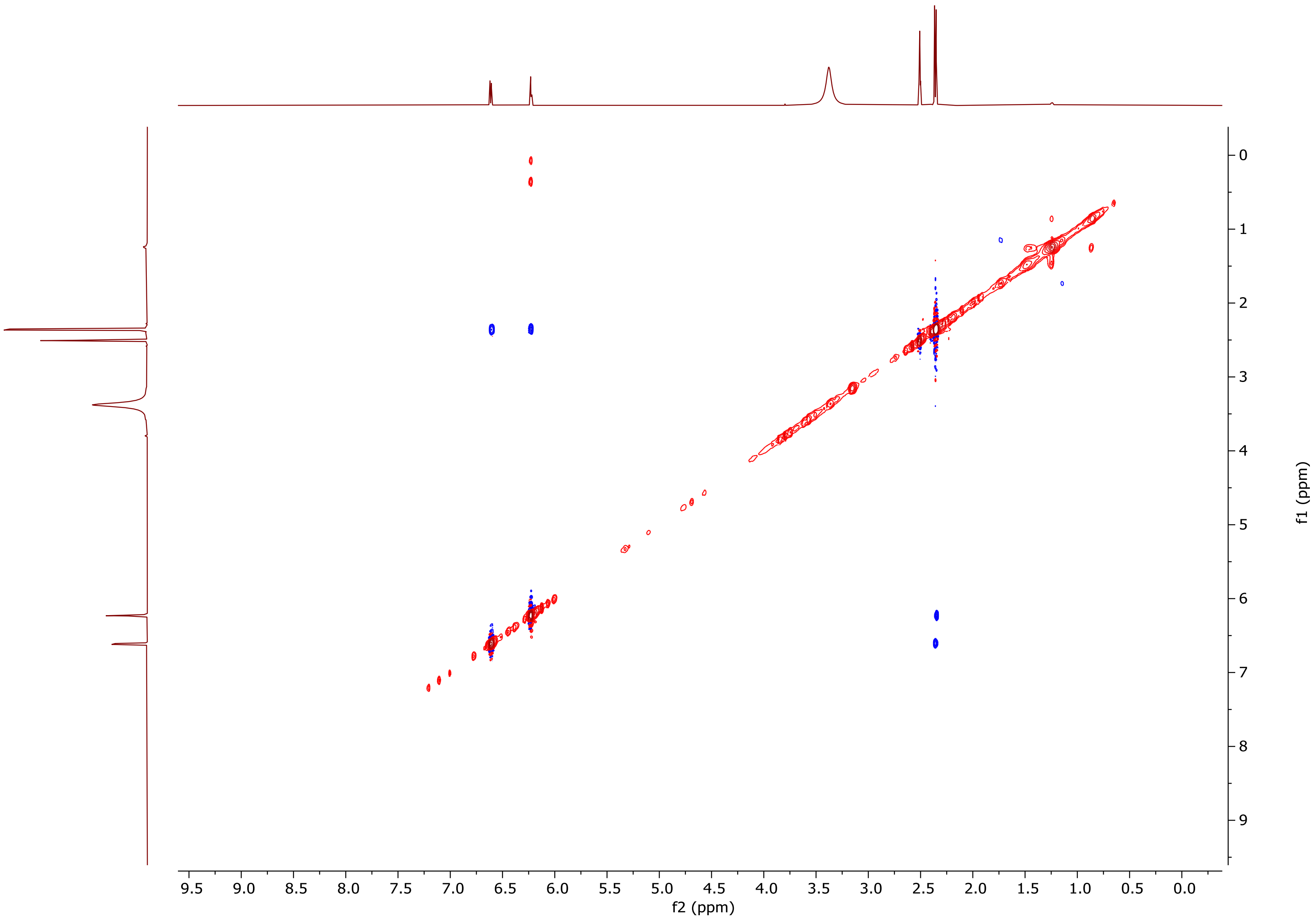

NMR data table for lecanoric acid (**1**) in DMSO-d_6[a]

Position	δ_H (mult., *J* in Hz)	δ_C, type	COSY	HMBC	ROESY
1		108.4, C			
2		160.1, C			
2-OH	10.34 (s)			1,2,3,4,5	
3	6.23 (d, 2.3)	100.5, CH	5,6-Me[w]	1,2,4,5,6-Me	
4		161.1, C			
4-OH	10.04 (brs)				
5	6.22 (dq, 2.3, 0.7)	109.9, CH	3,6-Me	1,2,3,4,6-Me	6-Me
6		140.3, C			
6-Me	2.35 (s)	21.4, CH_3	3[w],5	1,2[w],5,6,7[w]	5
7		167.2, C			
1’		116.7, C			
2’		158.7, C			
2’-OH	[b]				
3’	6.62 (d, 2.3)	107.4, CH	5’,6’-Me[w]	1’,2’,4’,5’	
4’		152.3, C			
5’	6.60 (dq, 2.3, 0.7)	114.8, CH	3’,6’-Me	1’,3’,4’,6’-Me	6’-Me
6’		139.5, C			
6’-Me	2.37 (s)	21.0, CH_3	3’[w],5’	1’,2’[w],5’,6’,7’[w]	5’
7’		170.6, C			
7’-OH	[b]				

[a] Spectra recorded at 25 °C (500 MHz for ^{1}H NMR and 125 MHz for ^{13}C NMR); [b] Not observed; [w] Weak correlation.

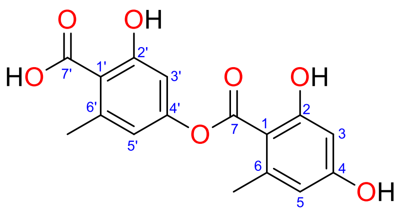

^{1}H NMR spectrum of divaricatic acid (**2**) in DMSO-d_6

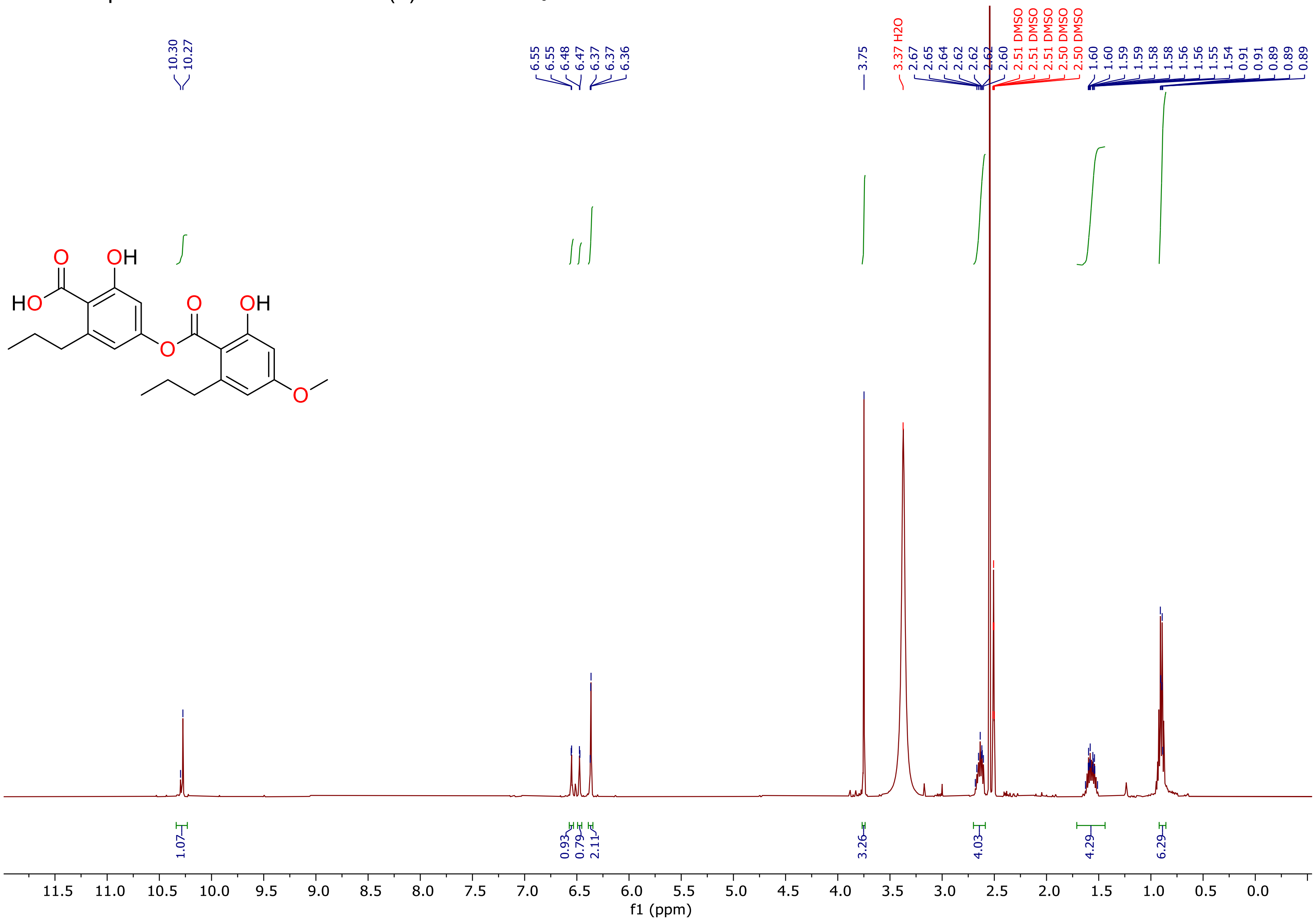

^{13}C NMR spectrum of divaricatic acid (**2**) in DMSO-d_6

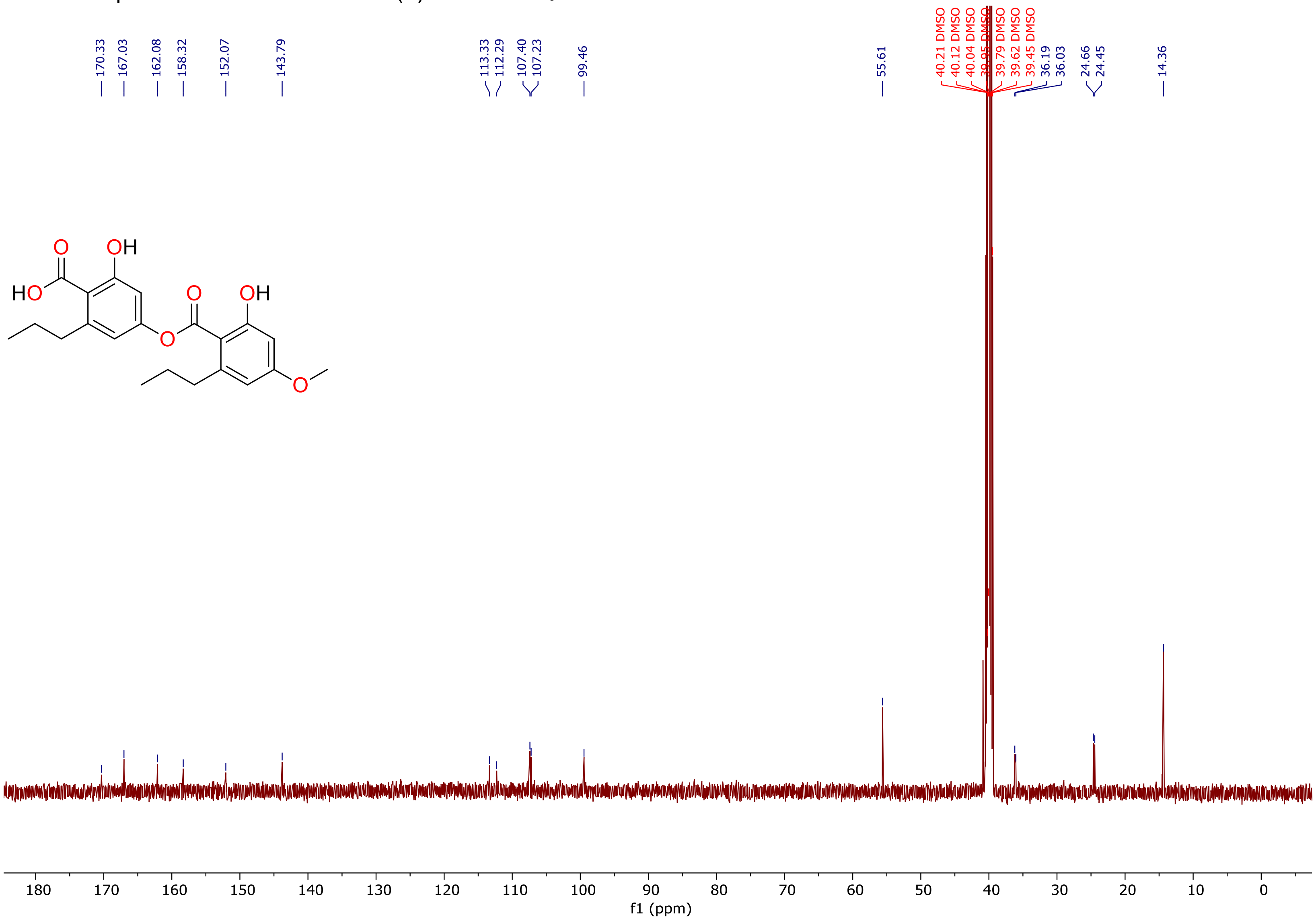

NMR data table for divaricatic acid (**2**) in DMSO-d_6[a]

Position	δ_H (mult., *J* in Hz)	δ_C, type
1		107.2, C
2		[b]
2-OH	10.30 (s)	
3	6.47 (d, 2.2)	99.5, CH
4		152.1, C
4-OMe	3.75 (s)	55.6, CH_3
5	6.55 (d, 2.2)	113.3, CH
6		[b]
7		167.0, C
1′		112.3, C
2′		158.3, C
2′-OH	10.27 (s)	
3′	6.36 (d, 2.4)	107.4, CH
4′		162.1, C
5′	6.37 (d, 2.4)	[b]
6′		143.8, C
7′		170.3, C
1′′	2.60 (t, 7.8)	36.0, CH_2
2′′	1.51–1.63 (m)	24.5, CH_2
3′′	0.89 (t, 7.3)	14.4, CH_3
1′′′	2.64 (t, 7.8)	36.2, CH_2
2′′′	1.51–1.63 (m)	24.7, CH_2
3′′′	0.91 (t, 7.3)	14.4, CH_3

[a] Spectra recorded at 25 °C (500 MHz for ^{1}H NMR and 125 MHz for ^{13}C NMR); [b] Not observed.

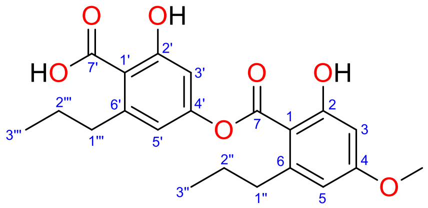

^{1}H NMR spectrum of orcinol (**3**) in CD_3OD

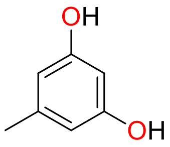

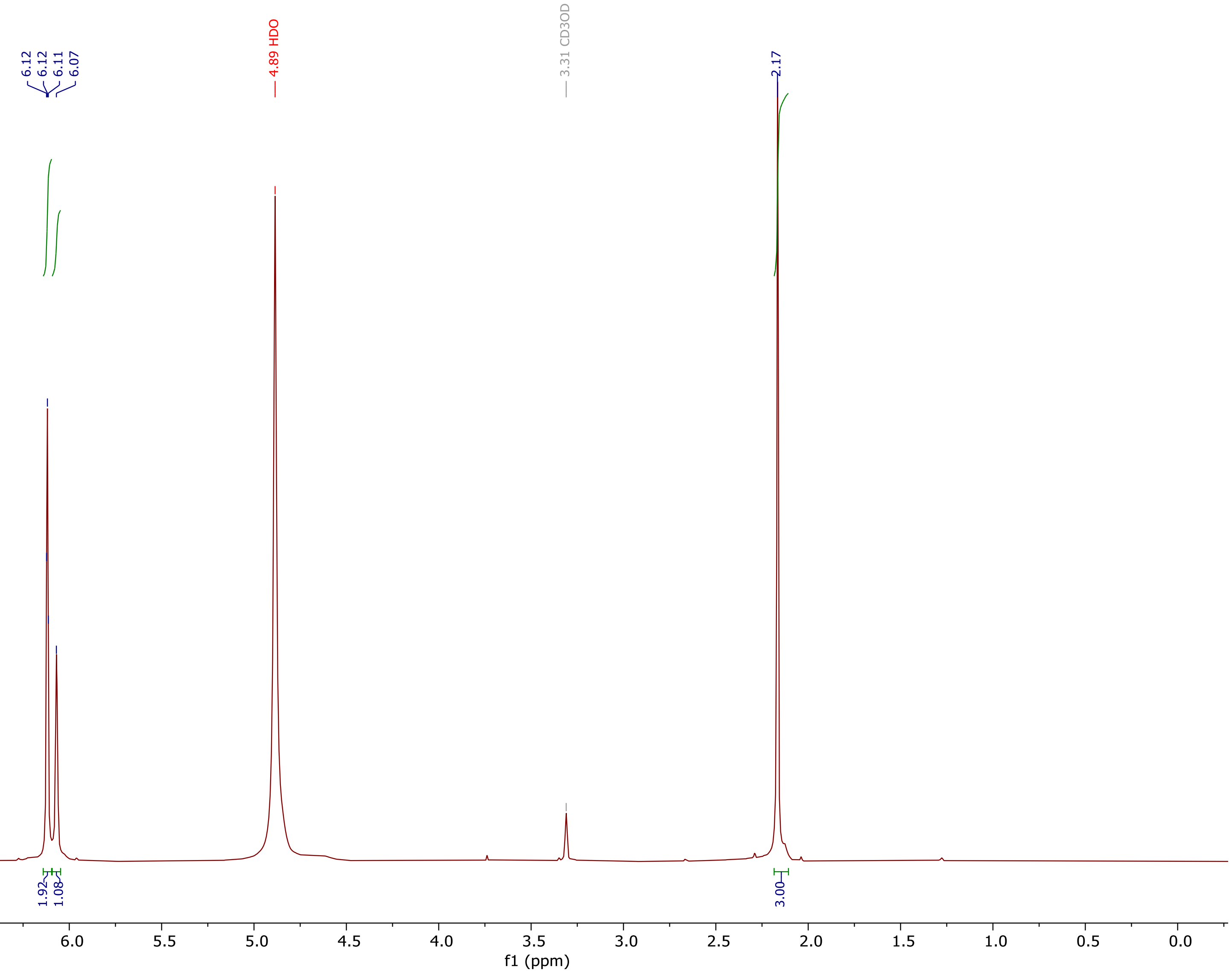

^{13}C NMR spectrum of orcinol (**3**) in CD_3OD

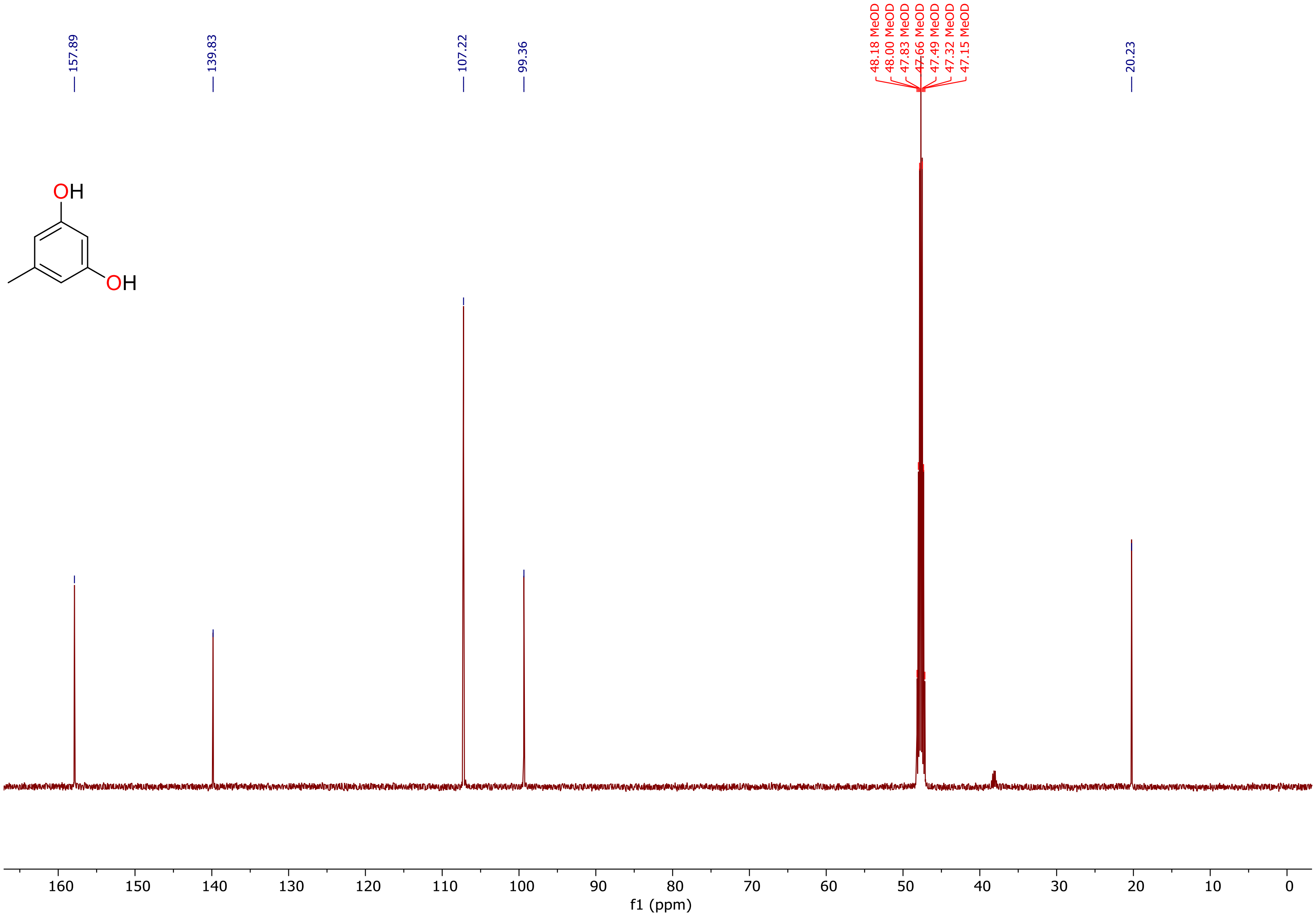

NMR data table for orcinol (**3**) in CD_3OD^a

Position	δ_H (mult., *J* in Hz)	δ_C, type
1	6.12, d (2.4)	107.2, CH
2		157.9, C
2-OH		
3	6.07, t (2.3)	99.4, CH
4		157.9, C
4-OH		
5	6.12, d (2.4)	107.2, CH
6		139.8, C
6-Me	2.17 (s)	20.2, CH_3

[a] Spectra recorded at 25 °C (500 MHz for ^{1}H NMR and 125 MHz for ^{13}C NMR).

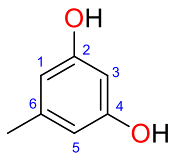

^{1}H NMR spectrum of orsellinic acid (**4**) in DMSO-d_6

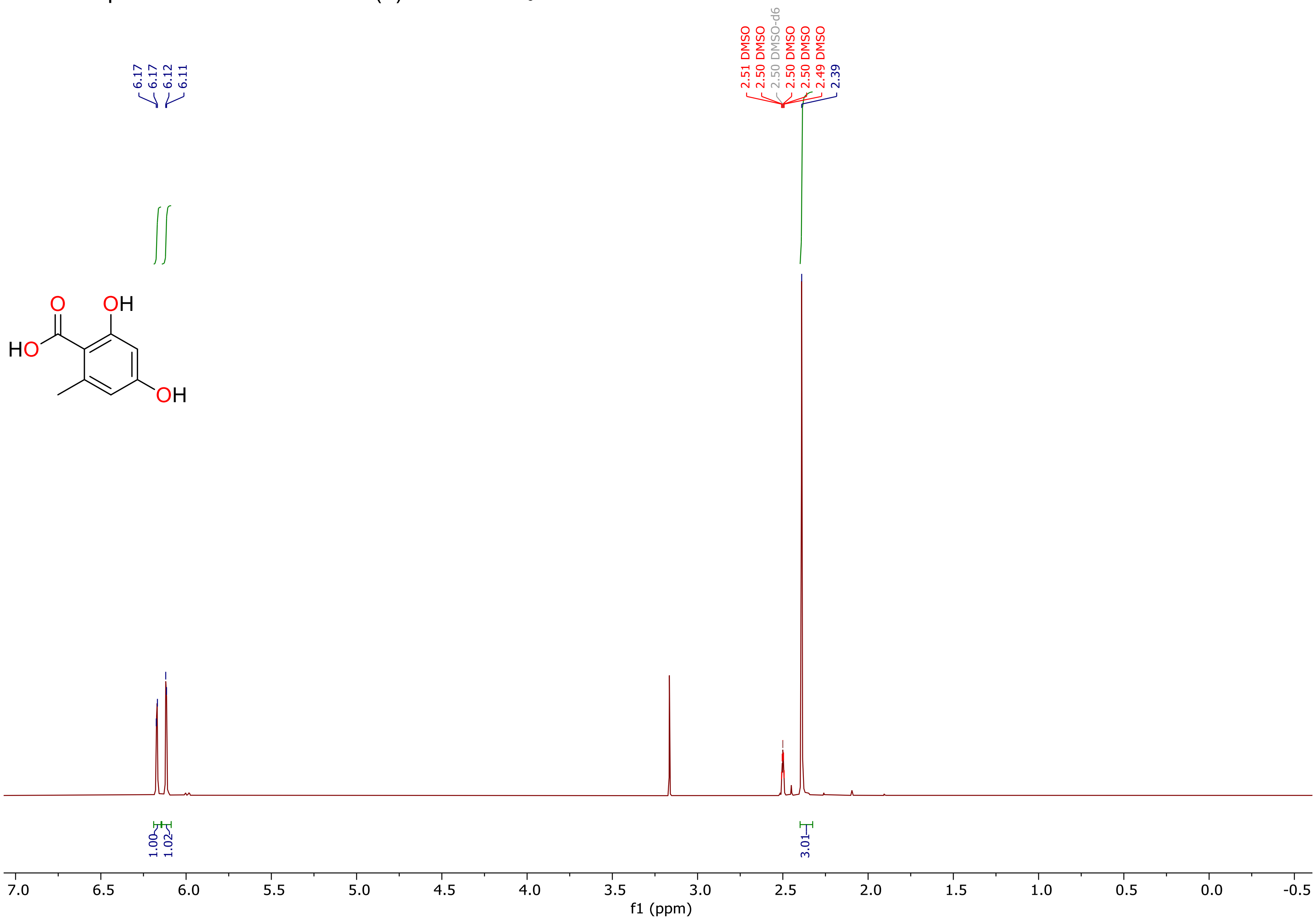

^{13}C NMR spectrum of orsellinic acid (**4**) in DMSO-d_6

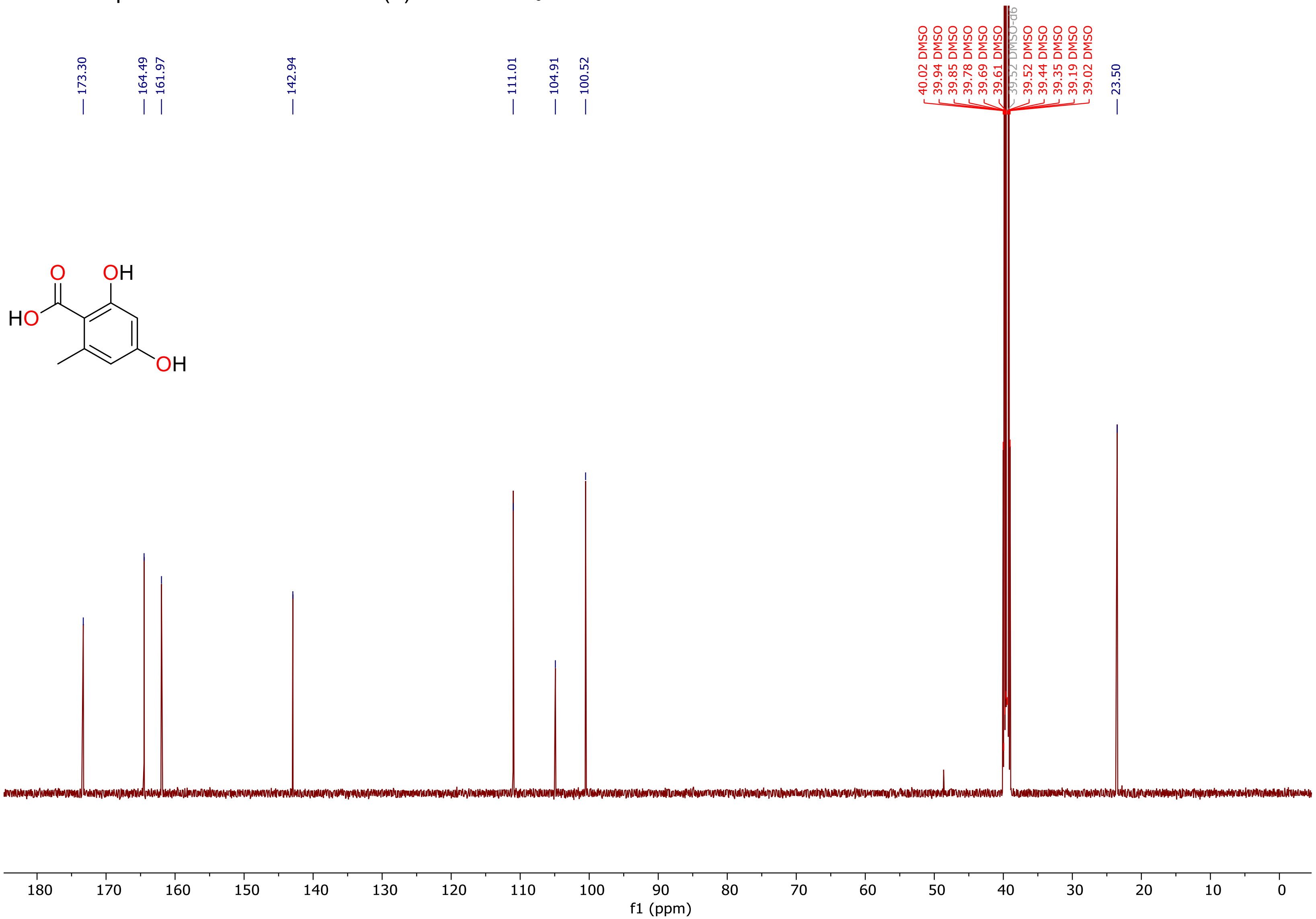

NMR data table for orsellinic acid (**4**) in DMSO-d_6[a]

Position	δ_H (mult., J in Hz)	δ_C, type
1		104.9, C
2		162.0, C
2-OH	[b]	
3	6.17 (d, 2.4)	100.5, CH
4		164.5, C
4-OH	[b]	
5	6.12 (d, 2.4)	111.0, CH
6		142.9, C
6-Me	2.39 (s)	23.5, CH_3
7		173.3, C
7-OH	[b]	

[a] Spectra recorded at 25 °C (500 MHz for ^{1}H NMR and 125 MHz for ^{13}C NMR); [b] Not observed.

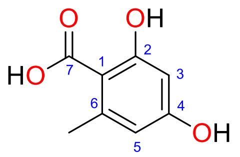

^{1}H NMR spectrum of methyl orsellinate (**5**) in $CDCl_3$

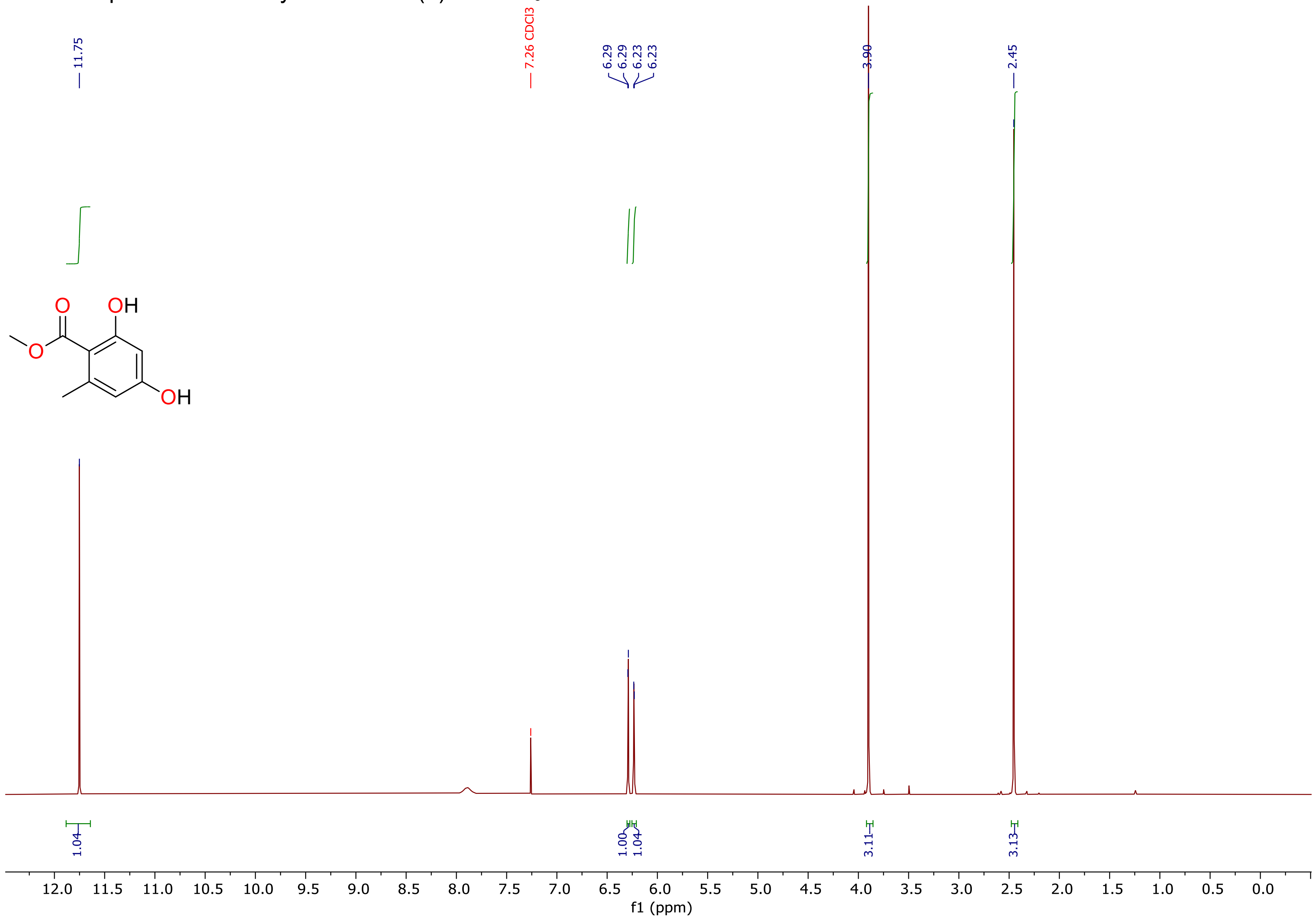

^{13}C NMR spectrum of methyl orsellinate (**5**) in $CDCl_3$

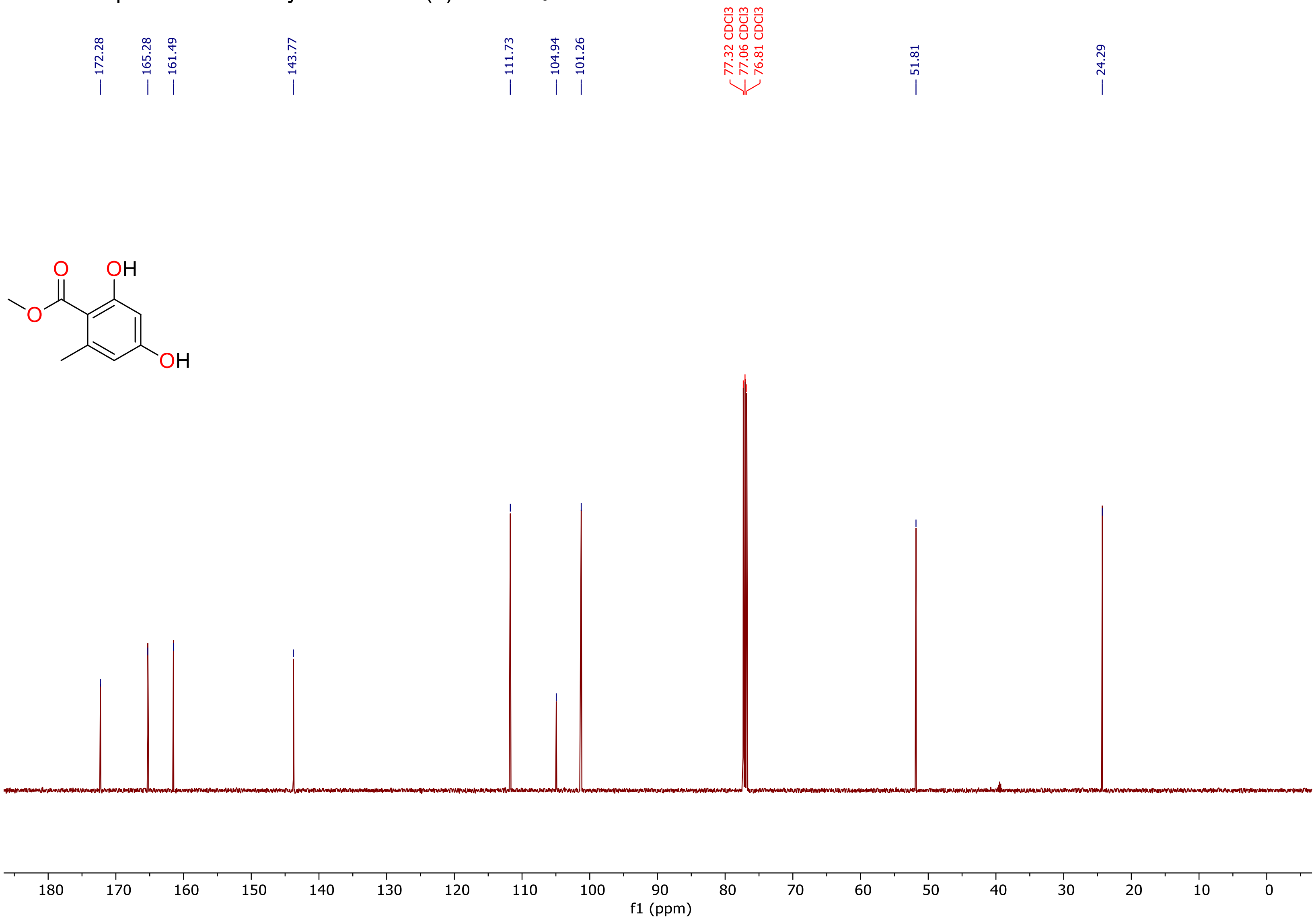

NMR data table for methyl orsellinate (**5**) in $CDCl_3$[a]

Position	δ_H (mult., *J* in Hz)	δ_C, type
1		101.3, C
2		161.5, C
2-OH	11.75 (s)	
3	6.29 (d, 2.5)	104.9, CH
4		165.3, C
4-OH	[b]	
5	6.23 (d, 2.5)	111.7, CH
6		143.8, C
6-Me	2.45 (s)	24.3, CH_3
7		172.3, C
7-OMe	3.90 (s)	51.8, CH_3

[a] Spectra recorded at 25 °C (500 MHz for ^{1}H NMR and 125 MHz for ^{13}C NMR); [b] Not observed.

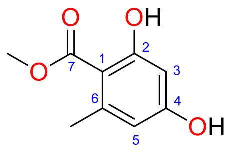

^{1}H NMR spectrum of compound **6** in DMSO-d_6

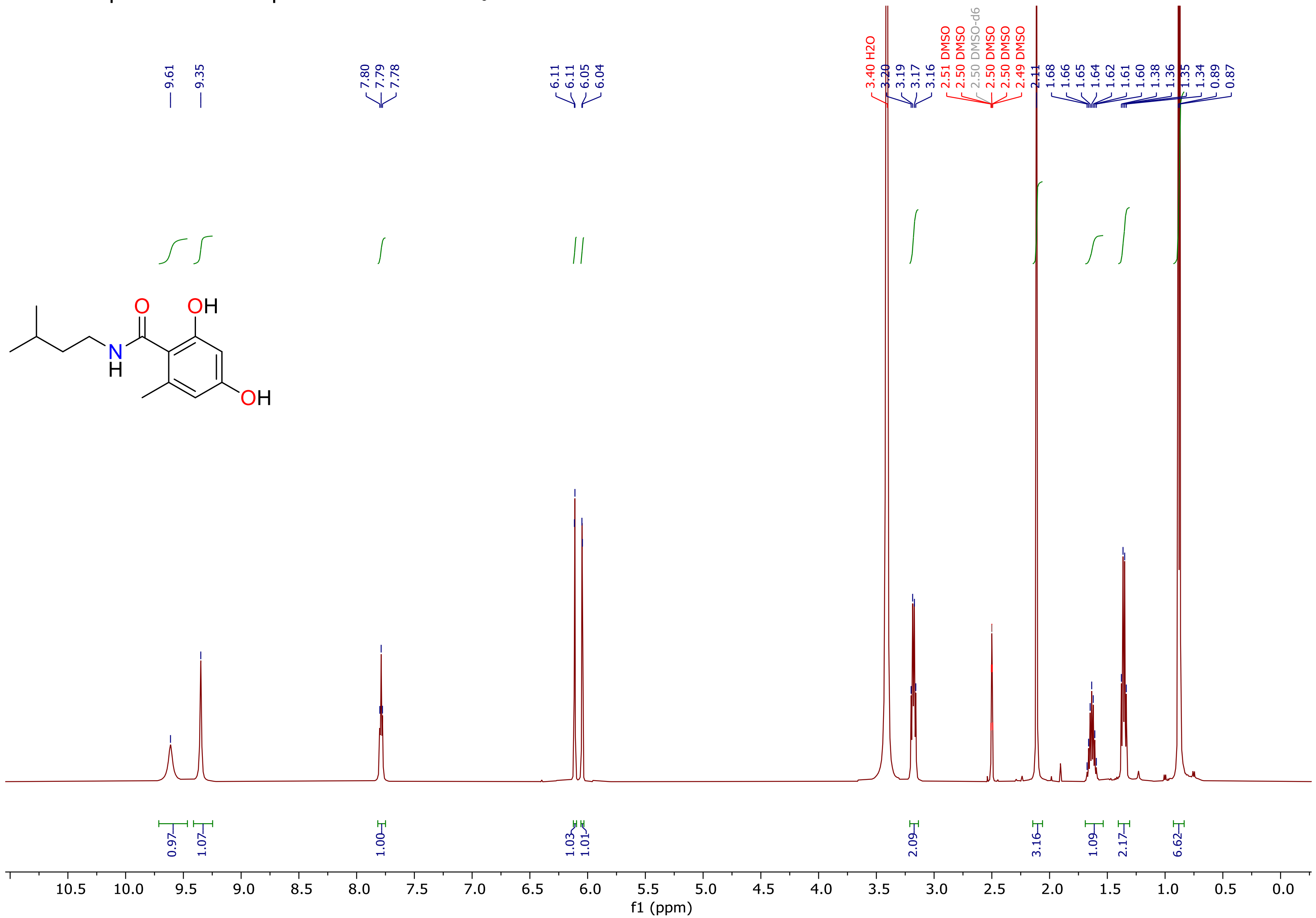

^{1}H NMR spectrum of compound **6** in DMSO-d_6 and D_2O

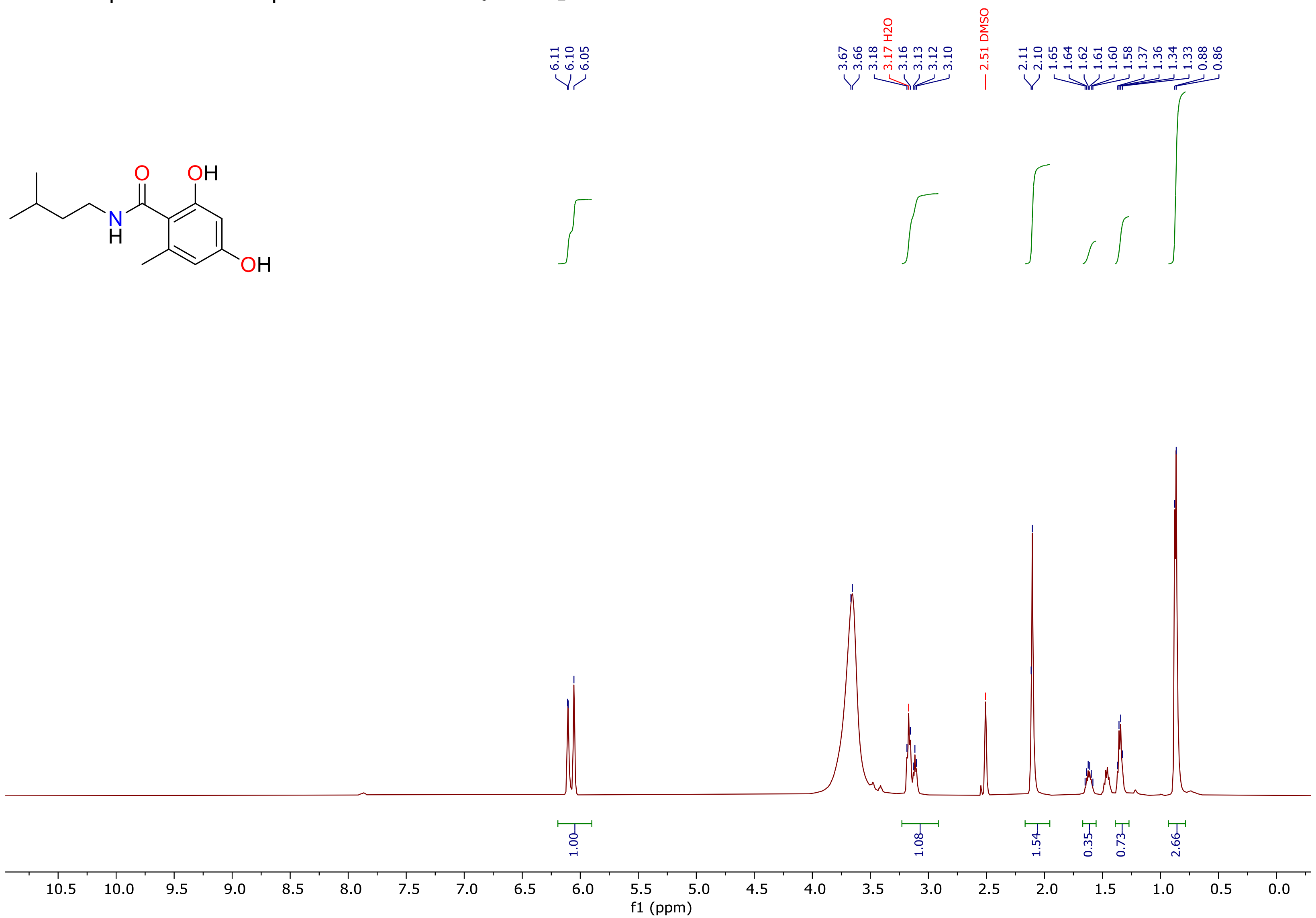

^{13}C NMR spectrum of compound **6** in DMSO-d_6

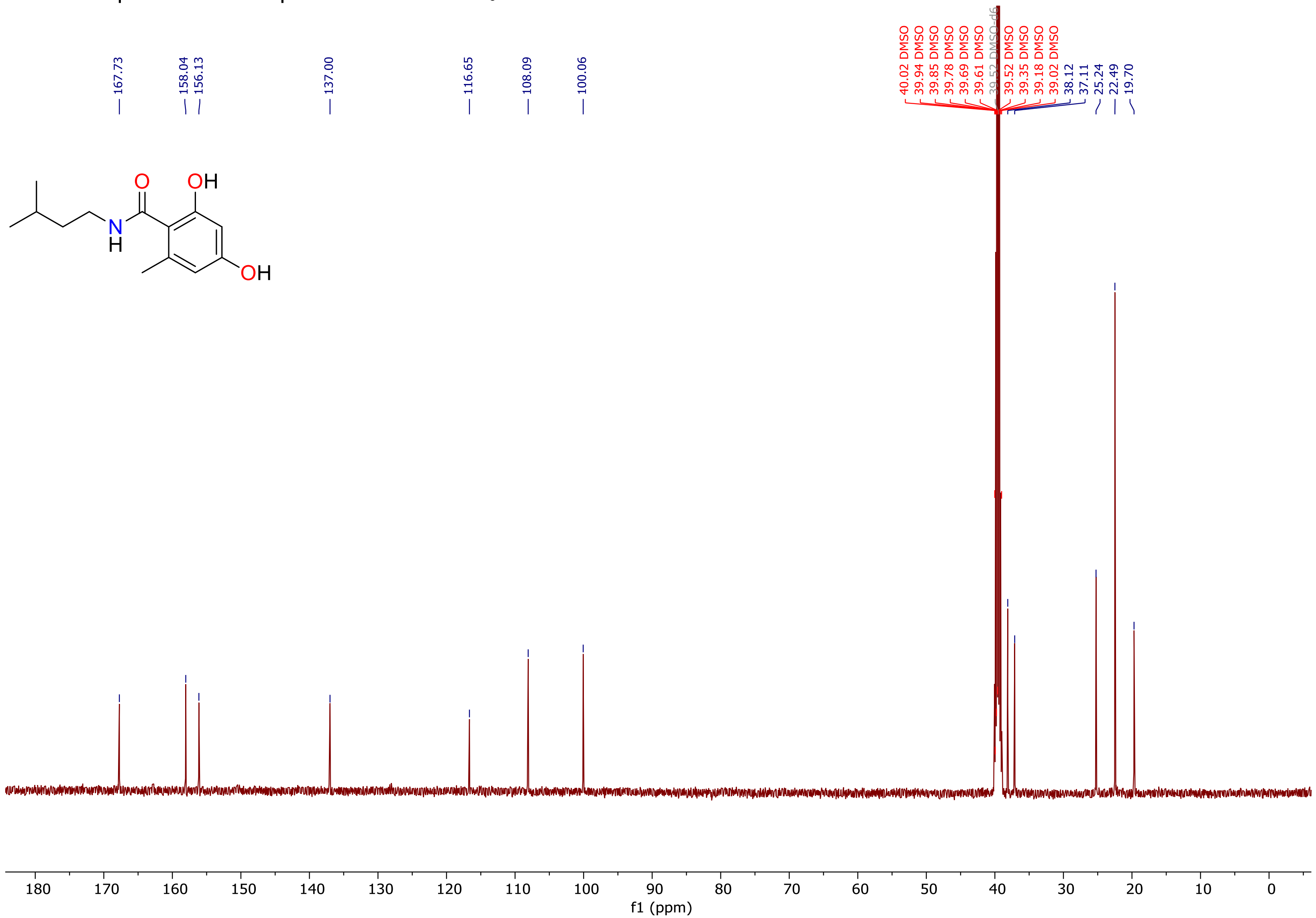

HSQC spectrum of compound **6** in DMSO-d_6

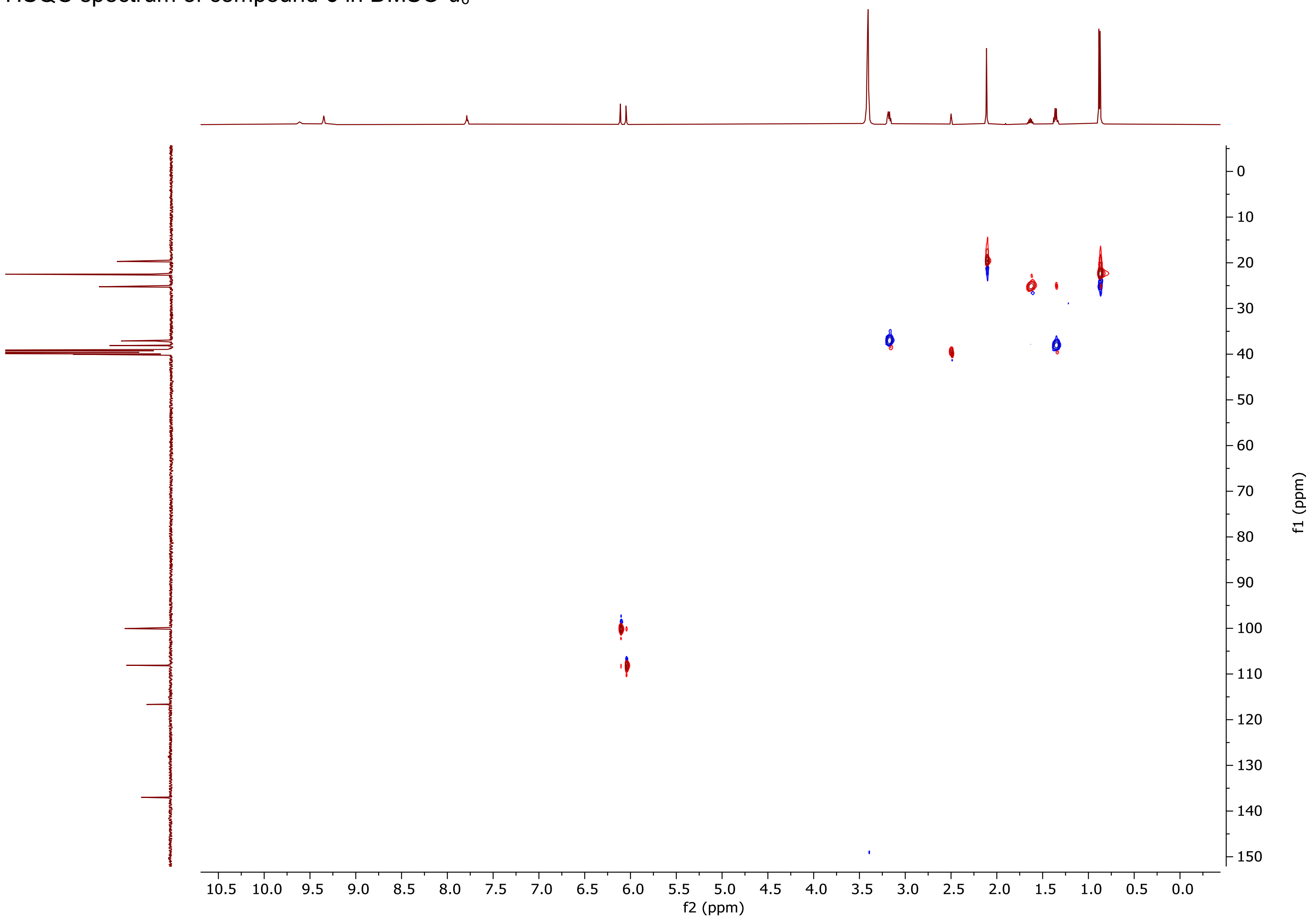

HMBC spectrum of compound **6** in DMSO-d_6

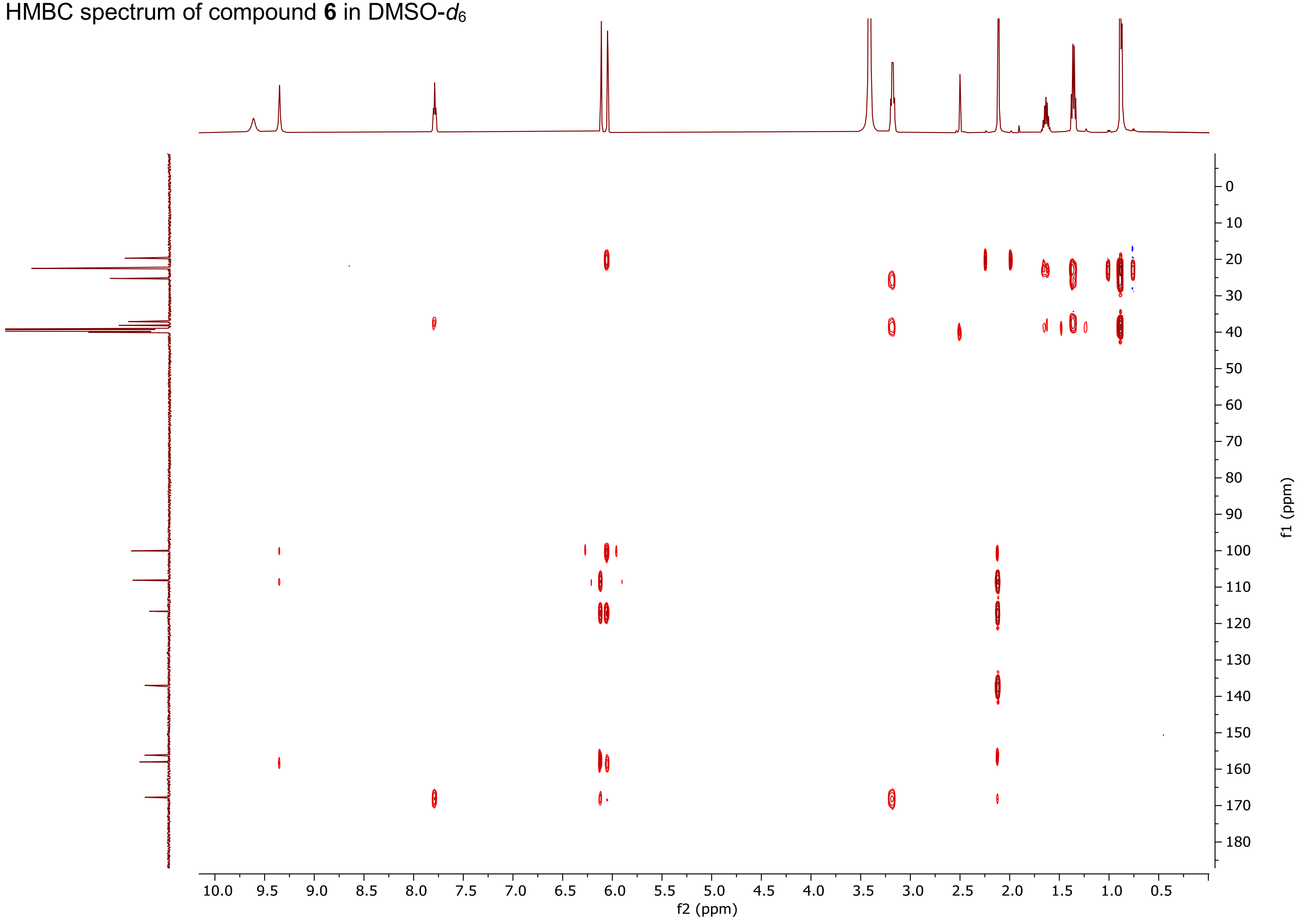

COSY spectrum of compound **6** in DMSO-d_6

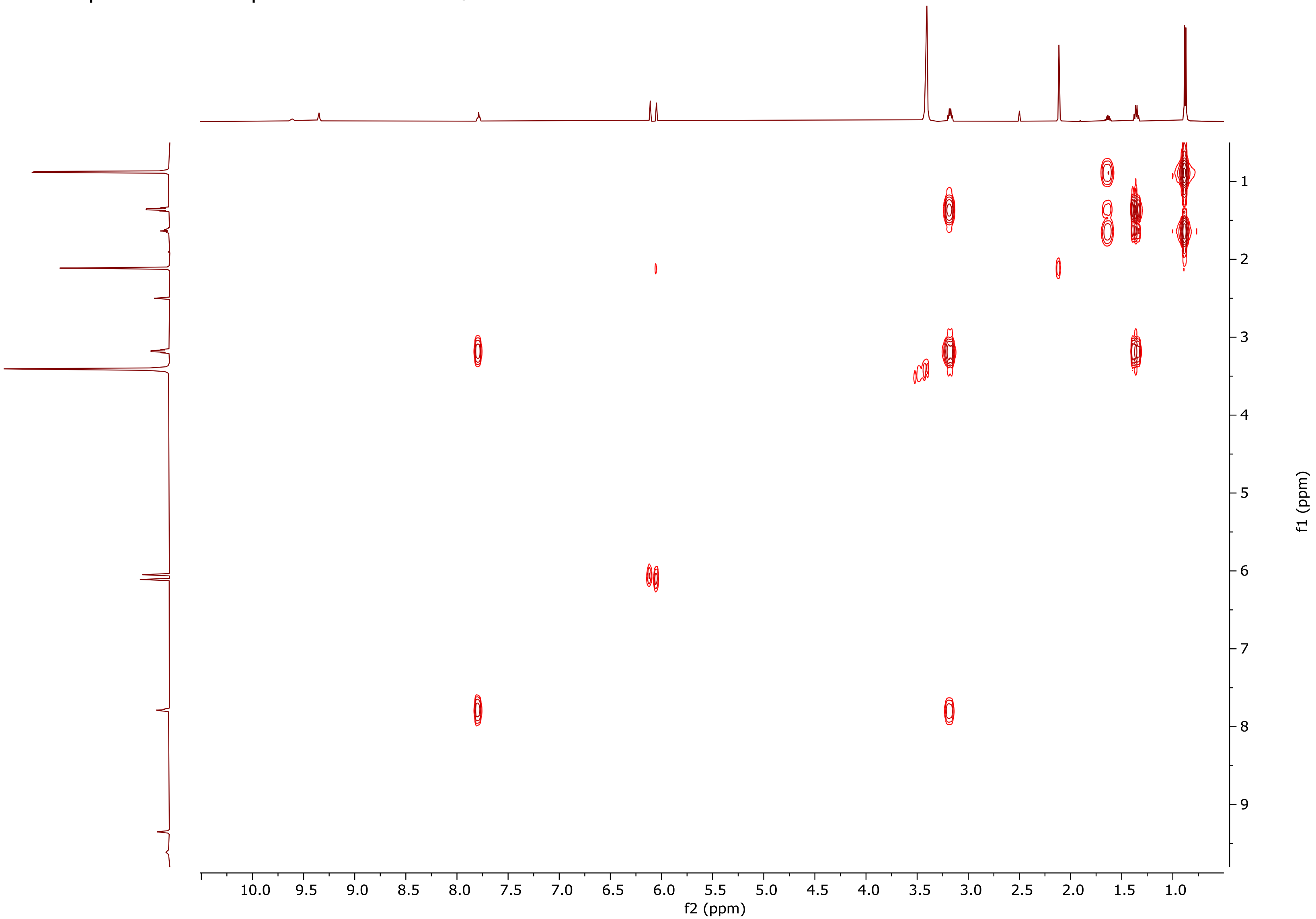

ROESY spectrum of compound **6** in DMSO-d_6

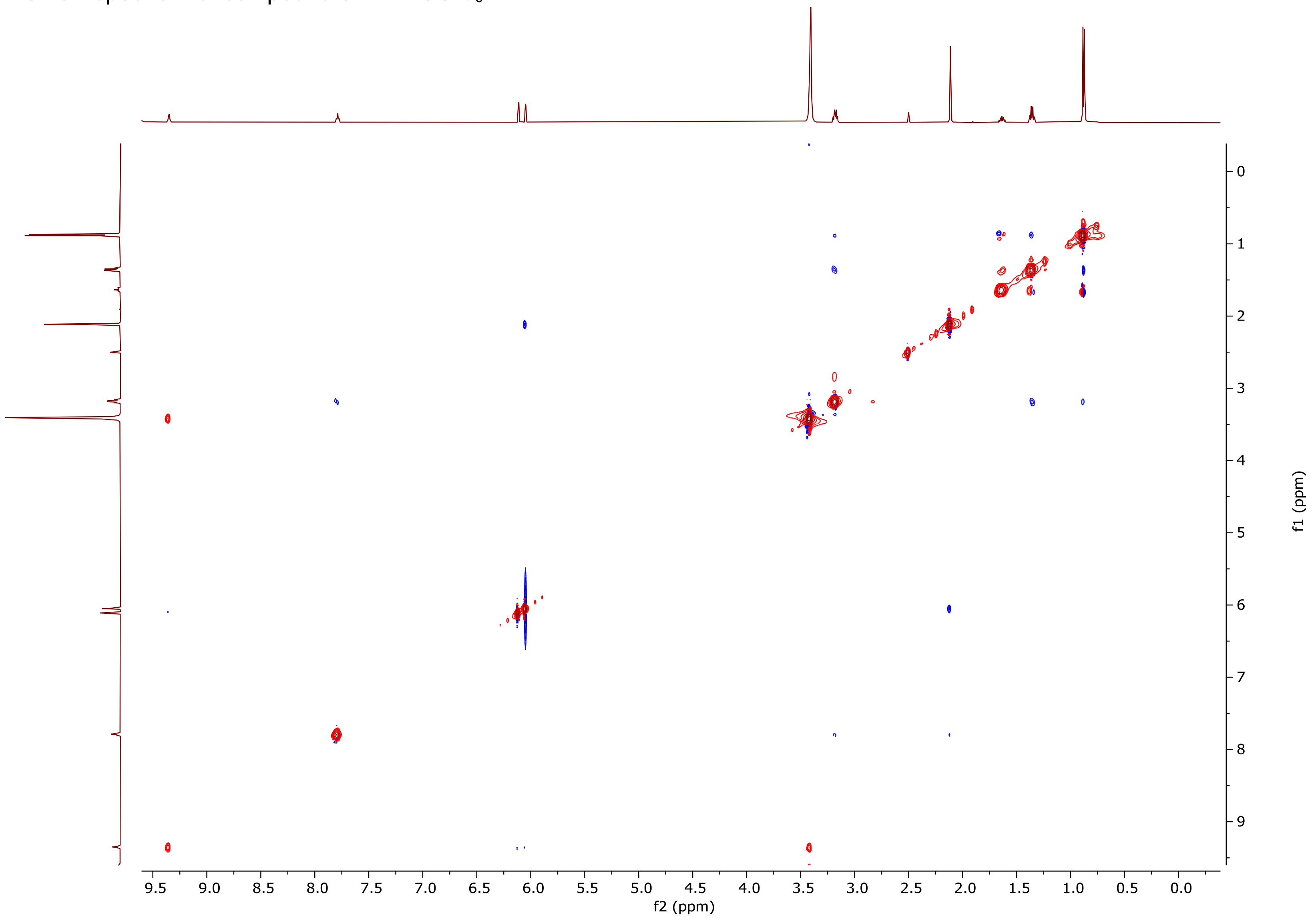

NMR data table for compound **6** in DMSO-d_6[a]

Position	δ_H (mult., *J* in Hz)	δ_C, type	COSY	HMBC	ROESY
1		116.7, C			
2		156.1, C			
2-OH	9.61 (brs)				3
3	6.11 (d, 2.2)	100.1, CH	5,6-Me^w	1,4,5,7^w	2-OH,4-OH
4		158.0, C			
4-OH	9.35 (brs)			3,4,5	3,5
5	6.04 (d, 2.2)	108.1, CH	3,6-Me^w	1,3,4,6-Me,7^w	4-OH,6-Me
6		137.0, C			
6-Me	2.11 (s)	19.7, CH_3	3^w,5^w	1,2^w,3^w,4,5,6,7^w	5,7-NH
7		167.7, C			
7-NH	7.79 (t, 5.6)		8	7,8,9	6-Me,8,9
8	3.17 (dt, 5.6, 6.6)	37.1, CH_2	7-NH,9	7,8,9,10	7-NH,9,11,12
9	1.35 (dt, 6.6, 7.1)	38.1, CH_2	8,10	8,10,11,12	8,11,12
10	1.64 (m)	25.2, CH	9,11,12	9,11,12	9,11,12
11	0.87 (d, 6.6)	22.5, CH_3	10	9,10,12	8,9,10
12	0.89 (d, 6.6)	22.5, CH_3	10	9,10,11	8,9,10

[a] Spectra recorded at 25 °C (500 MHz for ^{1}H NMR and 125 MHz for ^{13}C NMR); [w] Weak correlation.

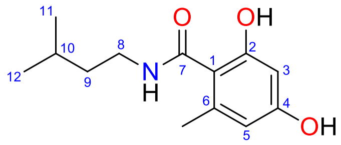

High resolution mass spectrum of compound **6**

Mass Spectrum SmartFormula Report

Analysis Info

Analysis Name	D:\Data\Sasha\20231004\RAD915C000001.d	Acquisition Date	10/4/2023 1:55:33 PM
Method	DirectInfusion_2018_pos.m	Operator	Demo User
Sample Name	RAD915C	Instrument	maXis II ETD
Comment			

Acquisition Parameter

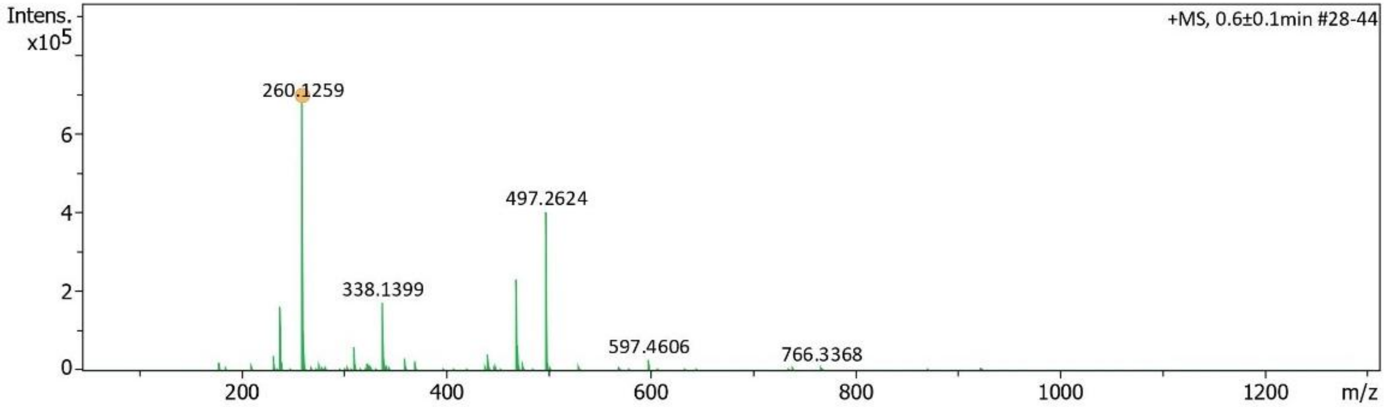

Meas. m/z	#	Ion Formula	m/z	err [ppm]	mSigma	# mSigma	Score	rdb	e¯ Conf	N-Rule
260.1259	1	C13H19NNaO3	260.1257	-0.7	0.6	1	100.00	5.0	even	ok

^{1}H NMR spectrum of compound **7** in DMSO-d_6

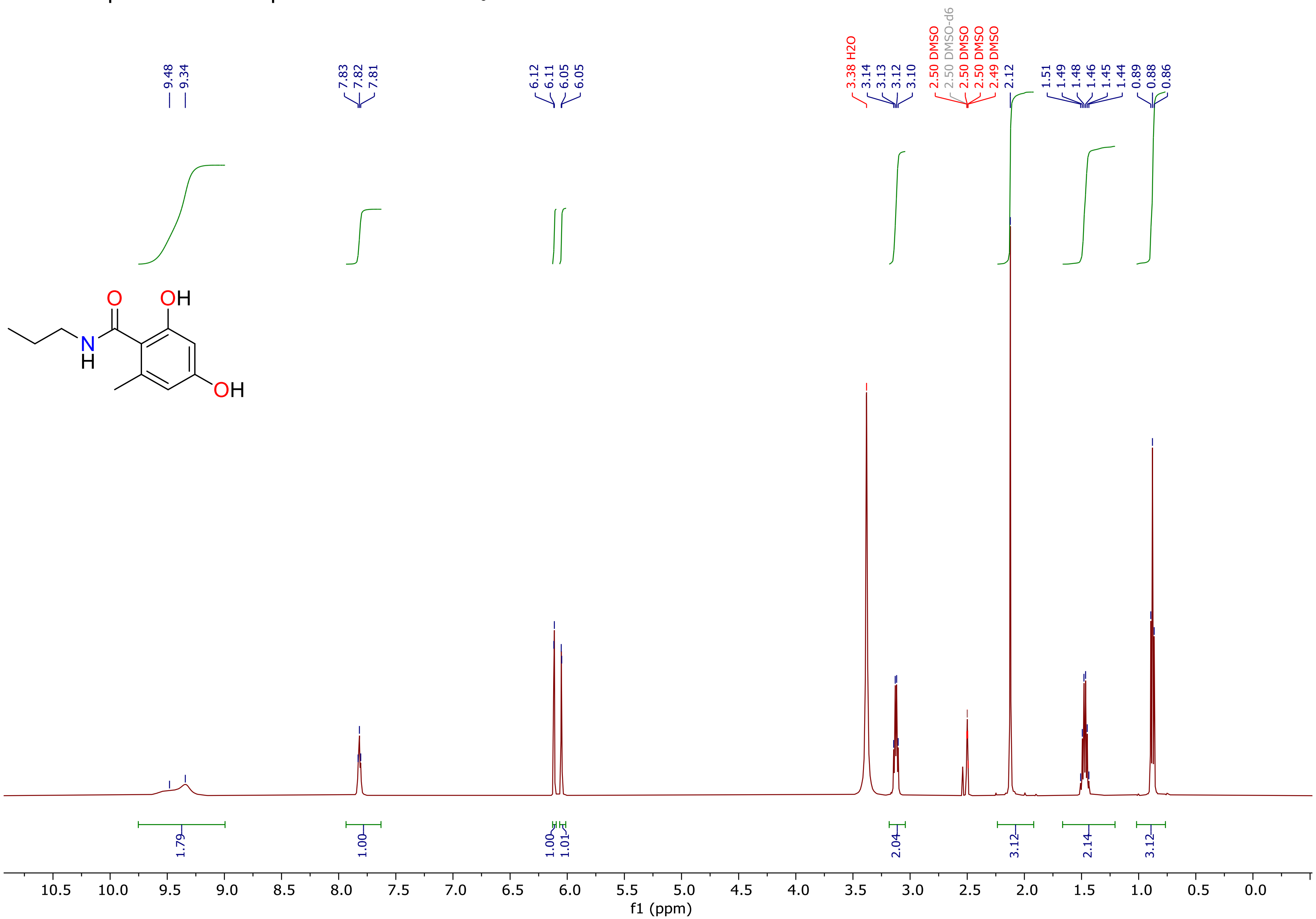

^{13}C NMR spectrum of compound **7** in DMSO-d_6

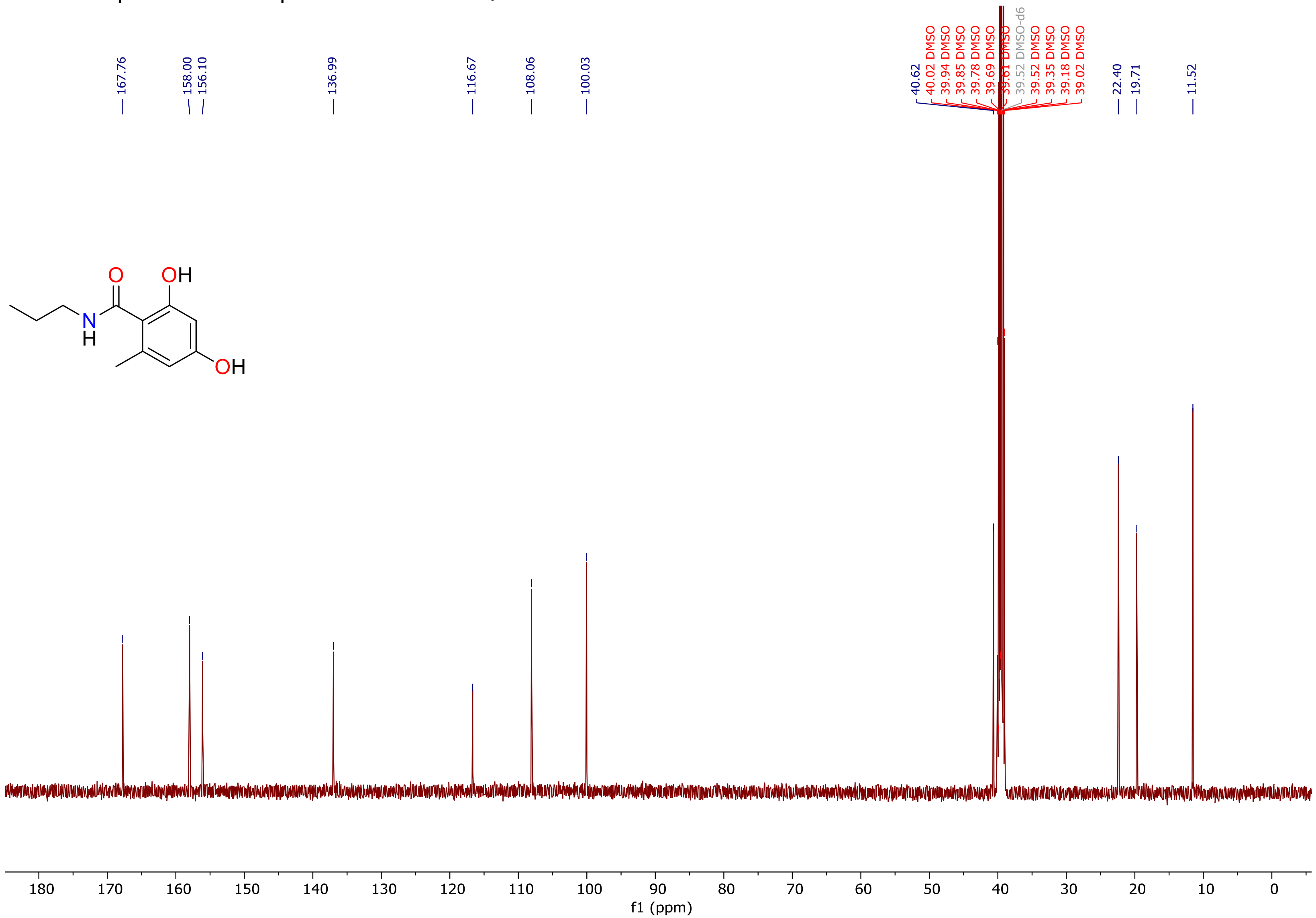

HSQC spectrum of compound **7** in DMSO-d_6

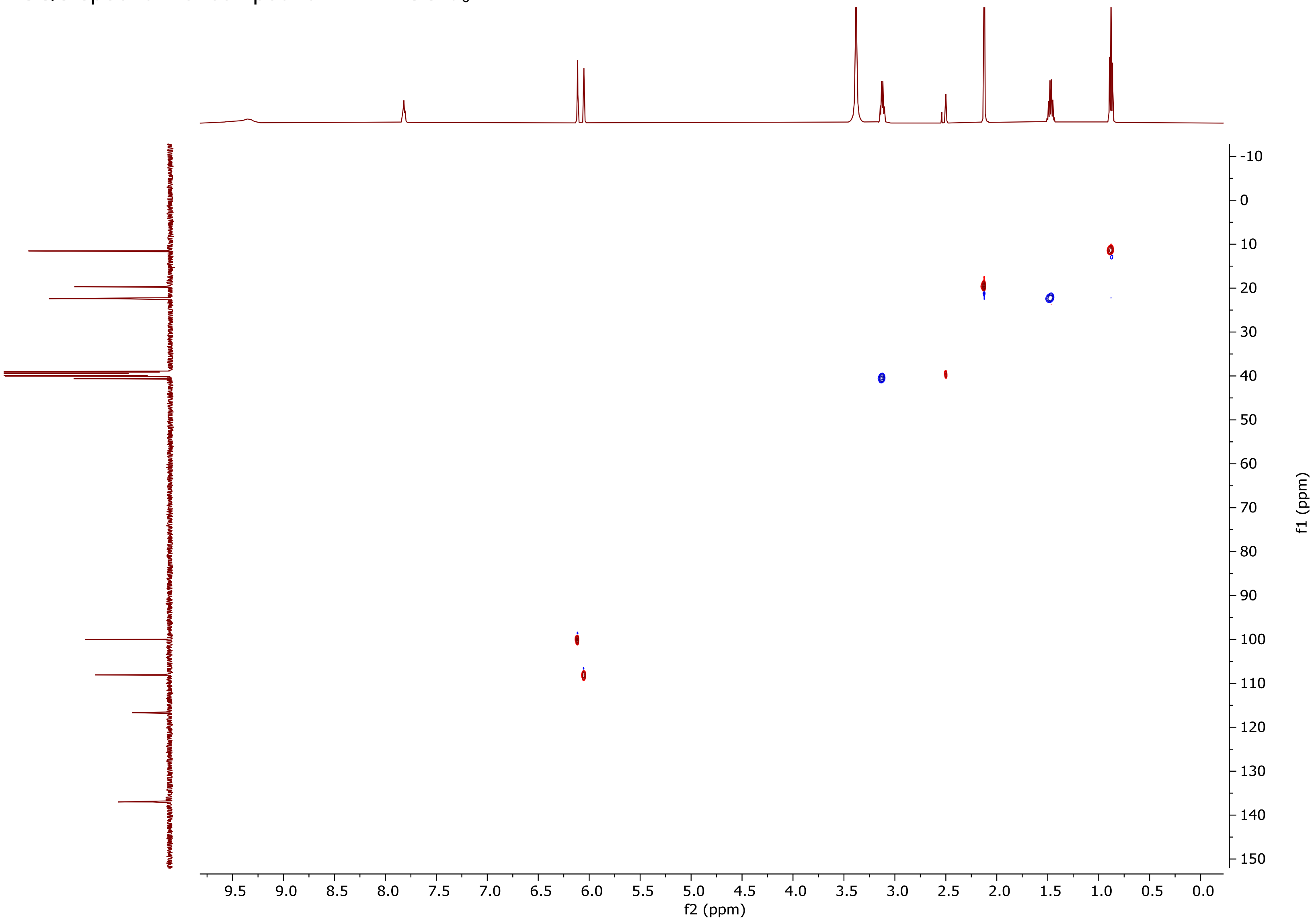

HMBC spectrum of compound **7** in DMSO-d_6

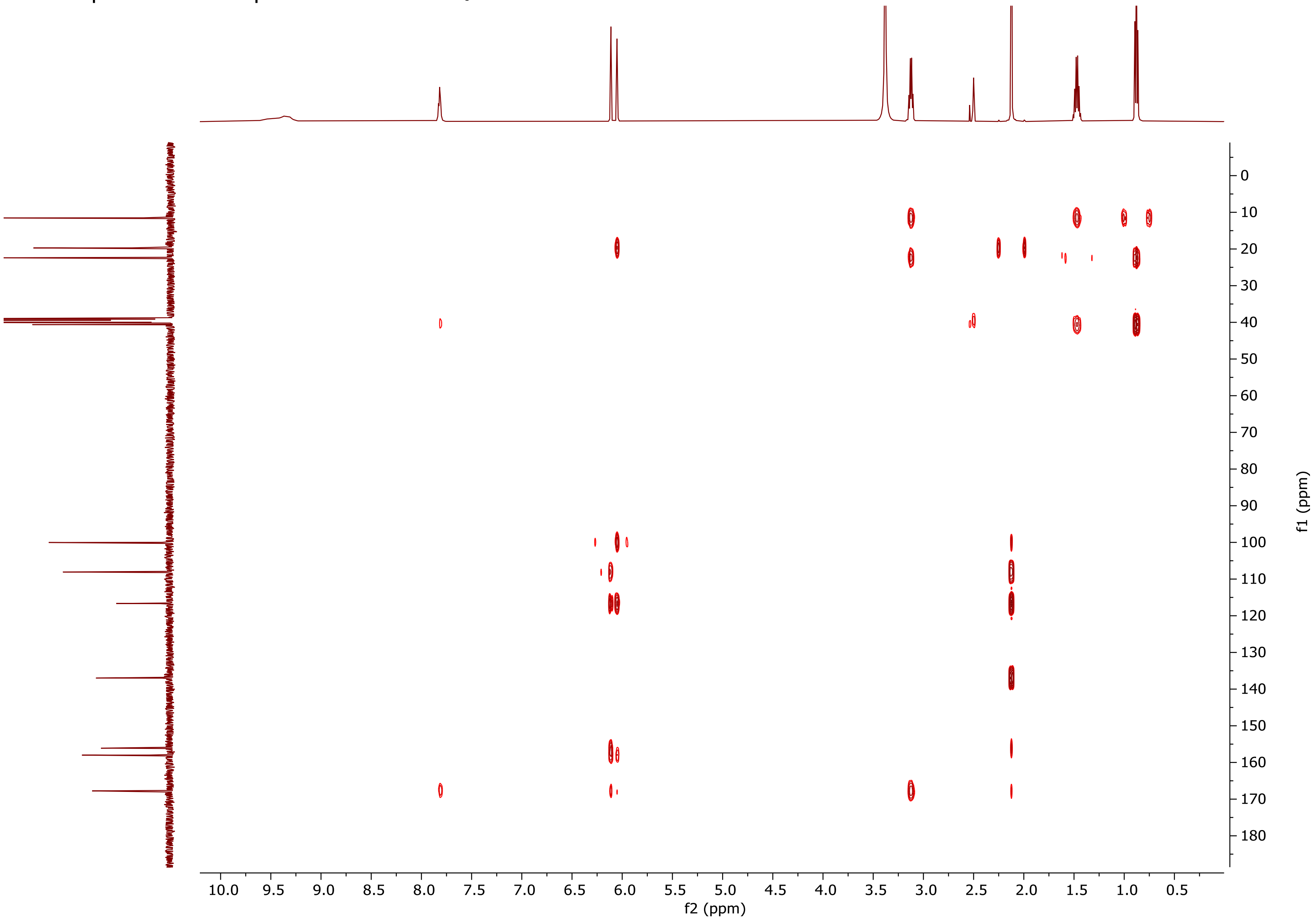

COSY spectrum of compound **7** in DMSO-d_6

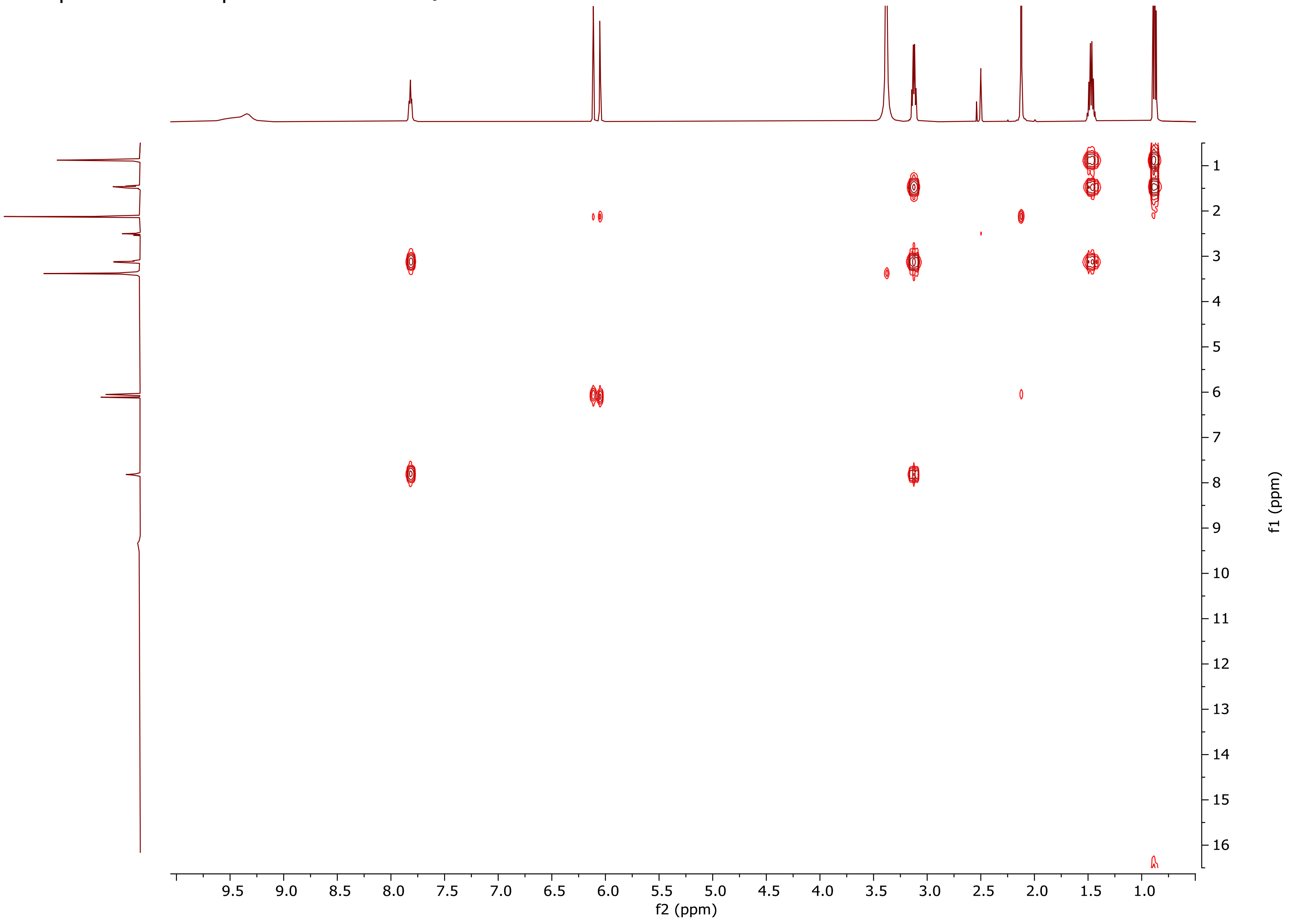

ROESY spectrum of compound **7** in DMSO-d_6

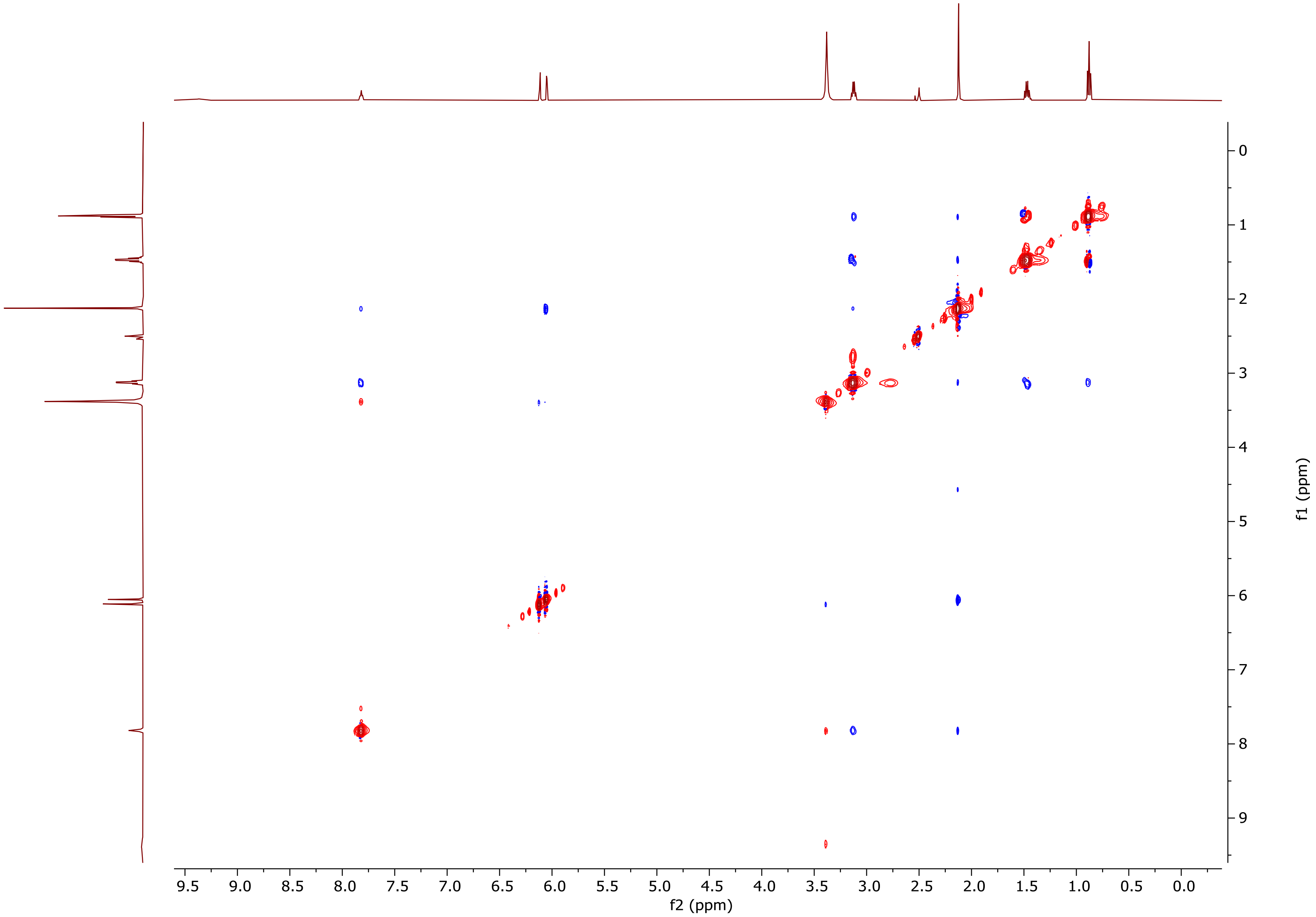

NMR data table for compound **7** in DMSO-d_6[a]

Position	δ_H (mult., *J* in Hz)	δ_C, type	COSY	HMBC	ROESY
1		116.7, C			
2		156.1, C			
2-OH	9.48 (brs)				
3	6.11 (d, 2.2)	100.0, CH	5,6-Me^w	1,2,4,5	4-OH,6-Me^w
4		158.0, C			
4-OH	9.34 (brs)				3^w
5	6.05 (d, 2.2)	108.1, CH	3,6-Me^w	1,3,4,6-Me	4-OH,6-Me
6		137.0, C			
6-Me	2.12 (s)	19.7, CH_3	3^w,5^w	1,2,5,6,7	3,5,7-NH,8^w
7		167.8, C			
7-NH	7.82 (t, 5.6)		8	7,8	6-Me,8,9^w
8	3.12 (dt, 5.6, 6.6)	40.6, CH_2	7-NH, 9	7,9,10	6-Me^w,7-NH,9,10
9	1.48 (tq, 6.6,7.4)	22.4, CH_2	8,10	8,10	7-NH^w,8,10
10	0.88 (t, 7.4)	11.5, CH_3	9	8,9	8,9

[a] Spectra recorded at 25 °C (500 MHz for ^{1}H NMR and 125 MHz for ^{13}C NMR); [w] Weak correlation.

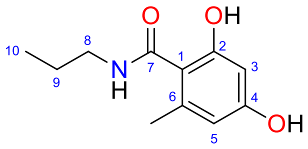

High resolution mass spectrum of compound **7**

Mass Spectrum SmartFormula Report

Analysis Info

Analysis Name	D:\Data\Sasha\20231004\RAD915D000001.d	Acquisition Date	10/4/2023 2:05:35 PM
Method	DirectInfusion_2018_pos.m	Operator	Demo User
Sample Name	RAD915D	Instrument	maXis II ETD
Comment			

Acquisition Parameter

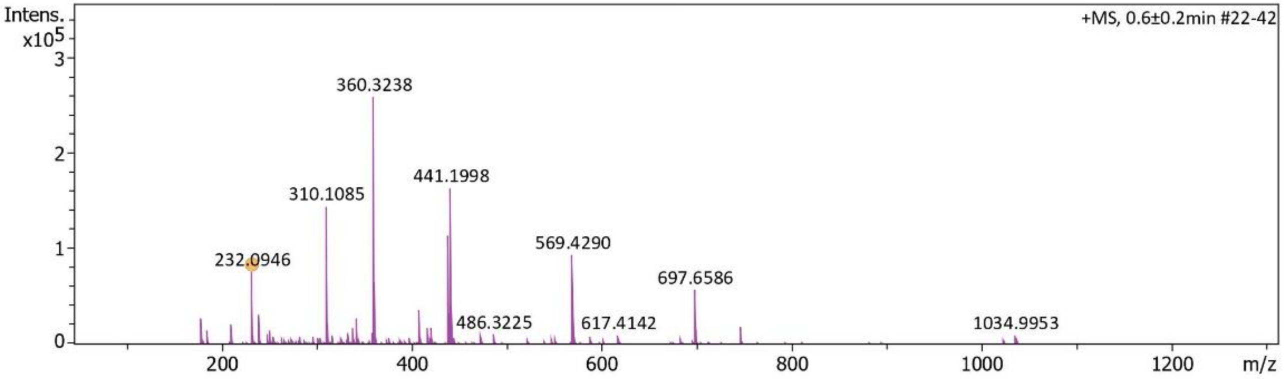

Meas. m/z	#	Ion Formula	m/z	err [ppm]	mSigma	# mSigma	Score	rdb	e⁻ Conf	N-Rule
232.0946	1	C11H15NNaO3	232.0944	-0.7	7.4	1	100.00	5.0	even	ok

^{1}H NMR spectrum of compound **8** in DMSO-d_6

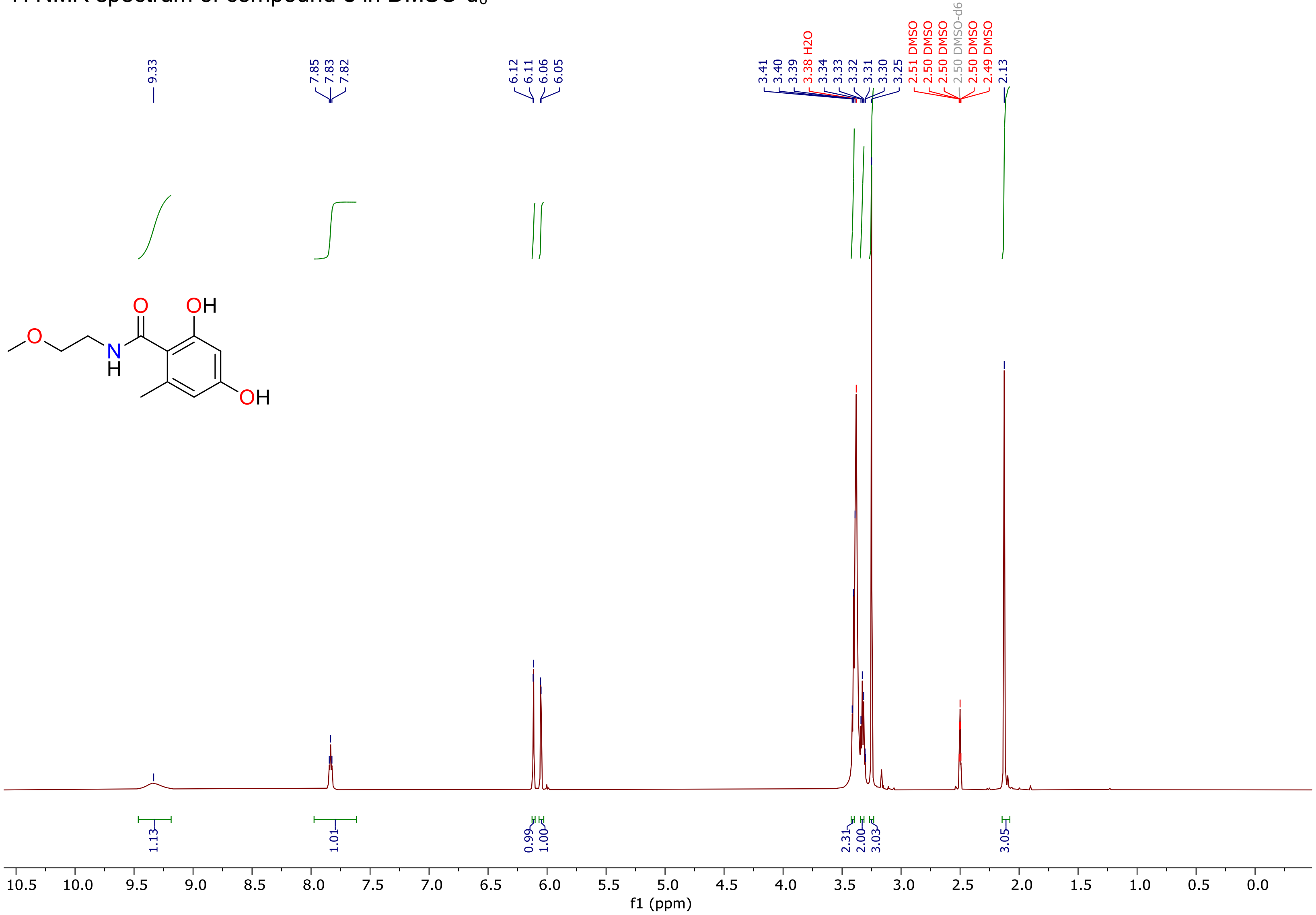

^{13}C NMR spectrum of compound **8** in DMSO-d_6

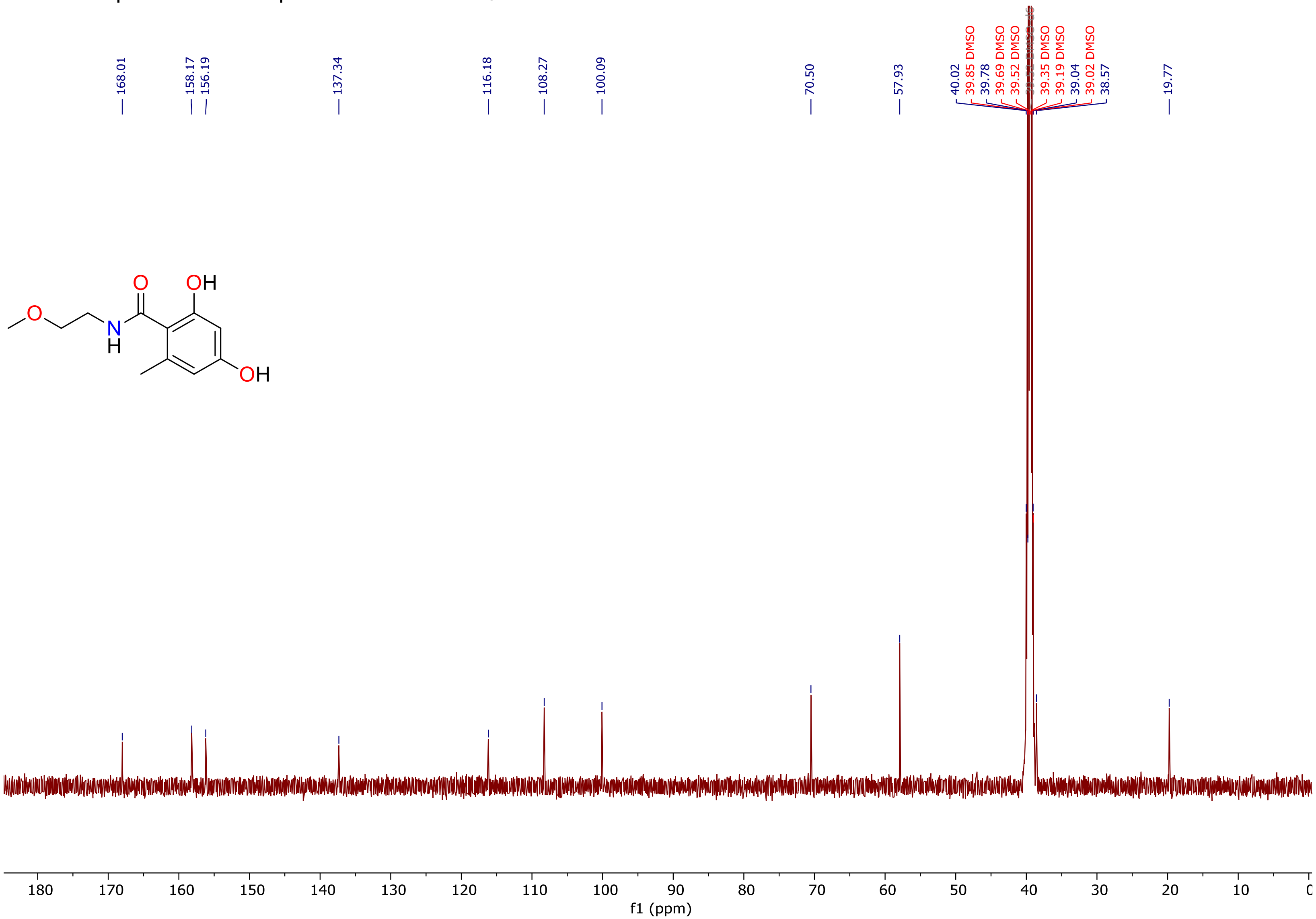

HSQC spectrum of compound **8** in DMSO-d_6

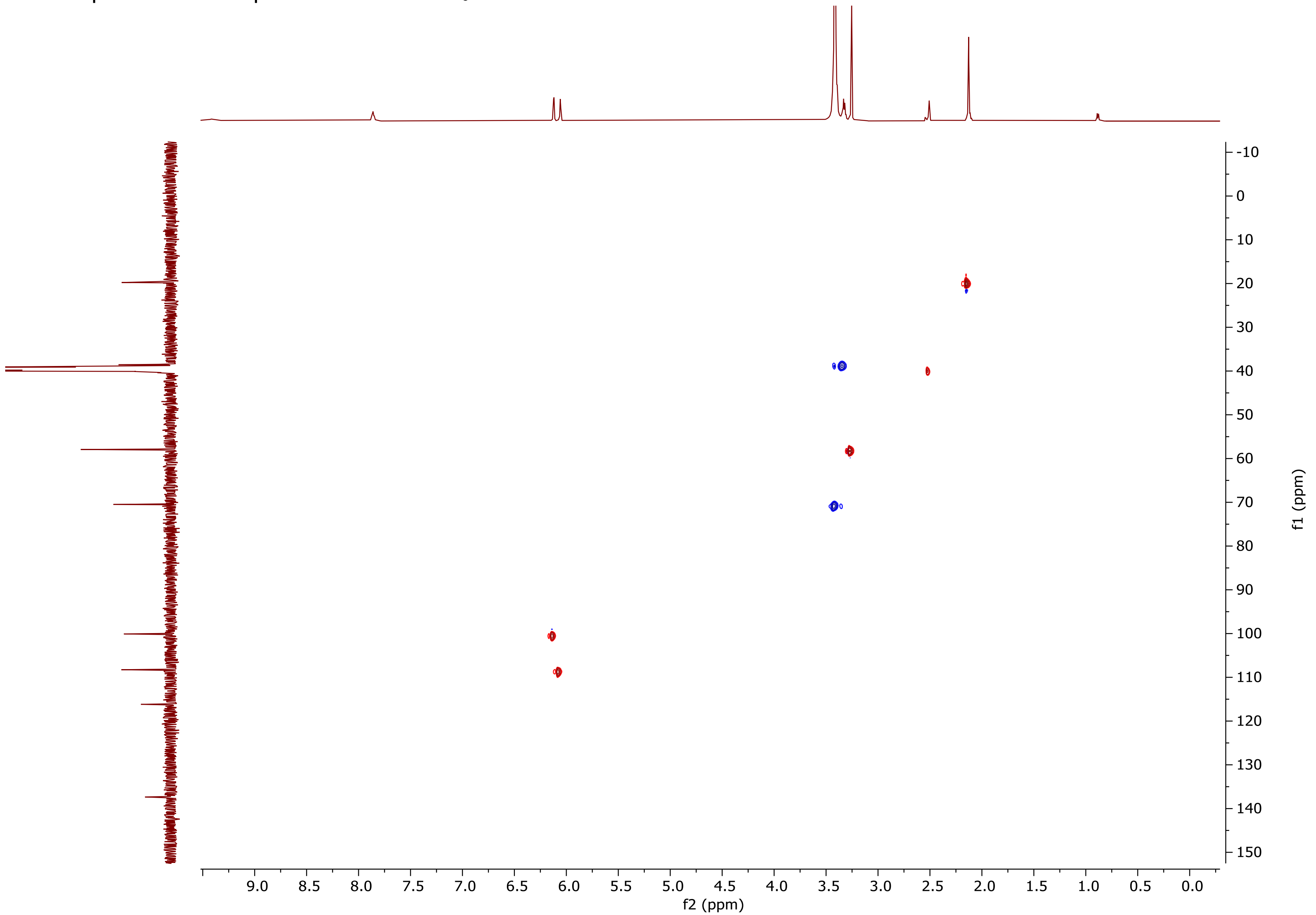

HMBC spectrum of compound **8** in DMSO-d_6

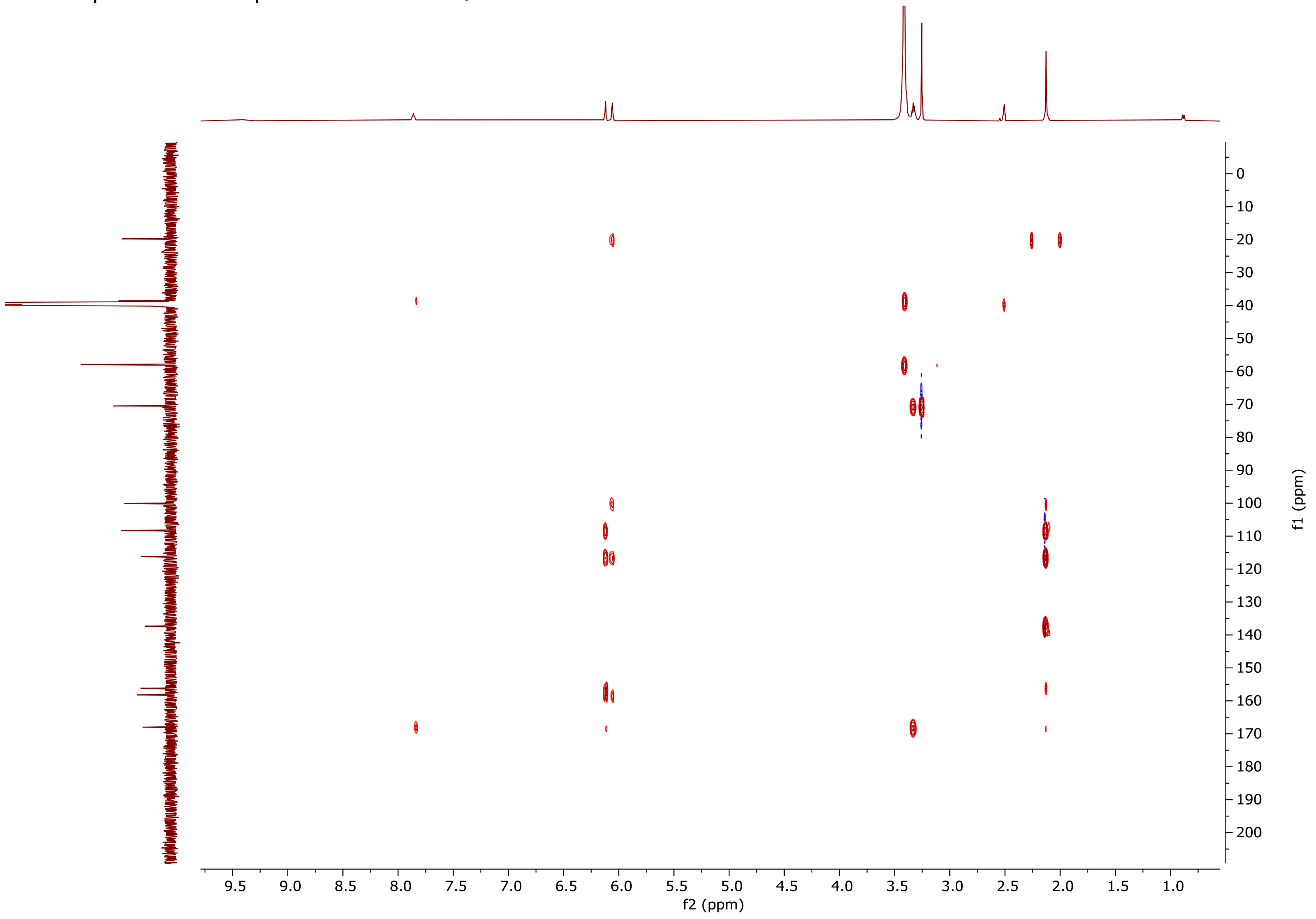

COSY spectrum of compound **8** in DMSO-d_6

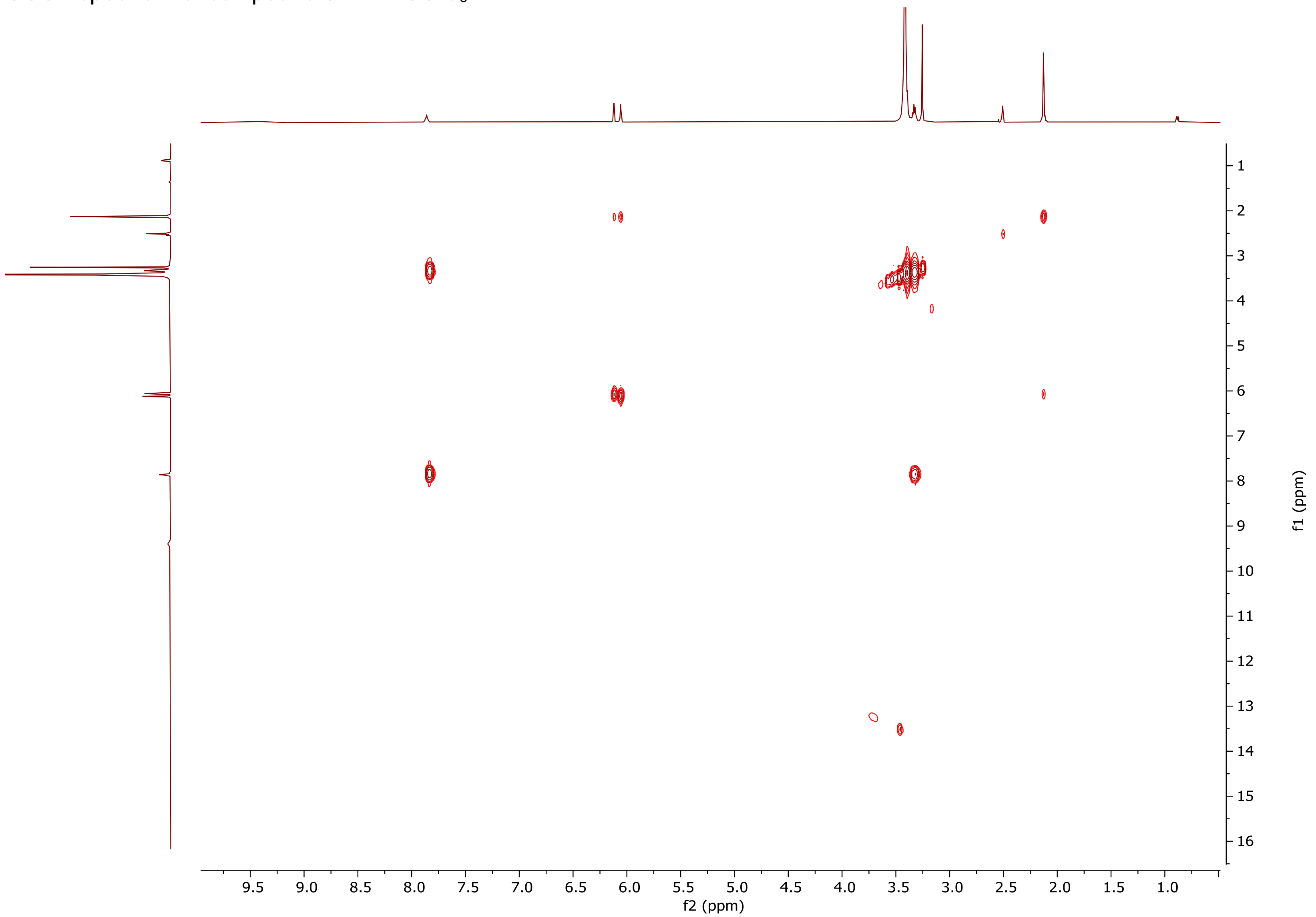

ROESY spectrum of compound **8** in DMSO-d_6

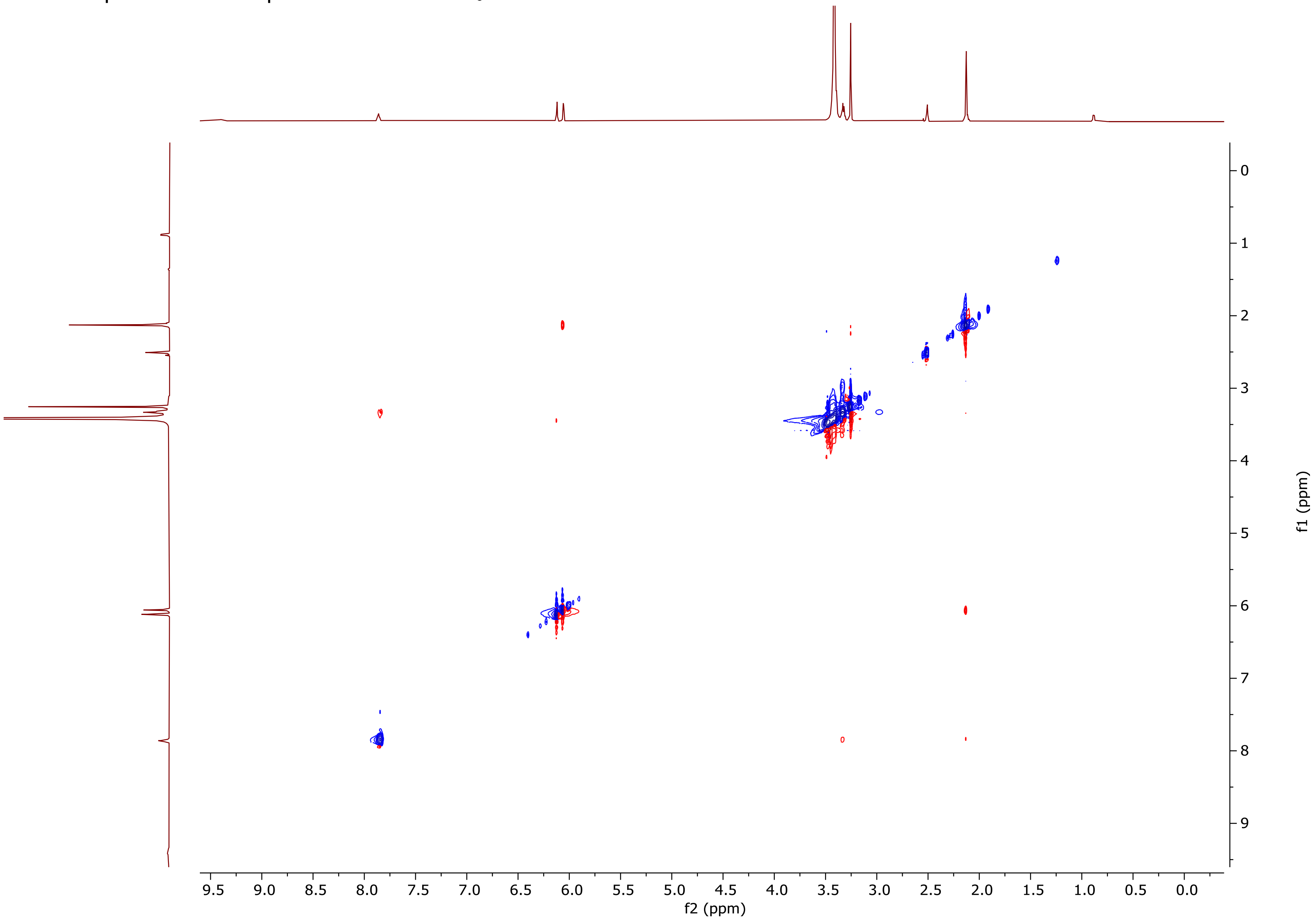

NMR data table for compound **8** in DMSO-d_6[a]

Position	δ_H (mult., *J* in Hz)	δ_C, type	COSY	HMBC	ROESY
1		116.2, C			
2		156.2, C			
2-OH	[b]				
3	6.11 (d, 2.2)	100.1, CH	5,6-Me[w]	1,4,5	6-Me
4		158.2, C			
4-OH	9.33 (brs)				
5	6.05 (d, 2.2)	108.3, CH	3,6-Me[w]	1,3,4,6-Me	6-Me
6		137.3, C			
6-Me	2.13 (s)	19.8, CH_3	3[w],5[w]	1,2,5,6,7[w]	5,7-NH[w]
7		168.0, C			
7-NH	7.83 (t, 5.7)		8	7,8	6-Me[w],8,9
8	3.33 (dt, 5.7, 5.8)	38.6, CH_2	7-NH, 9	7,9	7-NH,9,10
9	3.40 (t, 5.8)	70.5, CH_2	8	8,10	7-NH[w],8,10
10	3.25 (s)	57.9, CH_3		9	8,9

[a] Spectra recorded at 25 °C (500 MHz for ^1H NMR and 125 MHz for ^{13}C NMR); [b] Not observed; [w] Weak correlation.

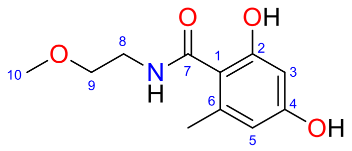

High resolution mass spectrum of compound **8**

Mass Spectrum SmartFormula Report

Analysis Info

Analysis Name	D:\Data\Sasha\20231004\RAD915A000001.d	Acquisition Date	10/4/2023 1:48:42 PM
Method	DirectInfusion_2018_pos.m	Operator	Demo User
Sample Name	RAD915A	Instrument	maXis II ETD
Comment			

Acquisition Parameter

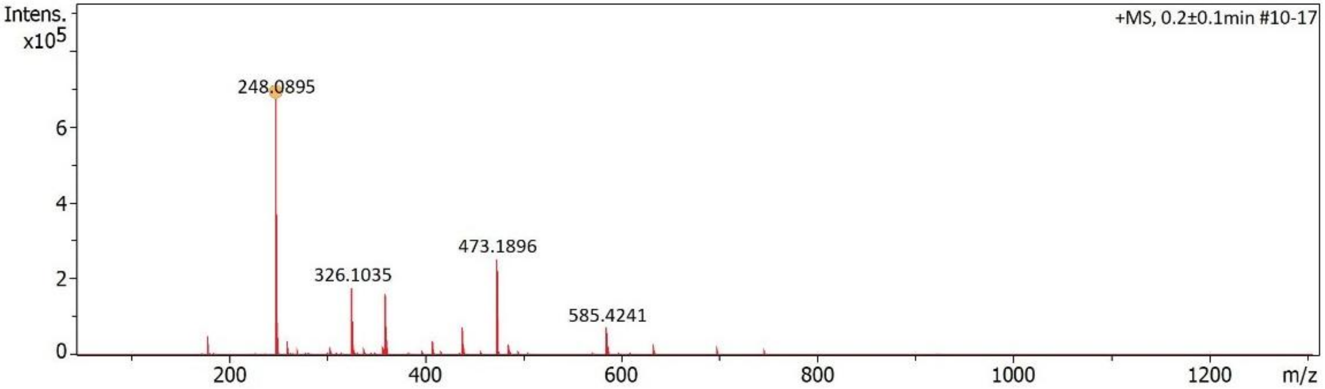

Meas. m/z	#	Ion Formula	m/z	err [ppm]	mSigma	# mSigma	Score	rdb	e⁻ Conf	N-Rule
248.0895	1	C11H15NNaO4	248.0893	-0.7	0.3	1	100.00	5.0	even	ok
	2	C12H11N5Na	248.0907	4.7	13.6	2	47.59	10.0	even	ok
	3	C7H11N7NaO2	248.0866	-11.5	13.7	3	12.91	6.0	even	ok

^{1}H NMR spectrum of compound **9** in DMSO-d_6

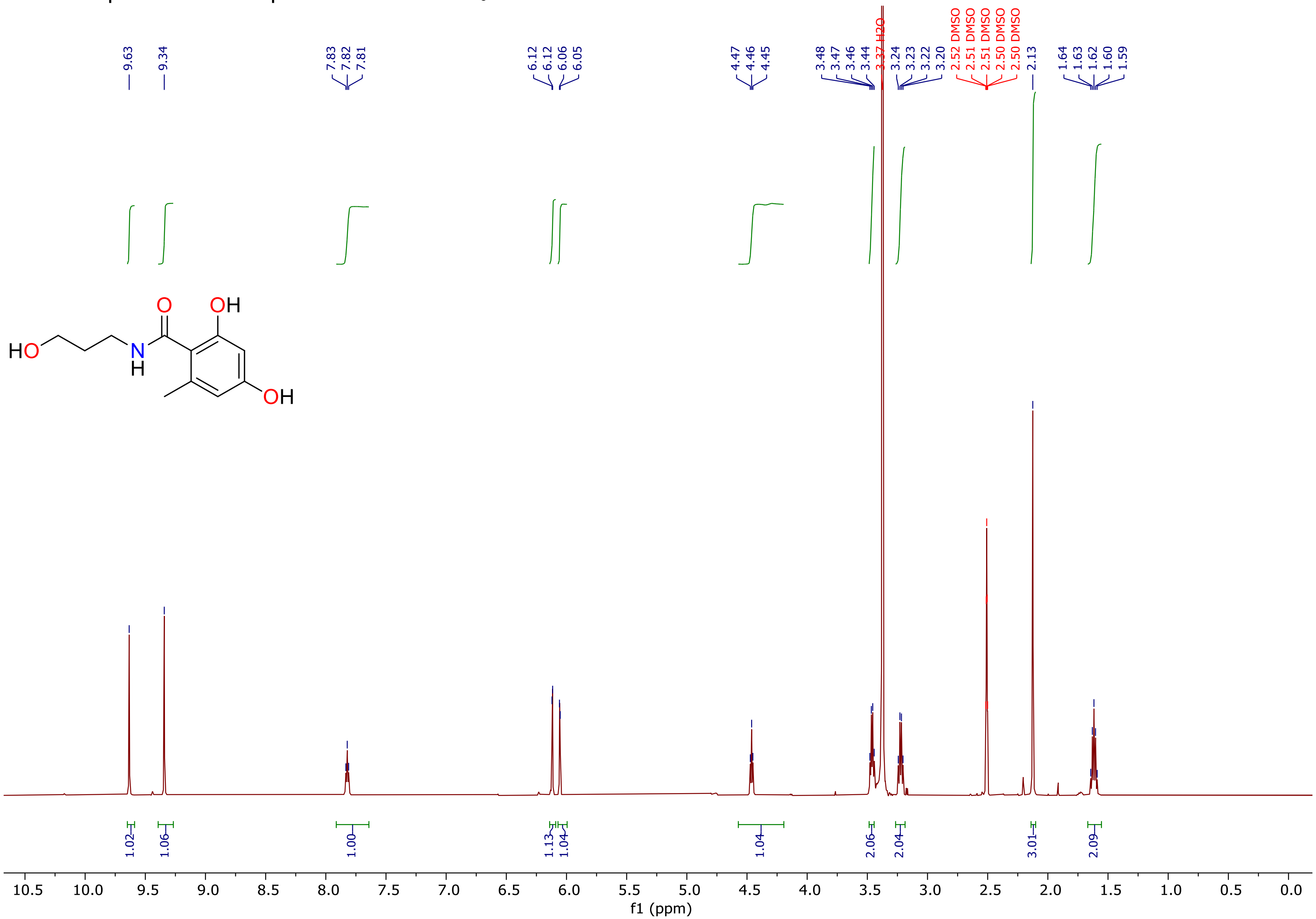

^{13}C NMR spectrum of compound **9** in DMSO-d_6

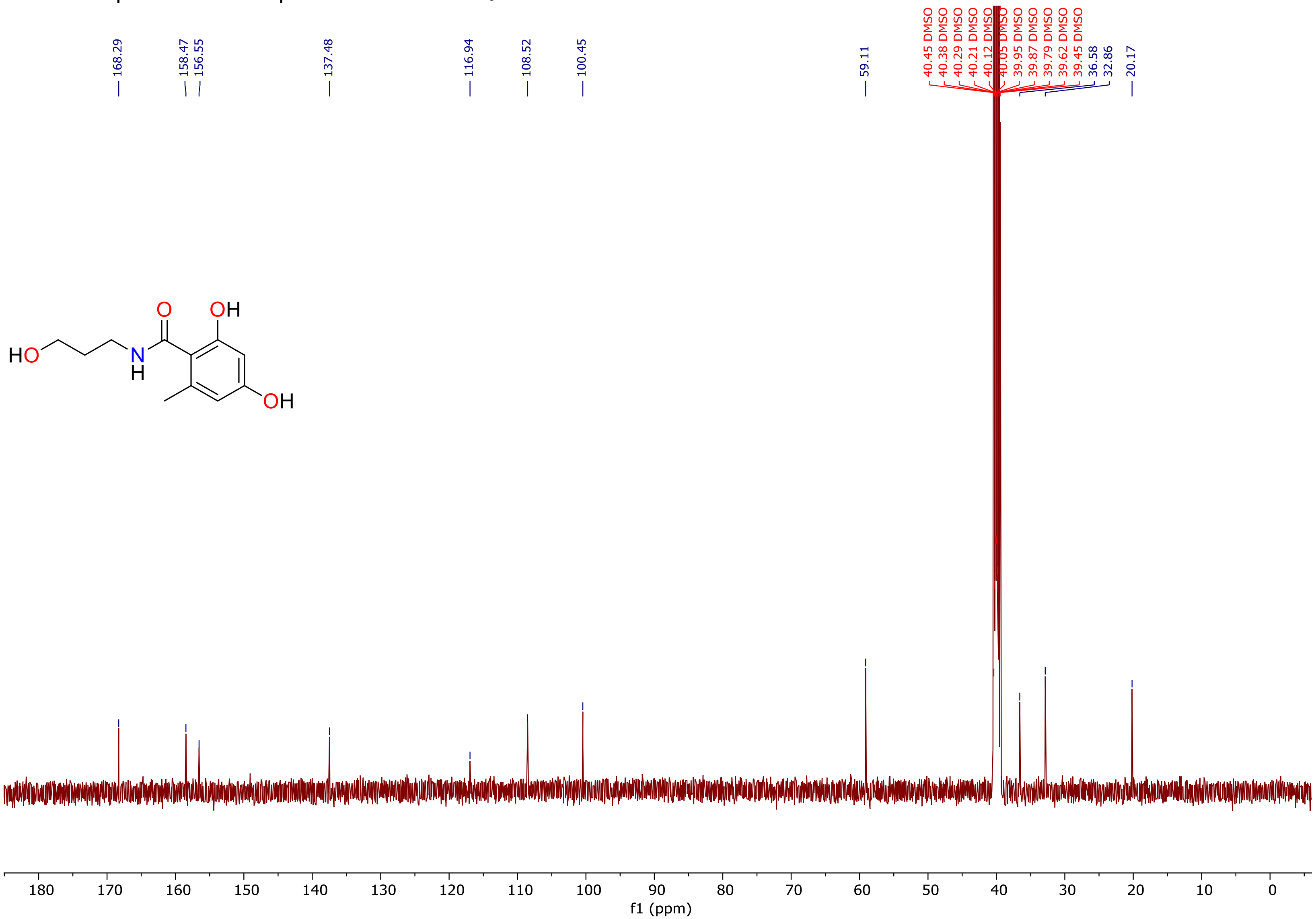

HSQC spectrum of compound **9** in DMSO-d_6

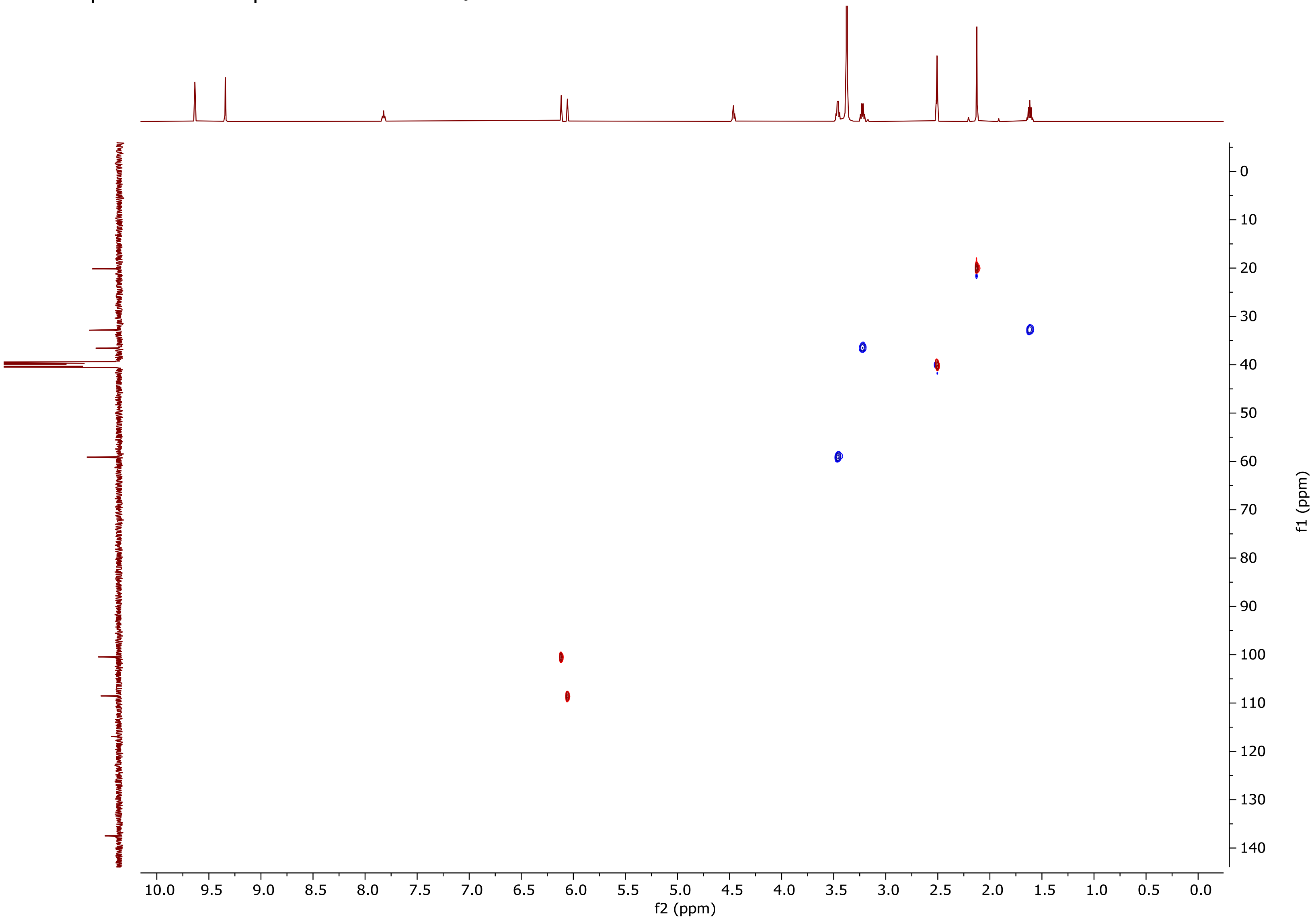

HMBC spectrum of compound **9** in DMSO-d_6

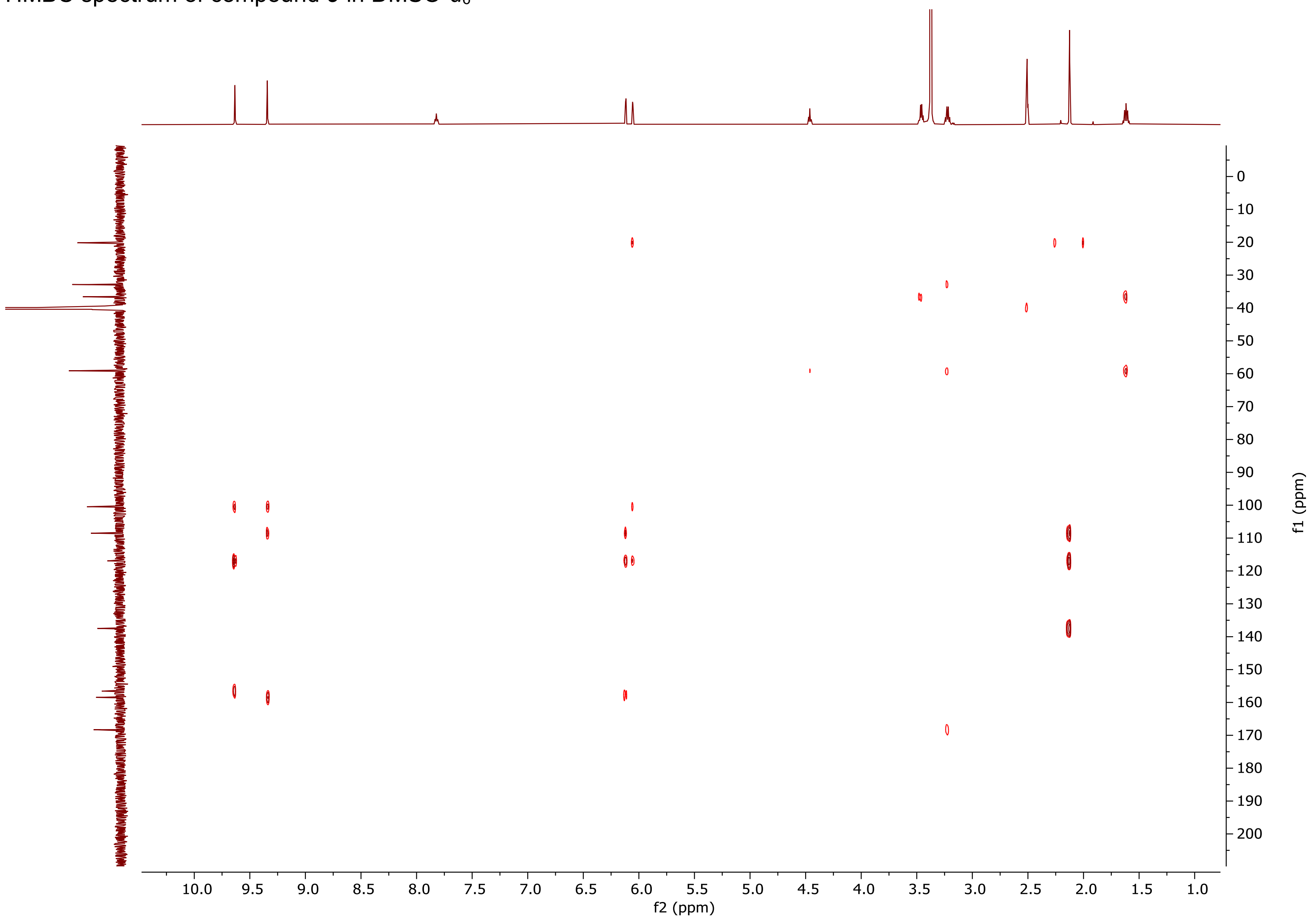

COSY spectrum of compound **9** in DMSO-d_6

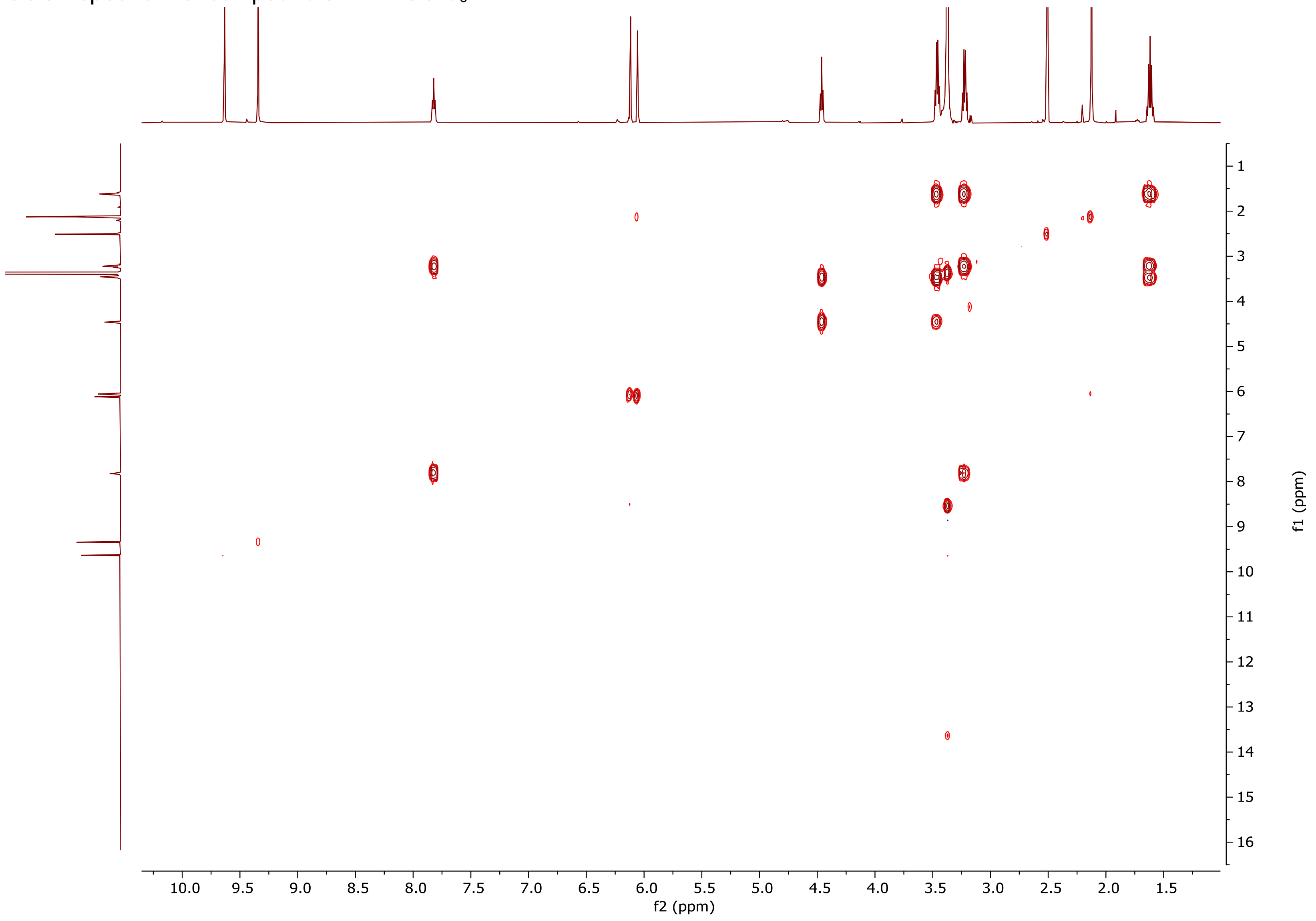

ROESY spectrum of compound **9** in DMSO-d_6

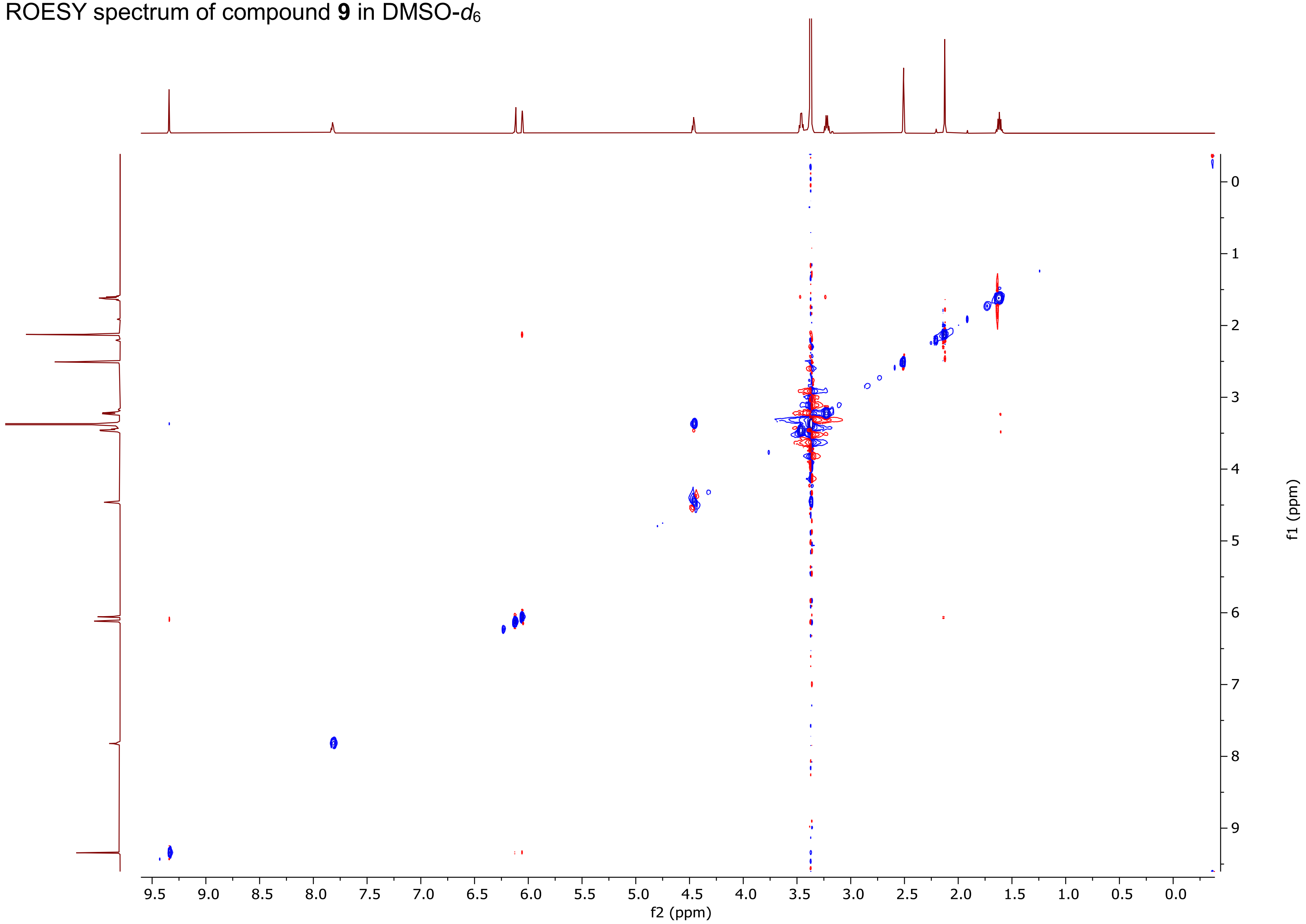

NMR data table for compound **9** in DMSO-d_6[a]

Position	δ_H (mult., *J* in Hz)	δ_C, type	COSY	HMBC	ROESY
1		116.9, C			
2		156.6, C			
2-OH	9.63 (brs)			1,2,3	3
3	6.12 (d, 2.3)	100.5, CH	5^w,6-Me^w	1,2,4,5,7^w	2-OH,4-OH
4		158.5, C			
4-OH	9.34 (brs)			3,4,5	3,5
5	6.05 (d, 2.3)	108.5, CH	3,6-Me^w	1,3,4,6-Me	4-OH,6-Me
6		137.5, C			
6-Me	2.13 (s)	20.2, CH_3	3^w,5^w	1,2^w,3^w,4^w,5,6	5,7-NH
7		168.3, C			
7-NH	7.82 (t, 5.6)		8	7^w	6-Me,8
8	3.22 (dt, 5.6, 6.6)	36.6, CH_2	7-NH,9,10	9,10	7-NH,9^w
9	1.62 (tq, 6.6, 6.2)	32.9, CH_2	8,10	8,10	8,10
10	3.46 (dt, 5.5, 6.2)	59.1, CH_2	9,10-OH	8,9	9^w,10-OH
10-OH	4.46 (t, 5.4)		10	9^w,10	10

[a] Spectra recorded at 25 °C (500 MHz for ^1H NMR and 125 MHz for ^{13}C NMR); [w] Weak correlation.

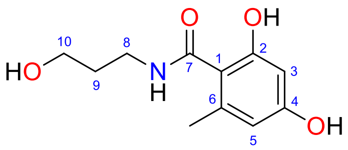

High resolution mass spectrum of compound **9**

Mass Spectrum SmartFormula Report

Analysis Info

Analysis Name	D:\Data\Sasha\20231004\RAD915H000001.d	Acquisition Date	10/4/2023 2:23:44 PM
Method	DirectInfusion_2018_pos.m	Operator	Demo User
Sample Name	RAD915H	Instrument	maXis II ETD
Comment			

Acquisition Parameter

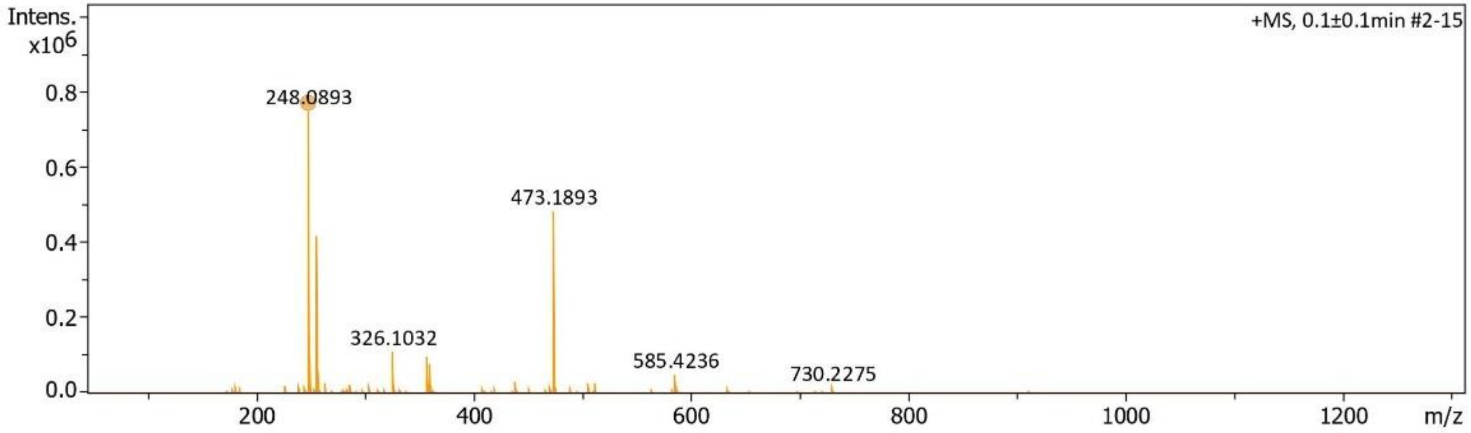

Meas. m/z	#	Ion Formula	m/z	err [ppm]	mSigma	# mSigma	Score	rdb	e⁻ Conf	N-Rule
248.0893	1	C11H15NNaO4	248.0893	-0.0	0.5	1	100.00	5.0	even	ok

^{1}H NMR spectrum of compound **10** in DMSO-d_6

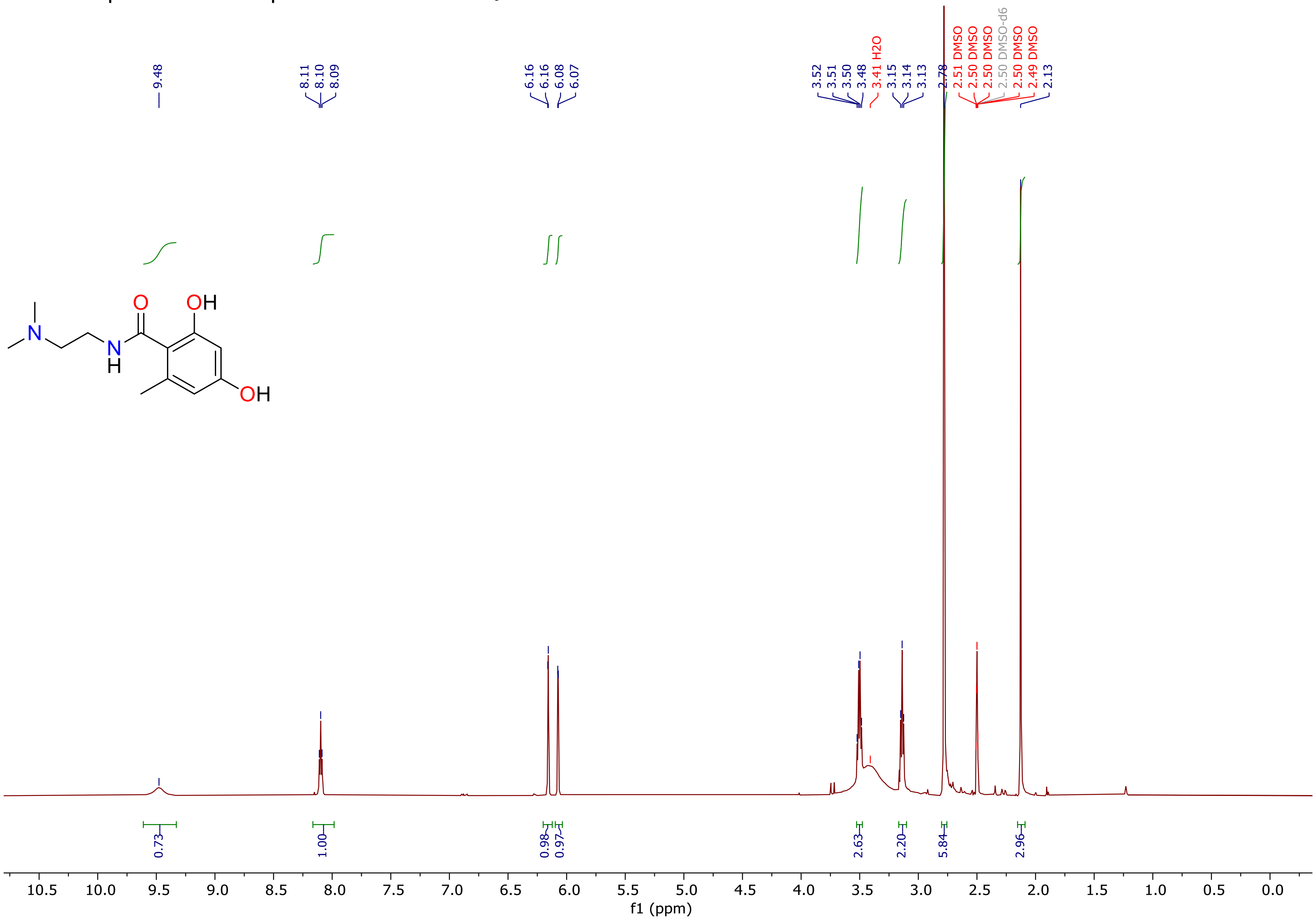

^{13}C NMR spectrum of compound **10** in DMSO-d_6

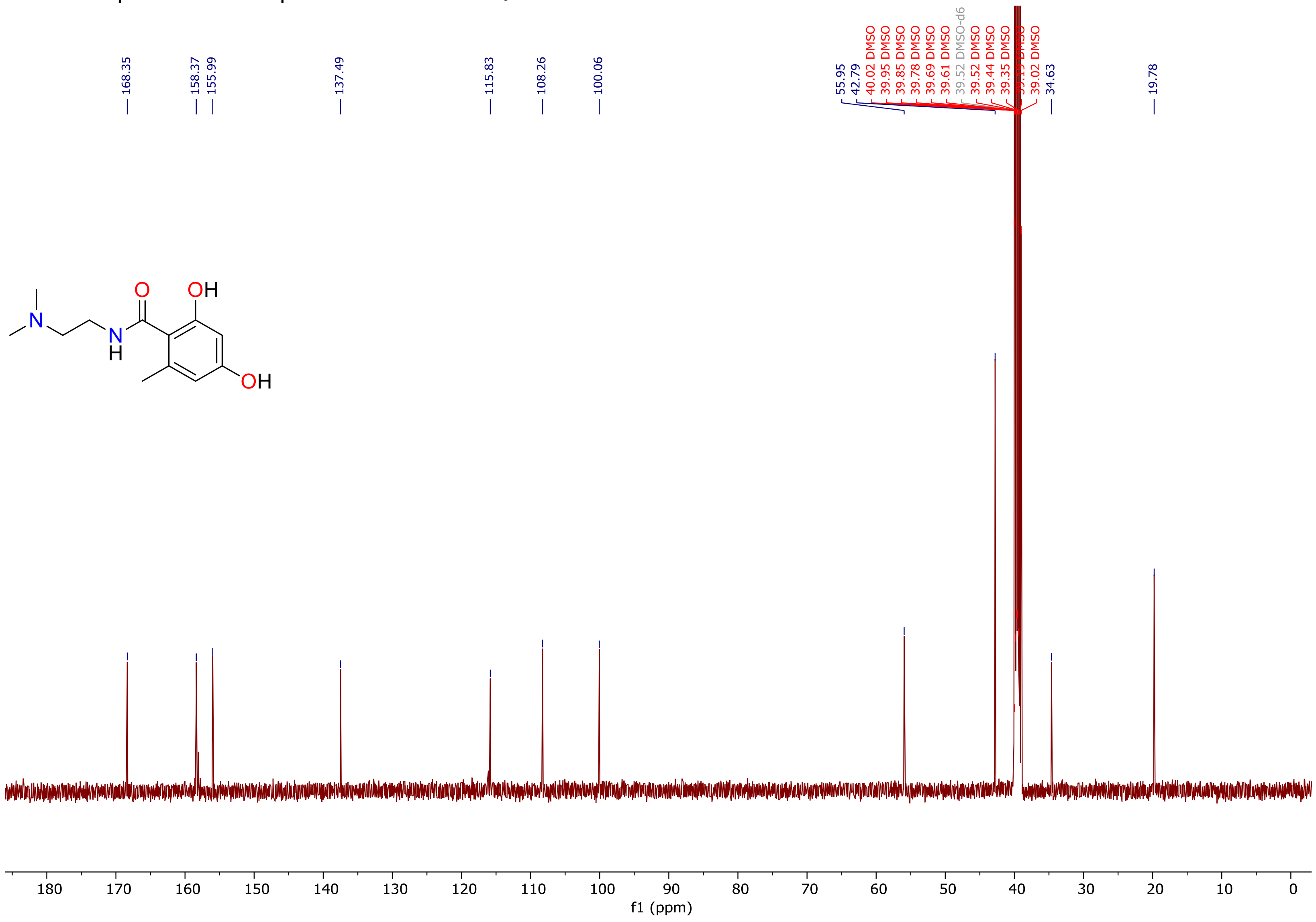

HSQC spectrum of compound **10** in DMSO-d_6

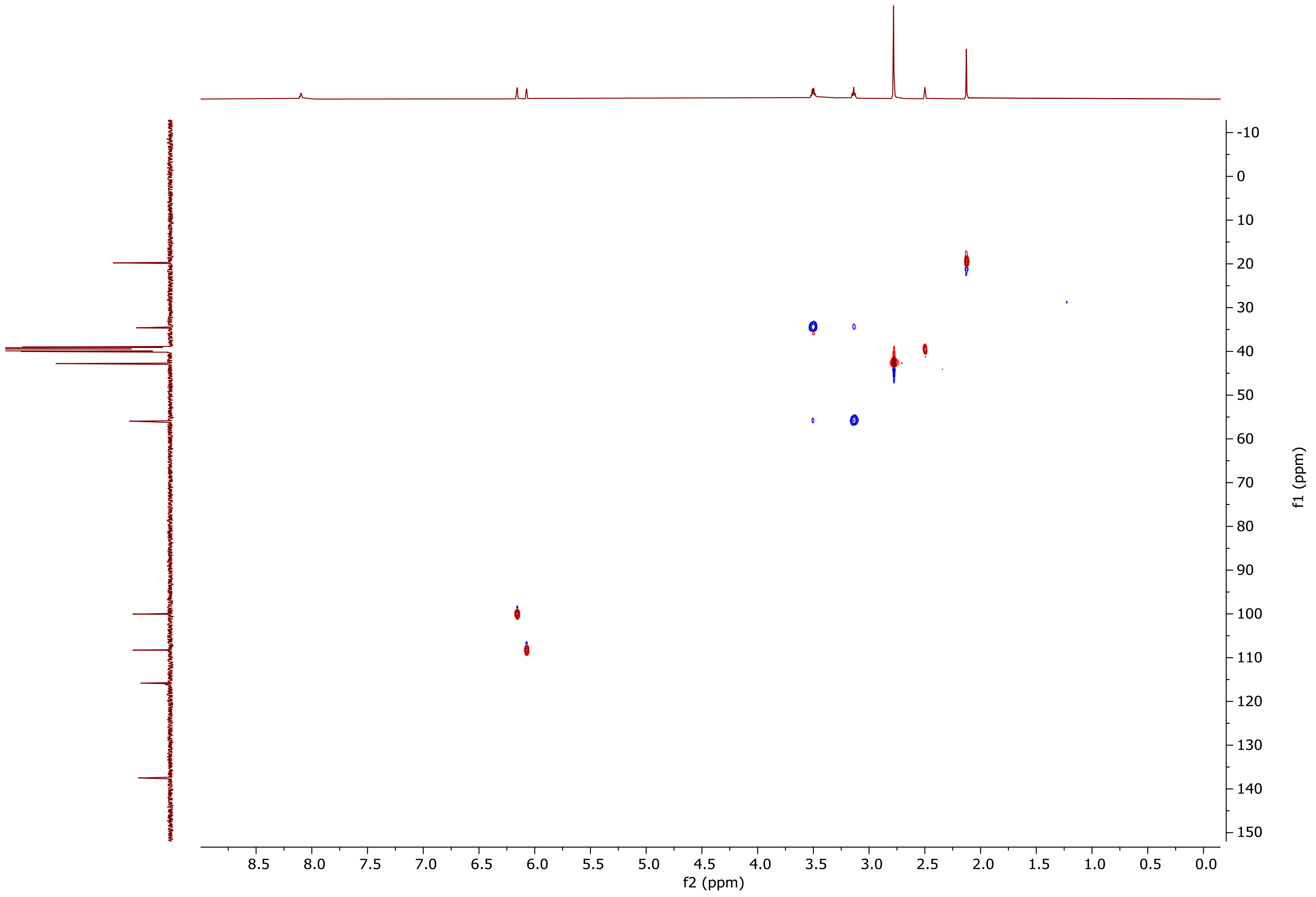

HMBC spectrum of compound **10** in DMSO-d_6

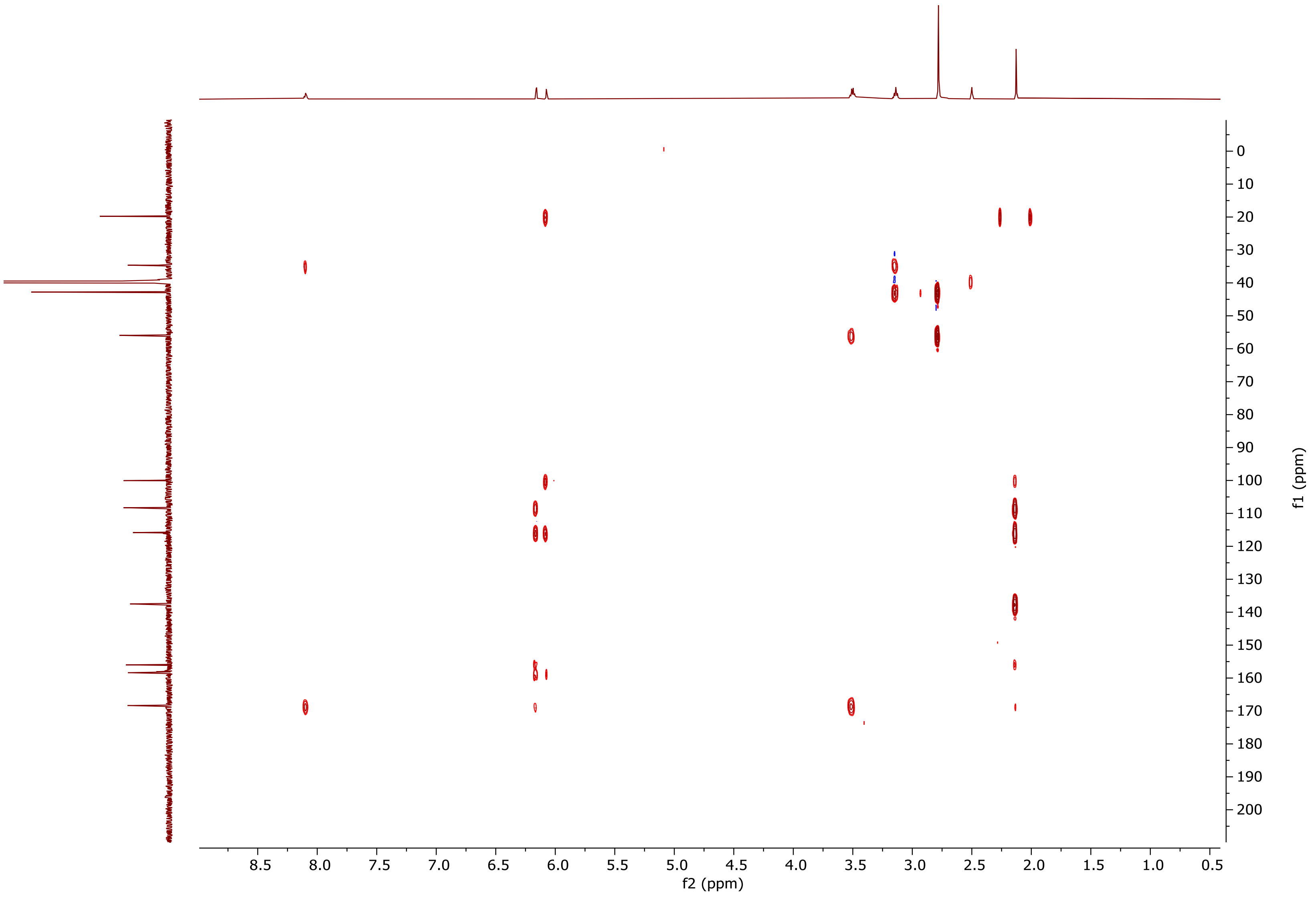

COSY spectrum of compound **10** in DMSO-d_6

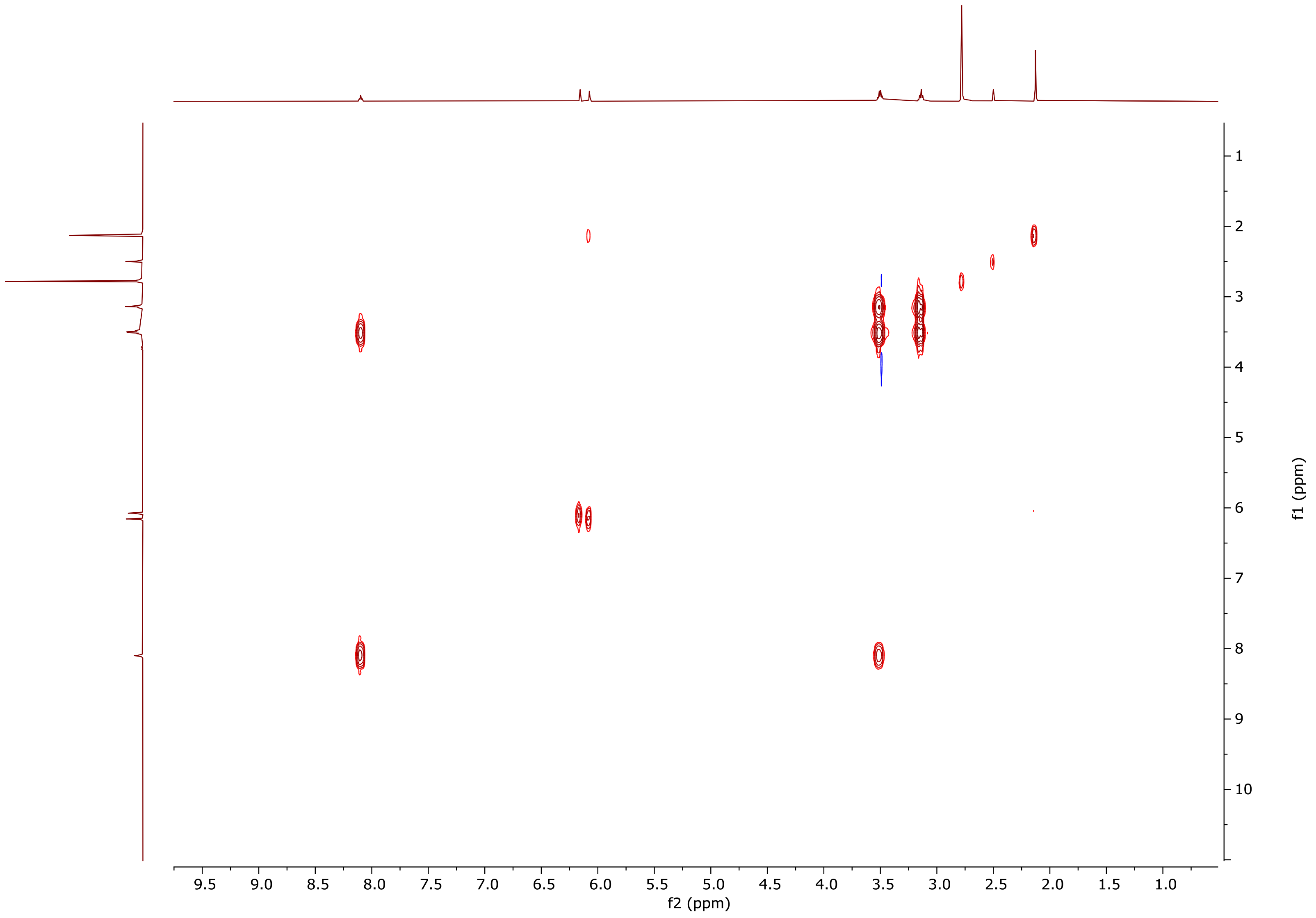

ROESY spectrum of compound **10** in DMSO-d_6

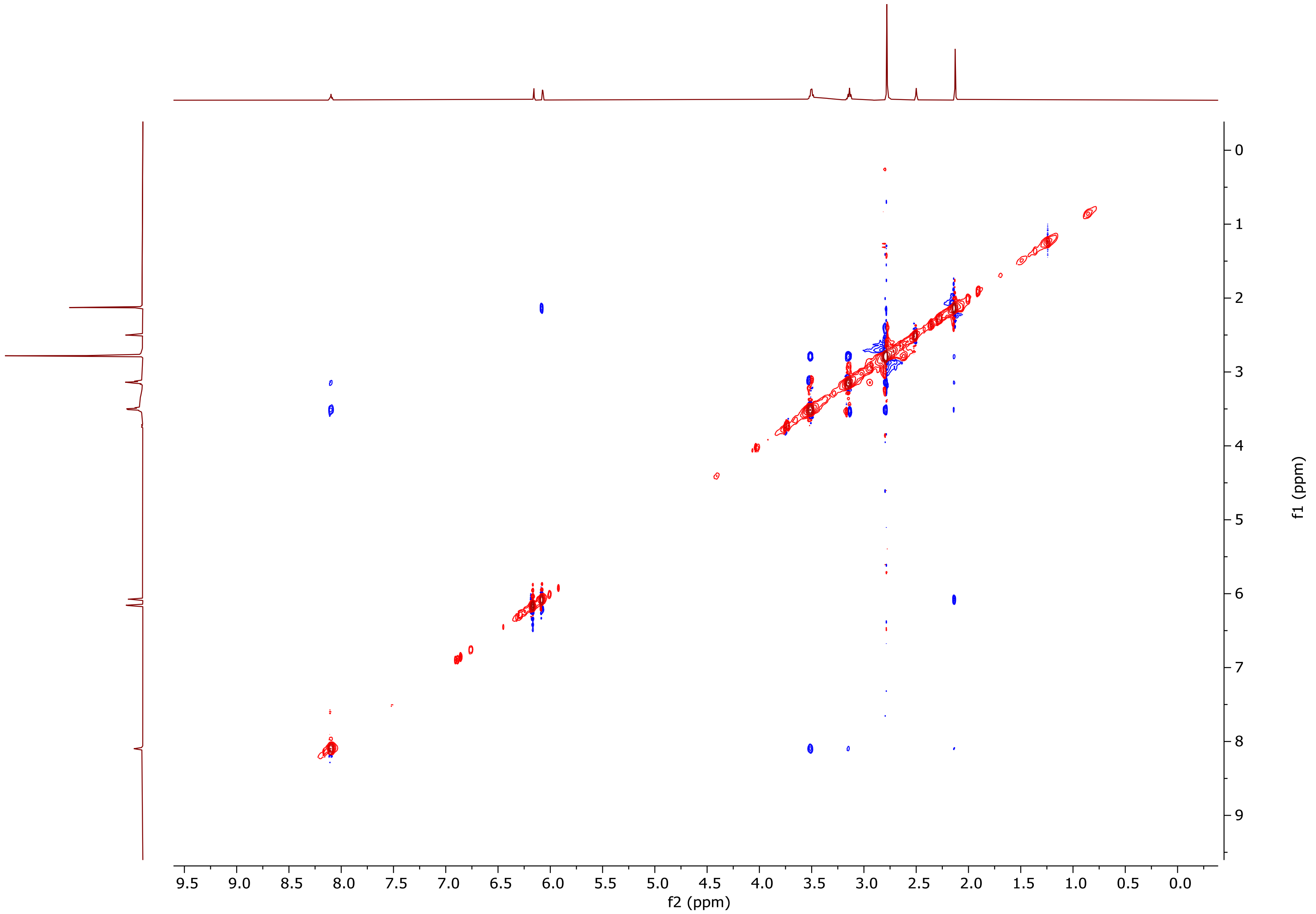

NMR data table for compound **10** in DMSO-d_6[a, b]

Position	δ_H (mult., *J* in Hz)	δ_C, type	COSY	HMBC	ROESY
1		115.8, C			
2		156.0, C			
2-OH	[c]				
3	6.16 (d, 2.0)	100.1, CH	5,6-Me[w]	1,2,4,5,7[w]	
4		158.4, C			
4-OH	9.48 (brs)				
5	6.08 (d, 2.0)	108.3, CH	3,6-Me[w]	1,3,4,6-Me	6-Me
6		137.5, C			
6-Me	2.13 (s)	19.8, CH_3	3[w],5[w]	1,2[w],3[w],4,5,6,7[w]	5,7-NH,8,9
7		168.4, C			
7-NH	8.10 (t, 5.6)		8	7,8	6-Me[w],8,9[w],10[w],11[w]
8	3.50 (dt, 5.6, 6.4)	34.6, CH_2	7-NH,9	7,9	6-Me[w],7-NH,9,10,11
9	3.14 (t, 6.4)	56.0, CH_2	8	8,10,11	6-Me[w],7-NH[w],8,10,11
9-NH	[b,c]				
10	2.78 (s)	42.8, CH_3		9,11	8,9
11	2.78 (s)	42.8, CH_3		9,10	8,9

[a] Spectra recorded at 25 °C (500 MHz for ^{1}H NMR and 125 MHz for ^{13}C NMR); [b] Purified as a TFA salt; [c] Not observed; [w] Weak correlation.

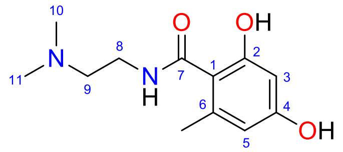

High resolution mass spectrum of compound **10**

Mass Spectrum SmartFormula Report

Analysis Info

Analysis Name	D:\Data\Sasha\20231004\RAD915E000001.d	Acquisition Date	10/4/2023 2:12:02 PM
Method	DirectInfusion_2018_pos.m	Operator	Demo User
Sample Name	RAD915E	Instrument	maXis II ETD
Comment			

Acquisition Parameter

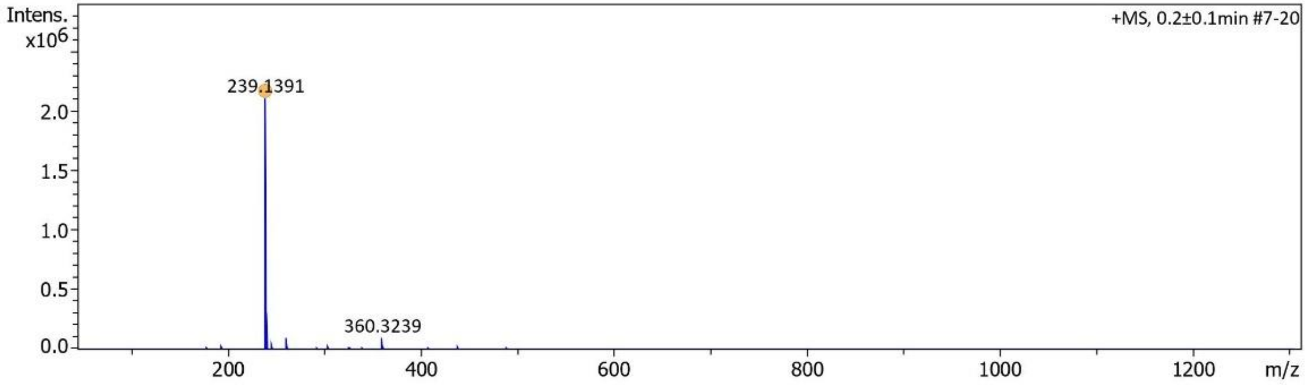

Meas. m/z	#	Ion Formula	m/z	err [ppm]	mSigma	# mSigma	Score	rdb	e⁻ Conf	N-Rule
239.1391	1	C12H19N2O3	239.1390	-0.4	3.4	1	100.00	5.0	even	ok

^{1}H NMR spectrum of compound **11** in DMSO-d_6

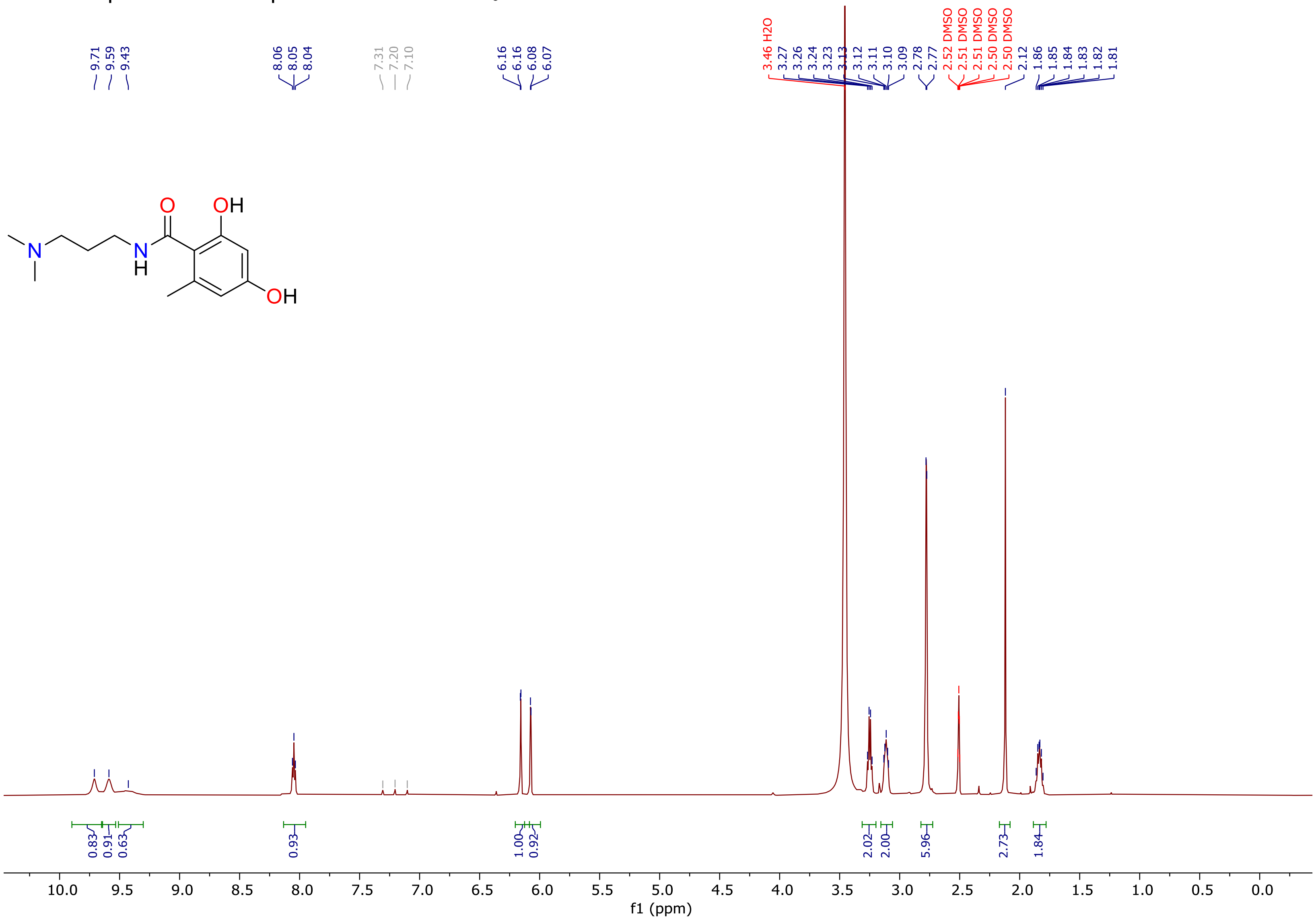

^{13}C NMR spectrum of compound **11** in DMSO-d_6

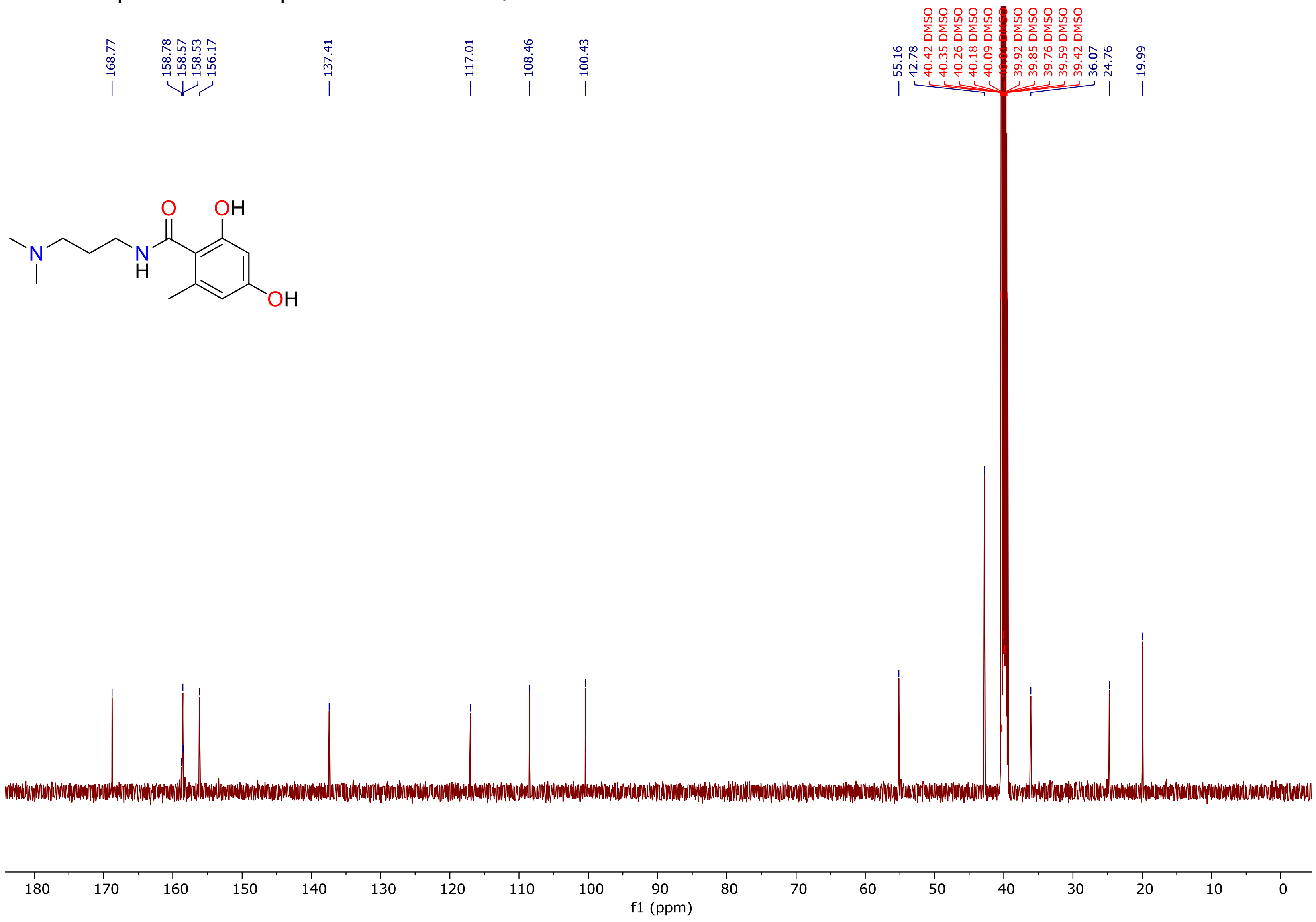

HSQC spectrum of compound **11** in DMSO-d_6

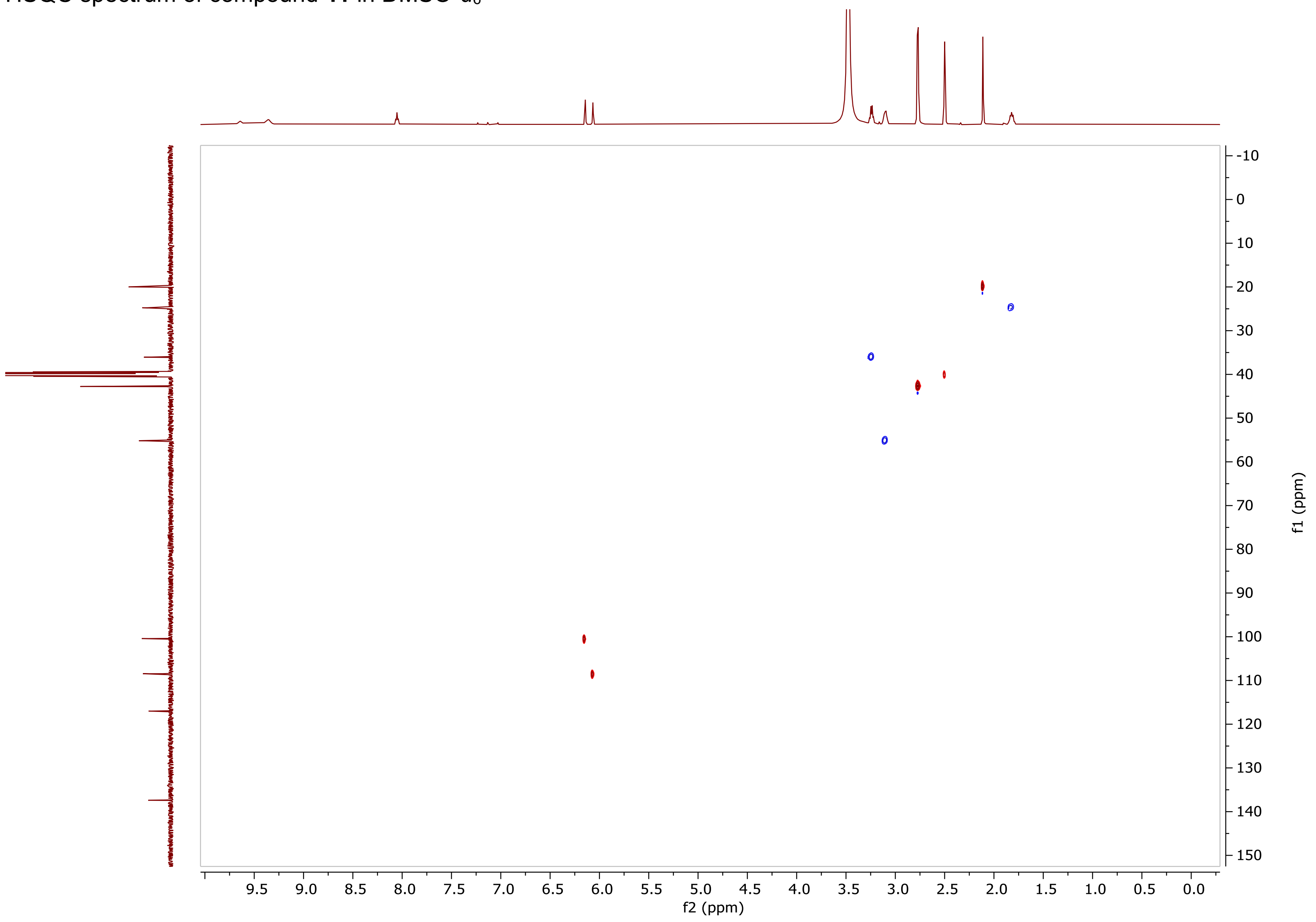

HMBC spectrum of compound **11** in DMSO-d_6

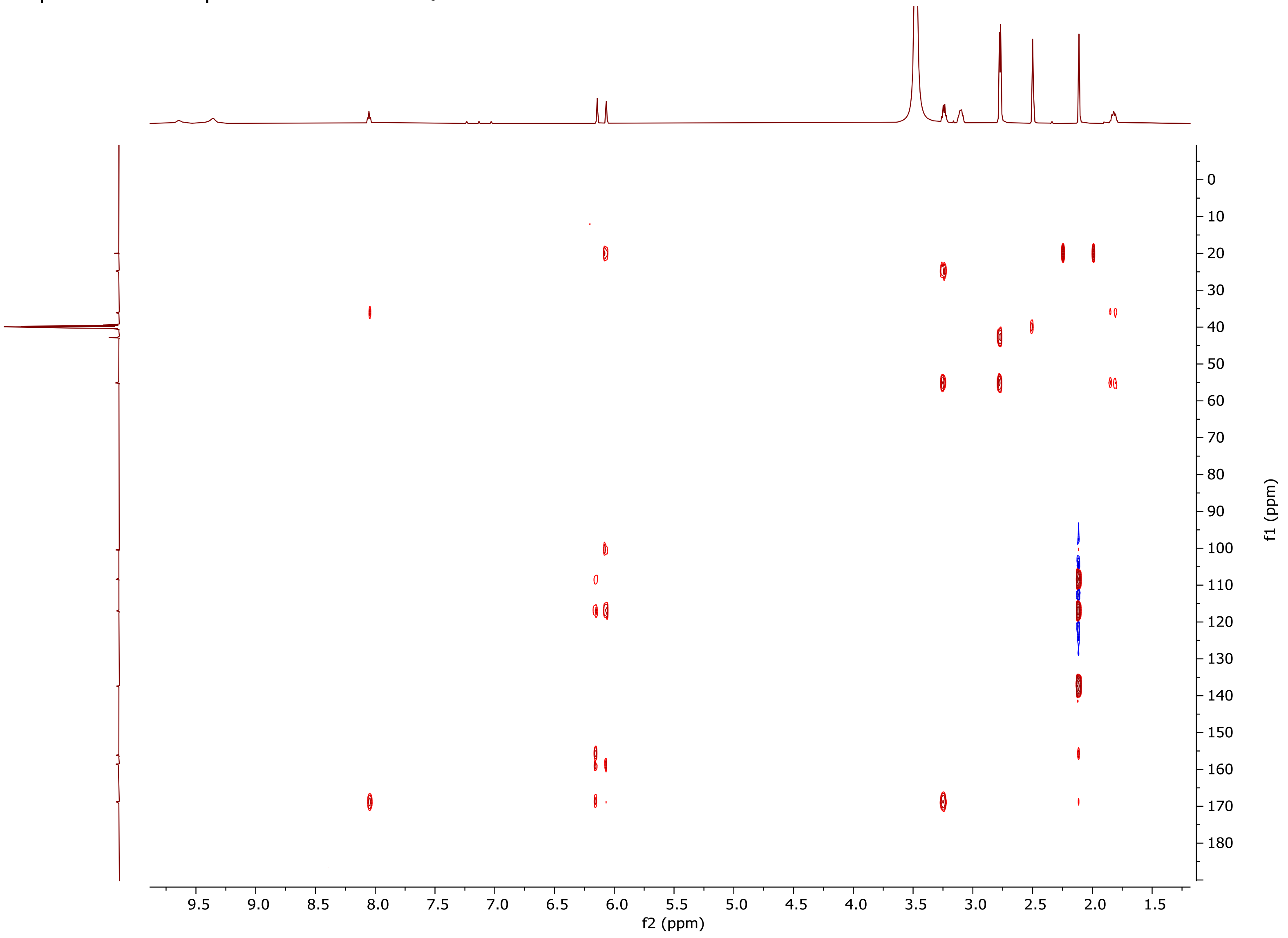

COSY spectrum of compound **11** in DMSO-d_6

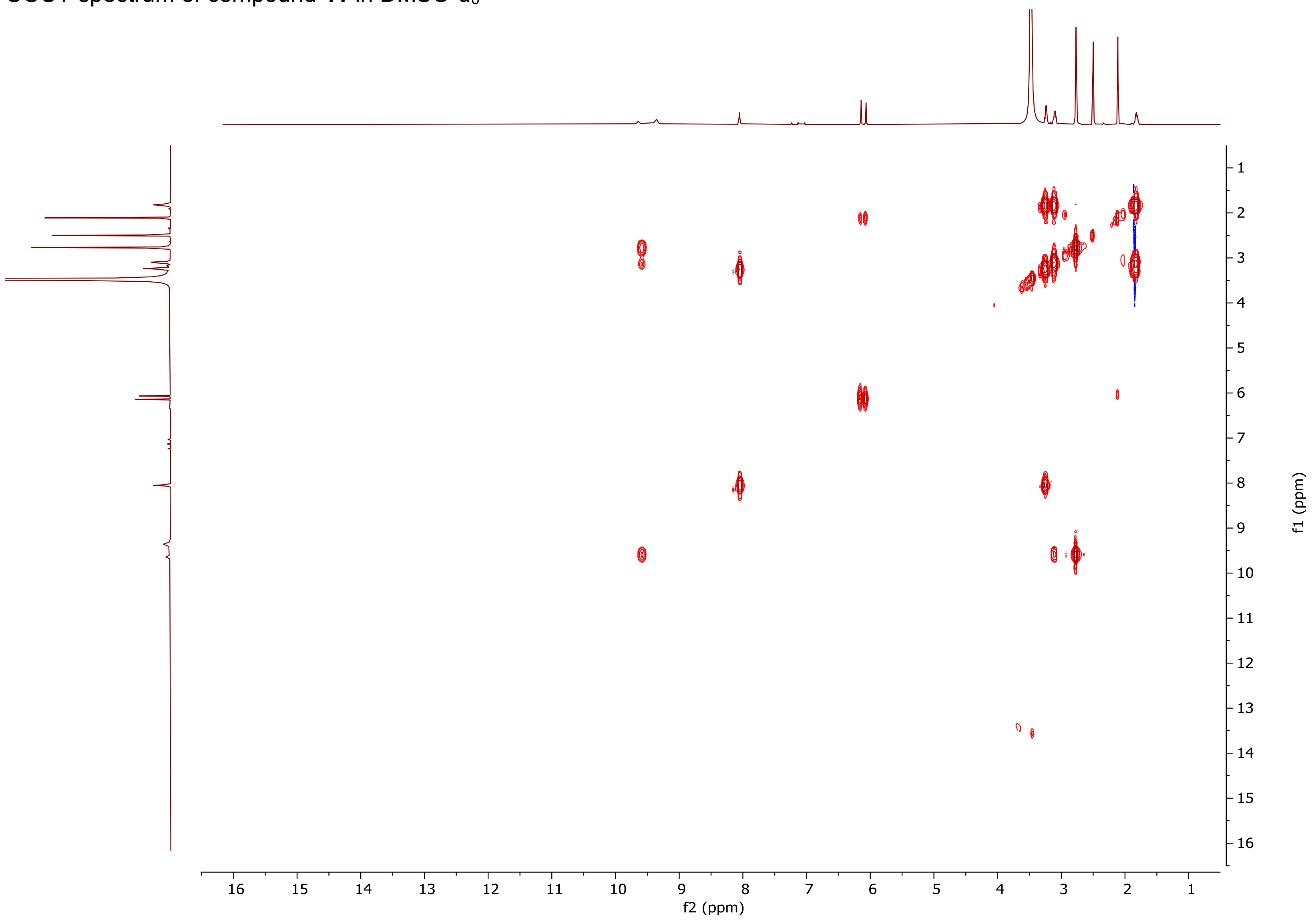

ROESY spectrum of compound **11** in DMSO-d_6

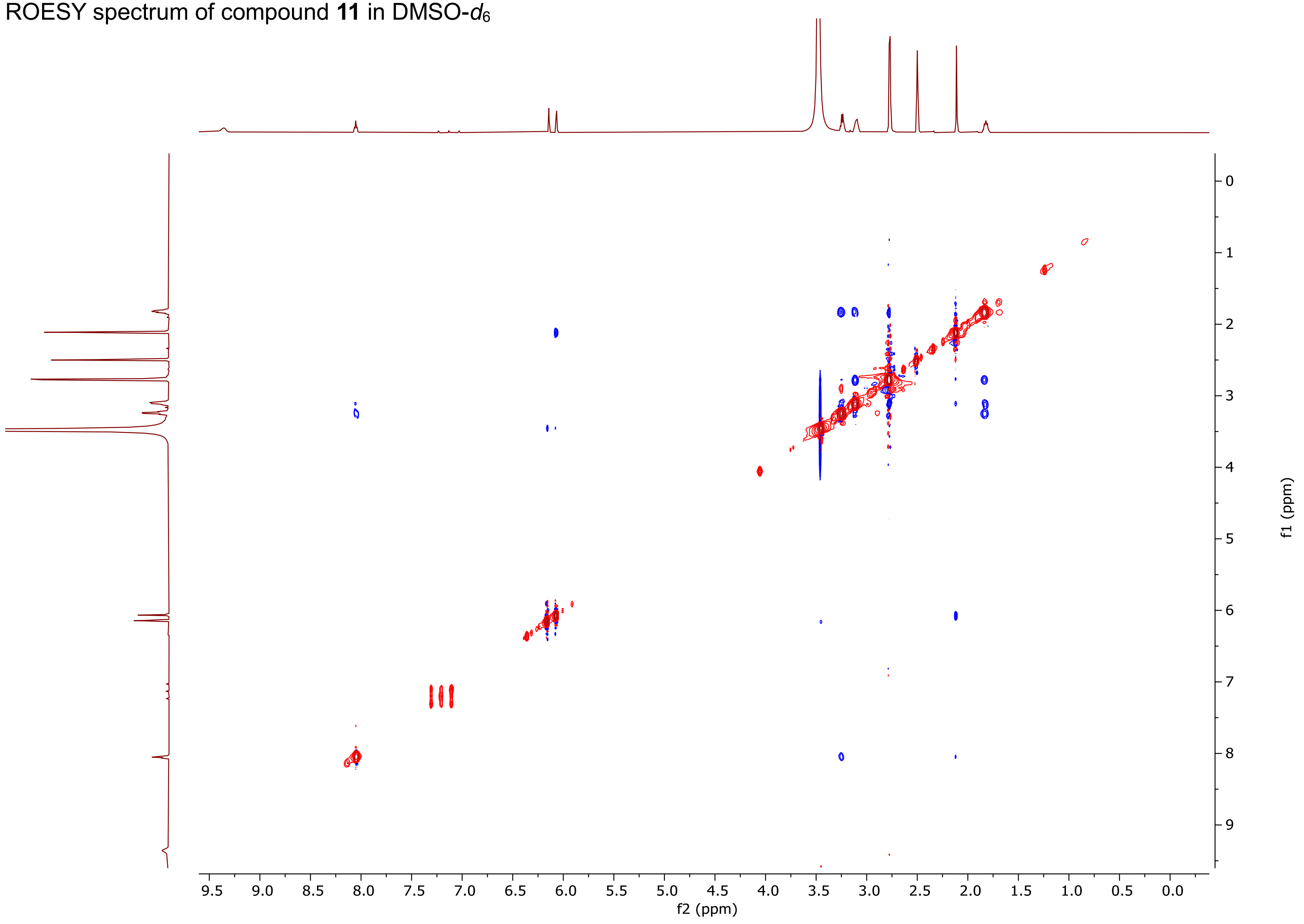

NMR data table for compound **11** in DMSO-d_6[a, b]

Position	δ_H (mult., J in Hz)	δ_C, type	COSY	HMBC	ROESY
1		117.0, C			
2		156.2, C			
2-OH	9.71 (brs)		8,10,11,12		
3	6.16 (d, 2.1)	100.4, CH	5,6-Me^w	1,2,4,5,7^w	
4		158.6, C			
4-OH	9.43 (brs)				
5	6.07 (d, 2.1)	108.5, CH	3,6-Me^w	1,3,4^w,6-Me,7^w	6-Me
6		137.4, C			
6-Me	2.12 (s)	20.0, CH_3	3^w,5^w	1,2^w,3^w,5,6,7^w	5,7-NH
7		168.8, C			
7-NH	8.05 (t, 5.8)		8	7,8^w	6-Me^w,8,9,10^w
8	3.25 (dt, 5.8, 6.2)	36.1, CH_2	7-NH,9	7,9,10	7-NH,9,10
9	1.83 (dt, 6.2, 5.6)	24.8, CH_2	8,10	8^w,10^w	7-NH^w,8,10,11^w,12^w
10	3.11 (tt, 5.6, 5.4)	55.2, CH_2	9,10-NH		6-Me^w,7-NH^w,8,9,11^w,12^w
10-NH	9.59 (brs)[b]		10,11,12		
11	2.78 (s)	42.8, CH_3	10-NH	10,12	9,10
12	2.77 (s)	42.8, CH_3	10-NH	10,11	9,10

[a] Spectra recorded at 25 °C (500 MHz for ^{1}H NMR and 125 MHz for ^{13}C NMR); [b] Purified as a TFA salt; [w] Weak correlation.

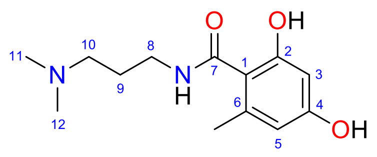

High resolution mass spectrum of compound **11**

Mass Spectrum SmartFormula Report

Analysis Info

Analysis Name	D:\Data\Sasha\20231004\RAD915J000002.d	Acquisition Date	10/4/2023 2:59:16 PM
Method	DirectInfusion_2018_pos.m	Operator	Demo User
Sample Name	RAD915J	Instrument	maXis II ETD
Comment			

Acquisition Parameter

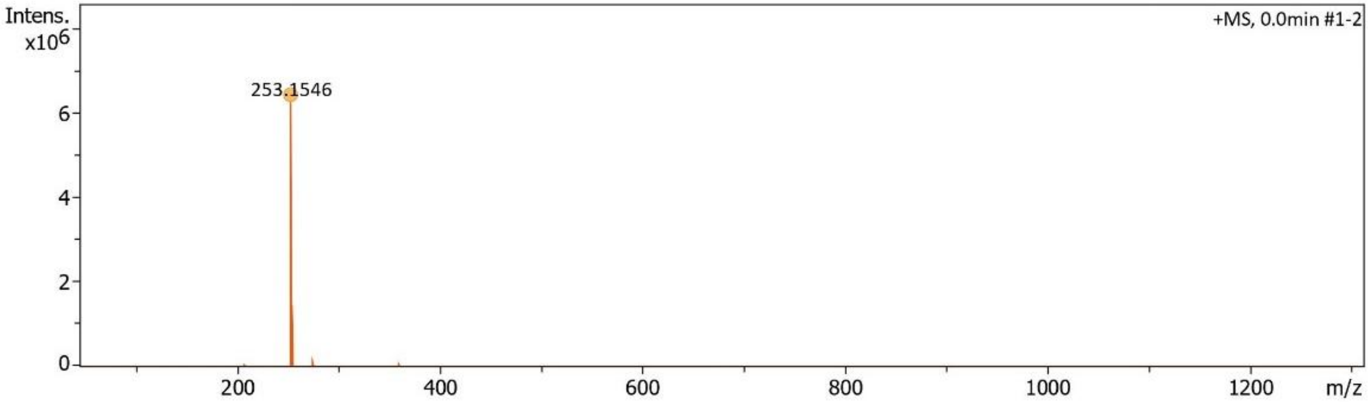

Meas. m/z	#	Ion Formula	m/z	err [ppm]	mSigma	# mSigma	Score	rdb	e⁻ Conf	N-Rule
253.1546	1	C13H21N2O3	253.1547	0.1	48.6	1	100.00	5.0	even	ok

^{1}H NMR spectrum of compound **12** in DMSO-d_6

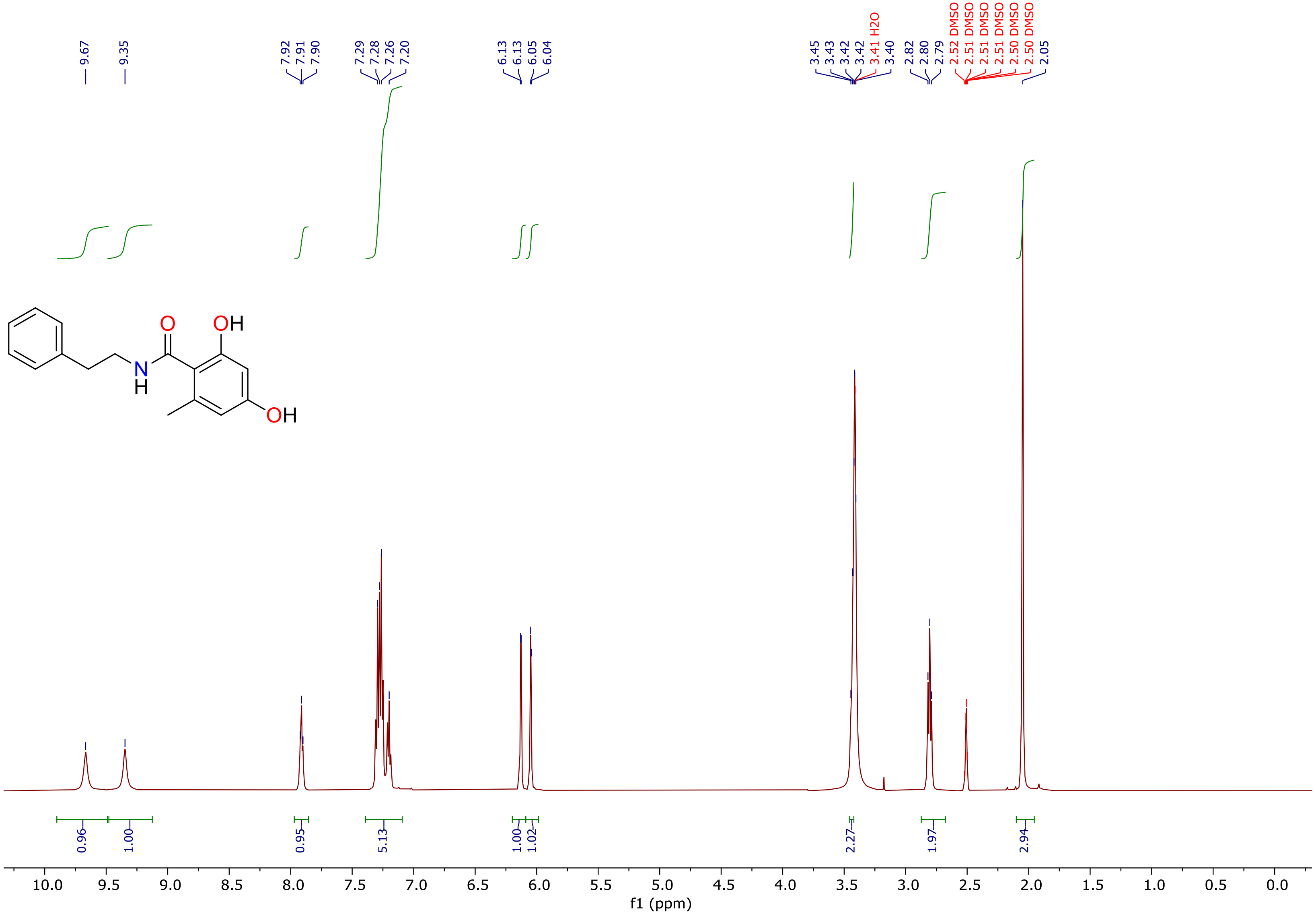

^{1}H NMR spectrum of compound **12** in DMSO-d_6 and D_2O

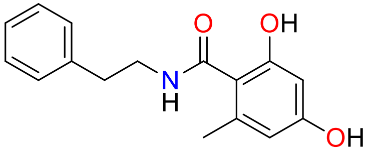

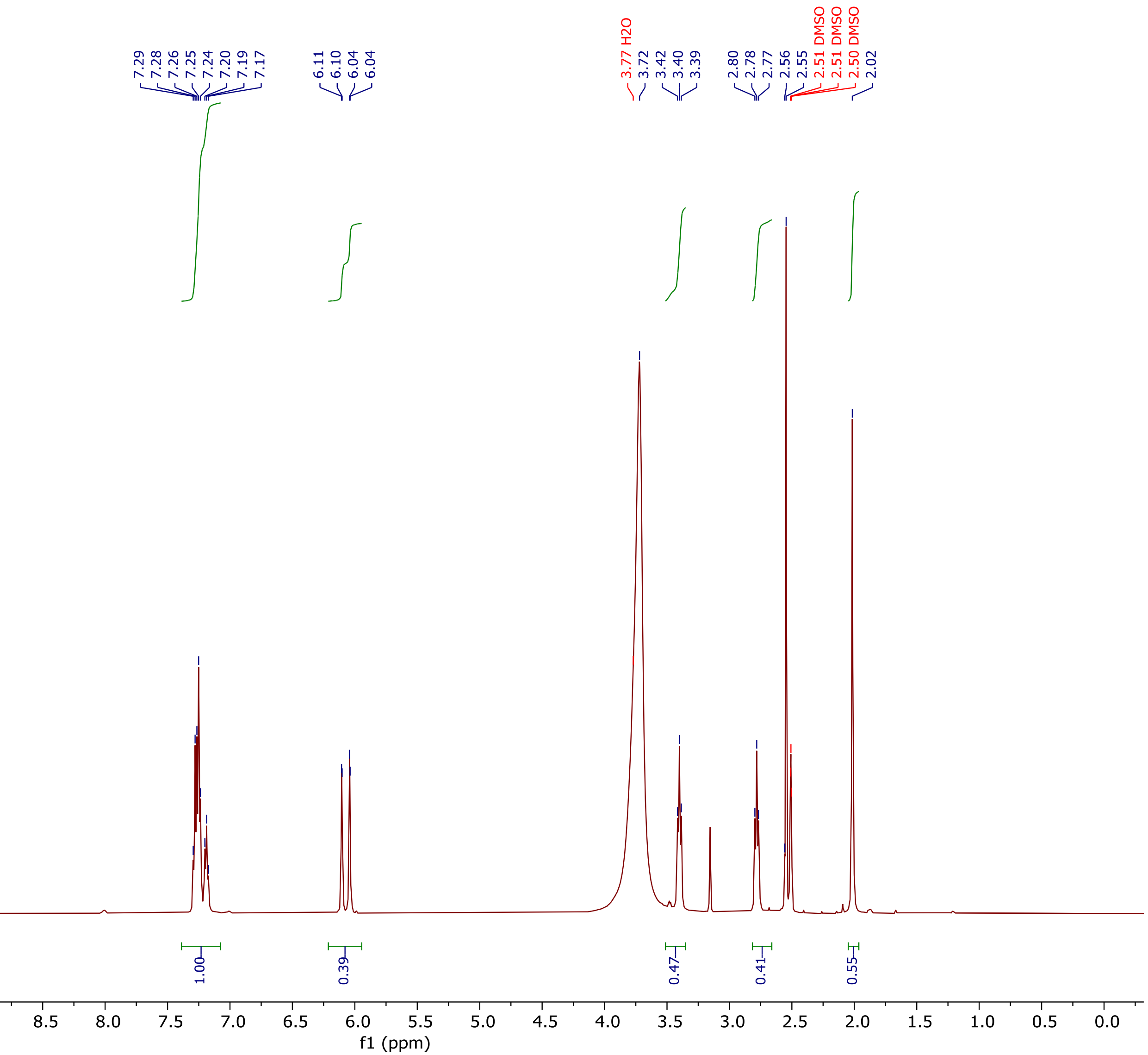

^{13}C NMR spectrum of compound **12** in DMSO-d_6

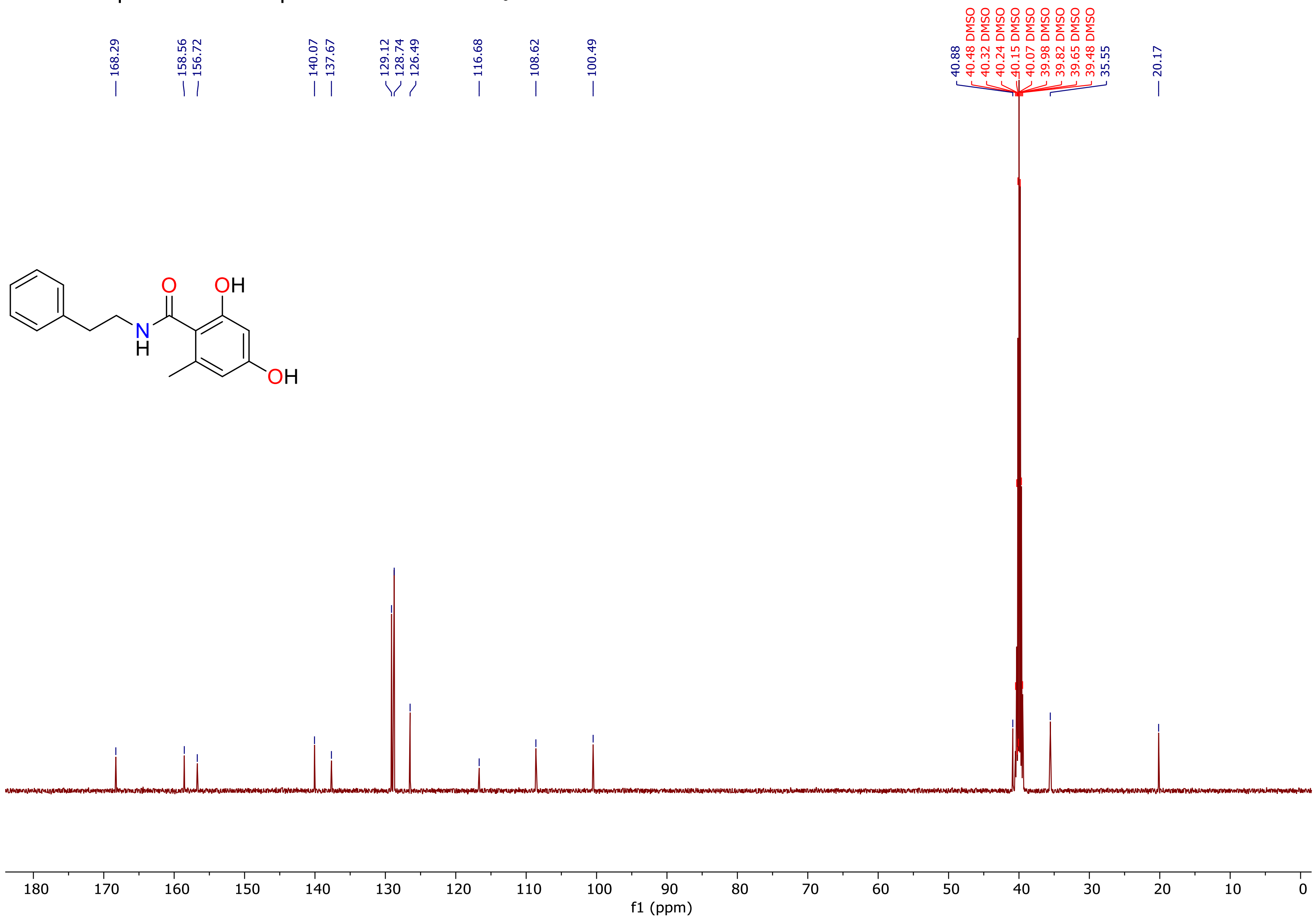

HSQC spectrum of compound **12** in DMSO-d_6

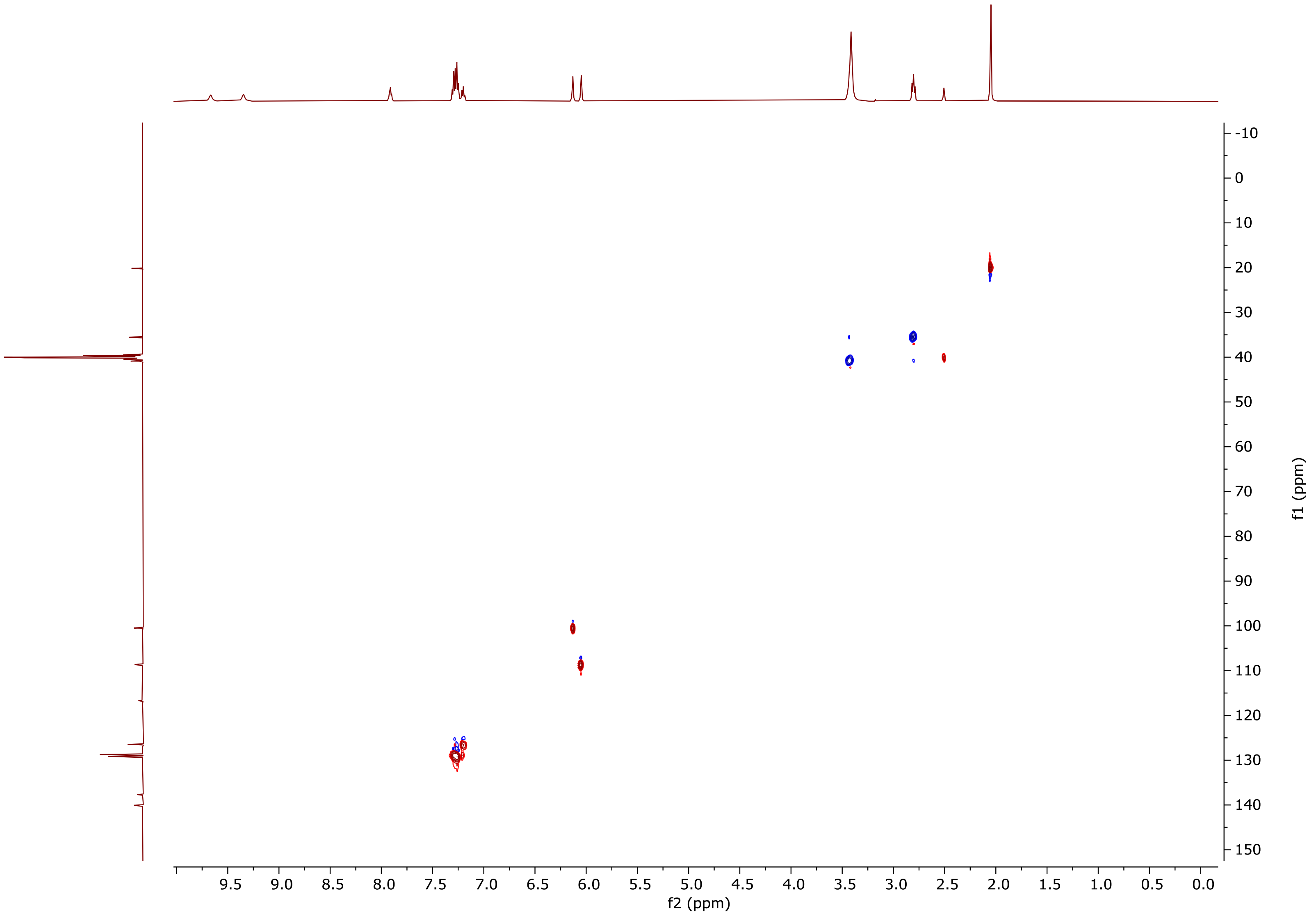

HMBC spectrum of compound **12** in DMSO-d_6

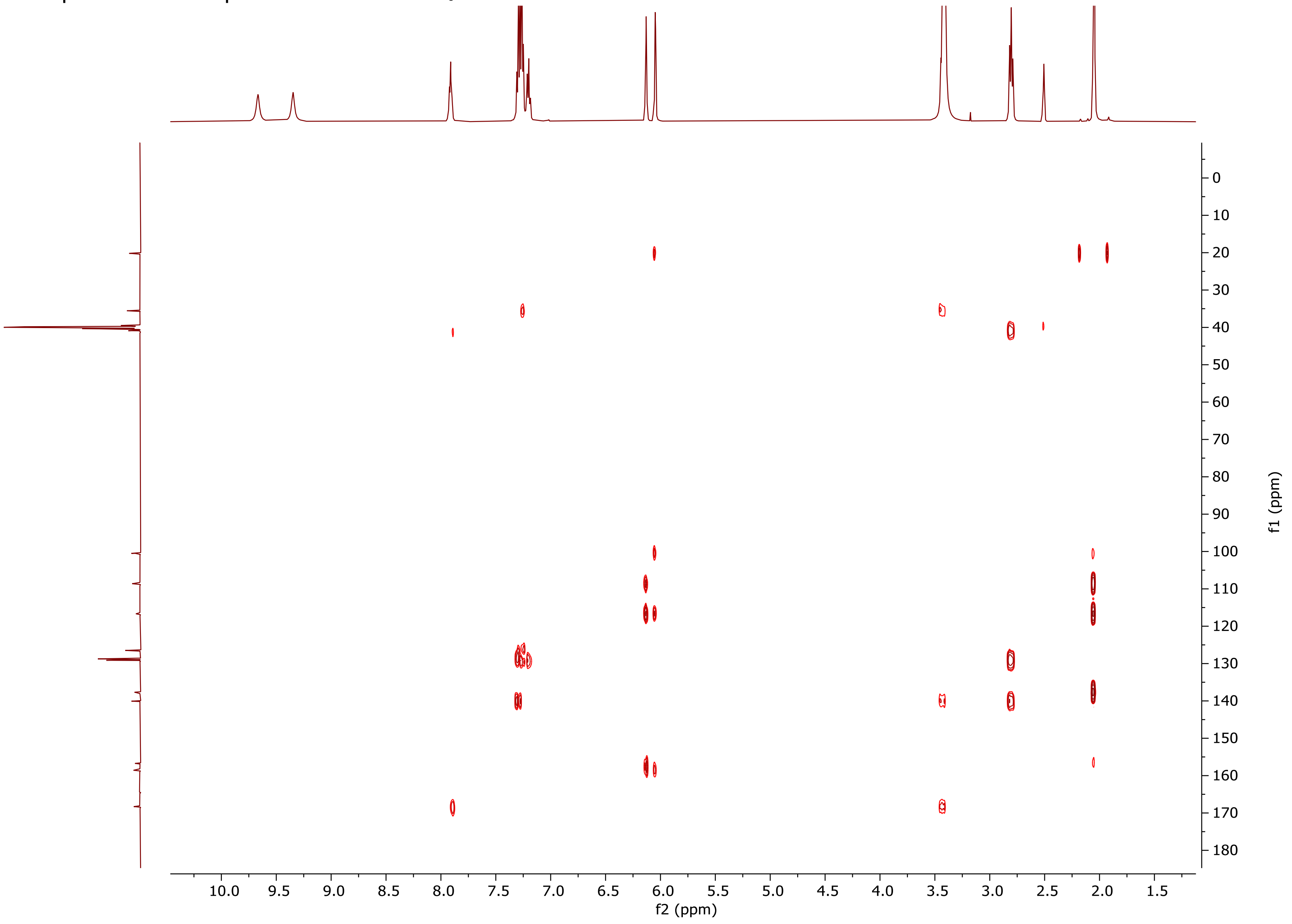

COSY spectrum of compound **12** in DMSO-d_6

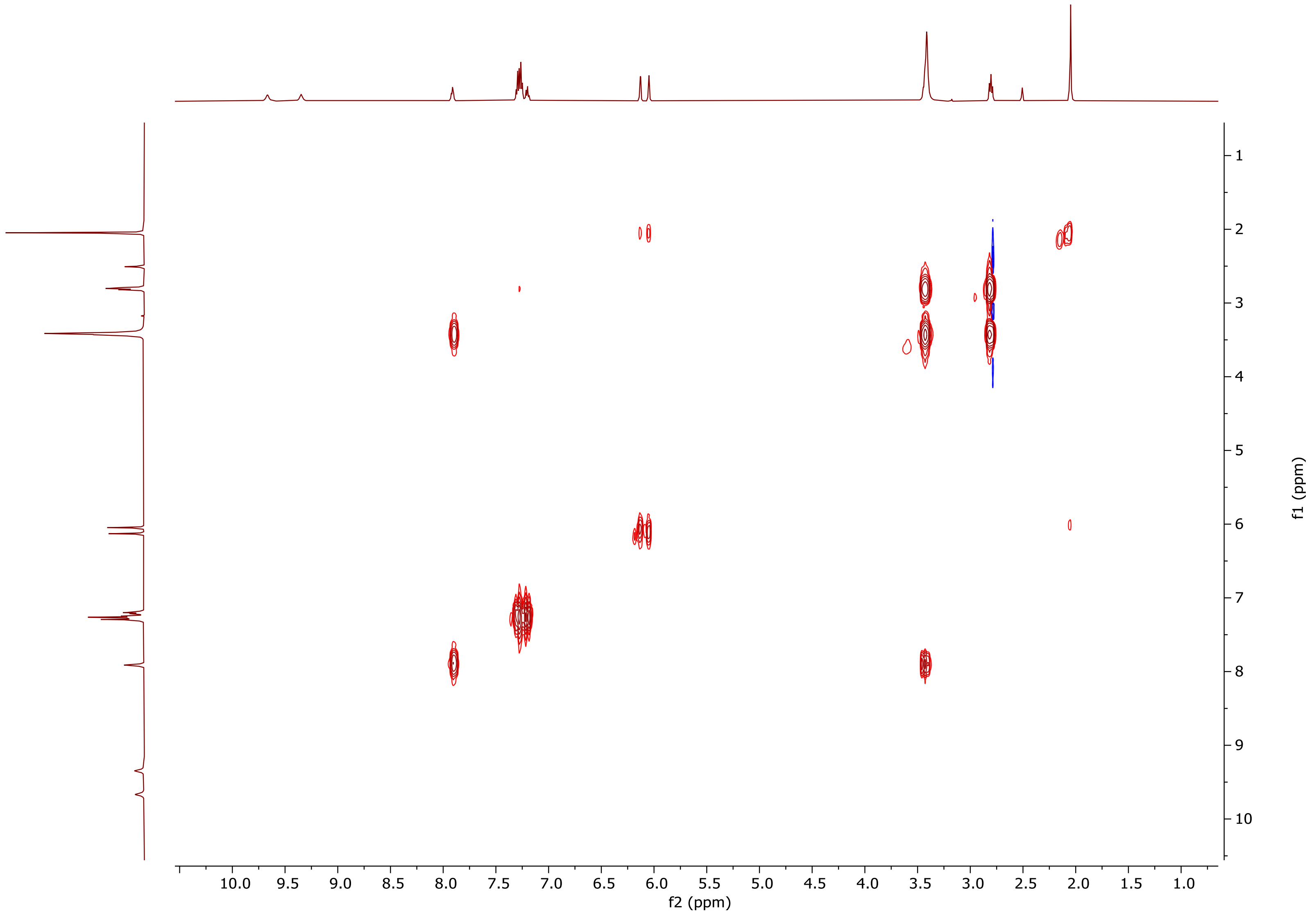

ROESY spectrum of compound **12** in DMSO-d_6

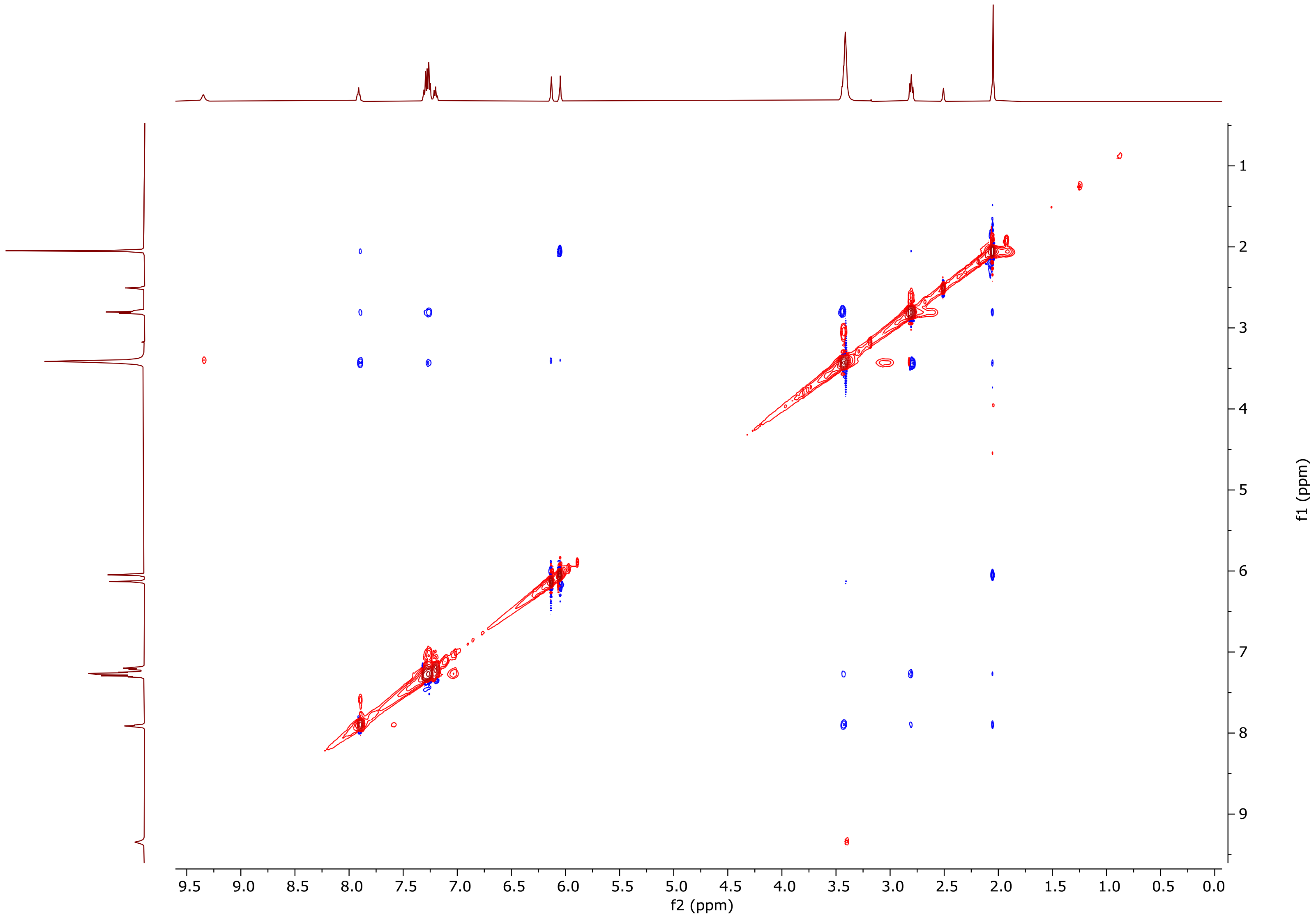

NMR data table for compound **12** in DMSO-d_6[a]

Position	δ_H (mult., J in Hz)	δ_C, type	COSY	HMBC	ROESY
1		116.7, C			
2		156.7, C			
2-OH	9.67 (brs)				
3	6.13 (d, 2.0)	100.5, CH	5,6-Me,8^w	1,2,4,5,7^w	4-OH^w,6-Me^w,7-NH^w,8
4		158.6, C			
4-OH	9.35 (brs)				3,5
5	6.05 (d, 2.0)	108.6, CH	3,6-Me	1,2,3,6-Me,7^w	4-OH^w,6-Me
6		137.7, C			
6-Me	2.05 (s)	20.2, CH_3	3,5	1,2,3^w,5,6,7^w,10	5,7-NH,8,9
7		168.3, C			
7-NH	7.91 (t, 5.5)		8	7,8	6-Me,8,9,15^w
8	3.42 (dt, 5.5, 7.4)	40.9, CH_2	7-NH,9	7,9,10	3^w,6-Me^w,7-NH,9,11,15
9	2.80 (t, 7.4)	35.6, CH_2	8,11,13,15	8,10,11,15	6-Me,7-NH^w,8,11,15
10		140.1, C			
11	7.26 (br d, 7.3)	129.1, CH	9^w,12	9,12,13,14,15	8,9
12	7.29 (br dd, 7.1, 7.3)	126.5, CH	11,13	10,11,14,15	
13	7.20 (tt, 7.1, 1.6)	128.7, CH	11,12,14,15	11,15	
14	7.29 (br dd, 7.1, 7.3)	126.5, CH	13,15	10,11,12,15	
15	7.26 (br d, 7.3)	129.1, CH	9^w,14	9,11,12,13,14	8,9

[a] Spectra recorded at 25 °C (500 MHz for ^{1}H NMR and 125 MHz for ^{13}C NMR); [w] Weak correlation.

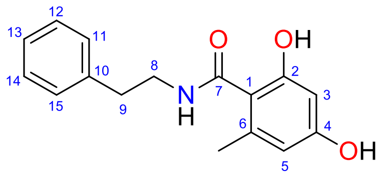

High resolution mass spectrum of compound **12**

Mass Spectrum SmartFormula Report

Analysis Info

Analysis Name	D:\Data\Sasha\20231004\RAD915M000001.d	Acquisition Date	10/4/2023 2:47:09 PM
Method	DirectInfusion_2018_pos.m	Operator	Demo User
Sample Name	RAD915M	Instrument	maXis II ETD
Comment			

Acquisition Parameter

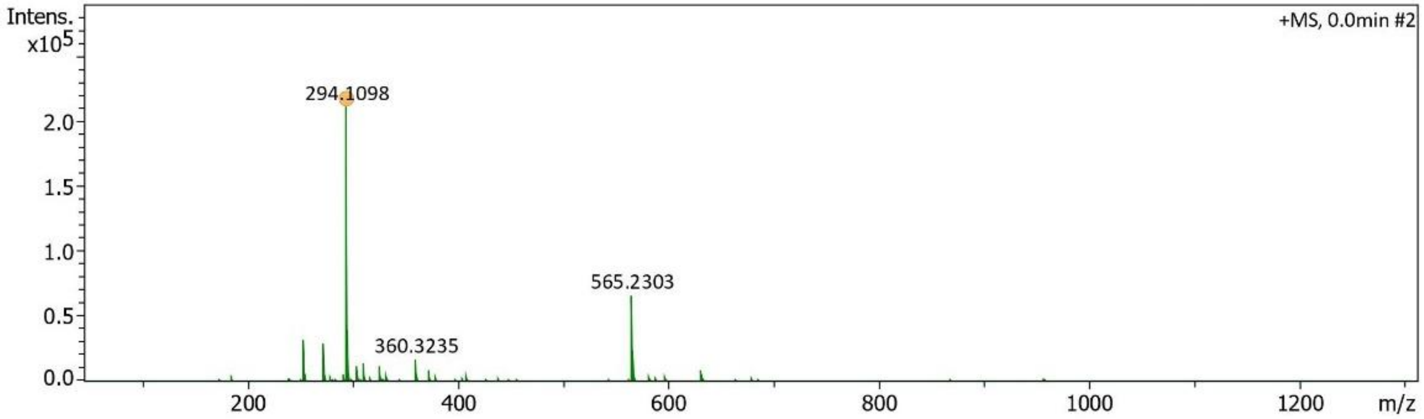

Meas. m/z	#	Ion Formula	m/z	err [ppm]	mSigma	# mSigma	Score	rdb	e⁻ Conf	N-Rule
294.1098	1	C16H17NNaO3	294.1101	0.8	3.0	1	100.00	9.0	even	ok

^{1}H NMR spectrum of compound **13** in DMSO-d_6

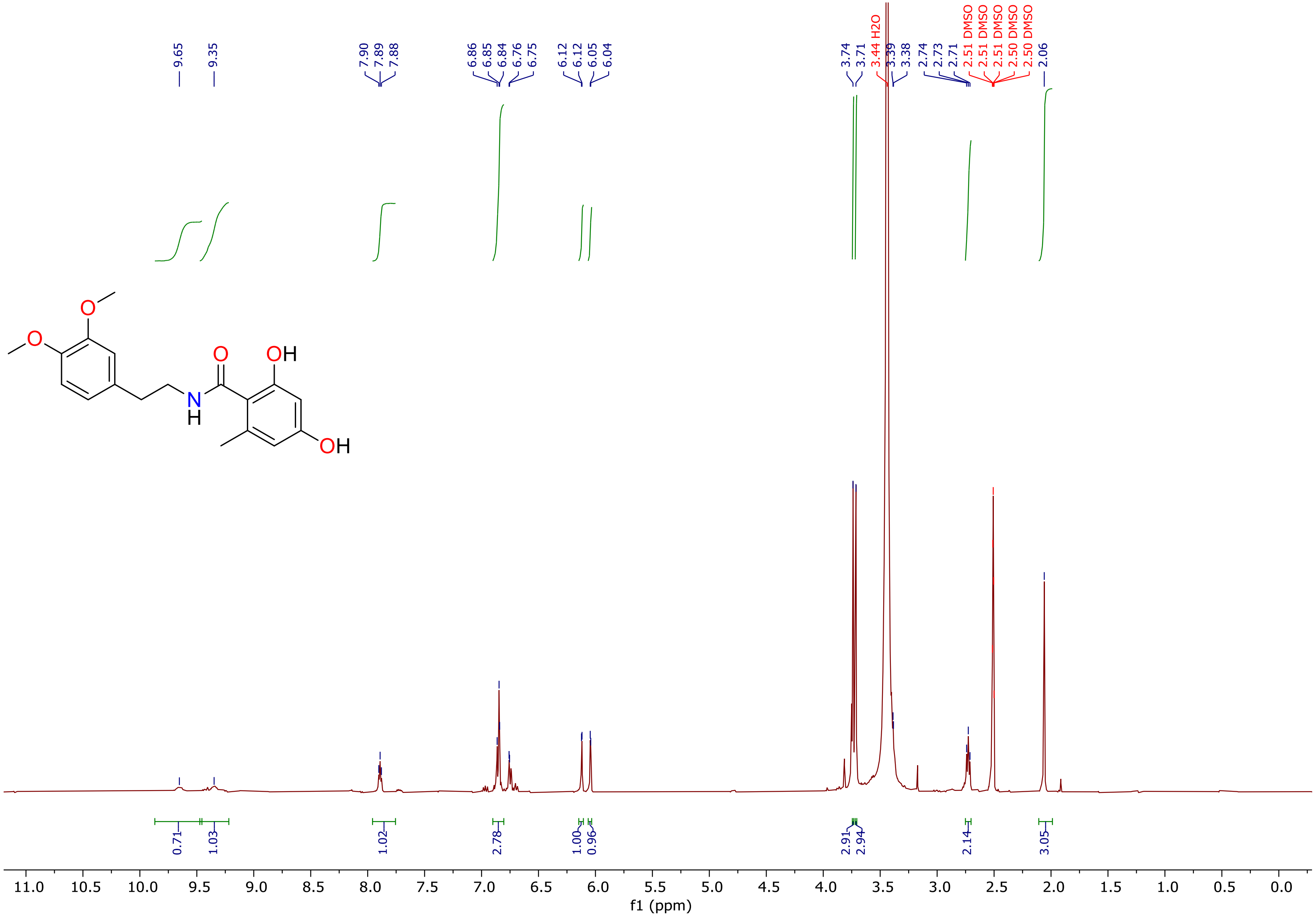

^{13}C NMR spectrum of compound **13** in DMSO-d_6

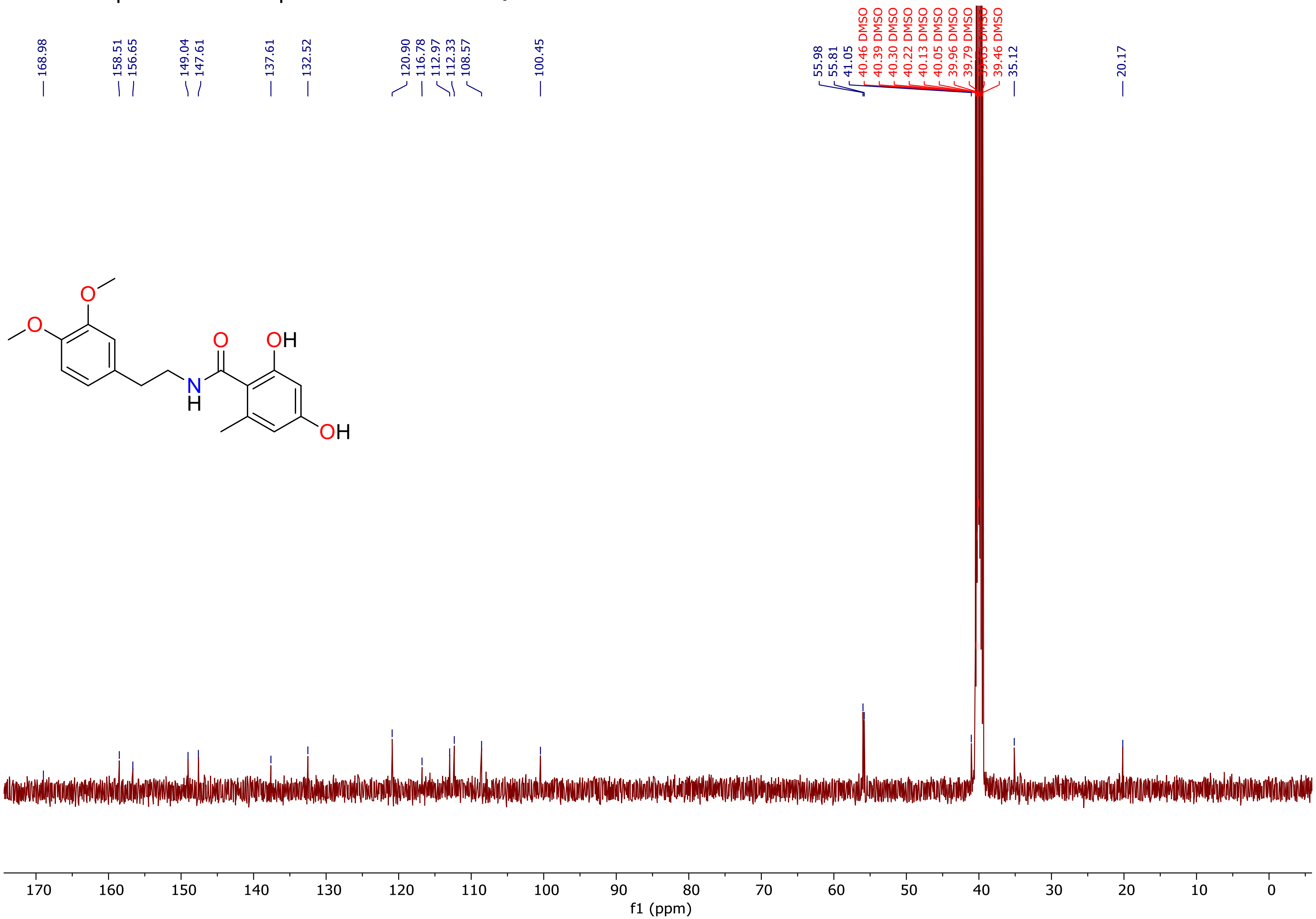

HSQC spectrum of compound **13** in DMSO-d_6

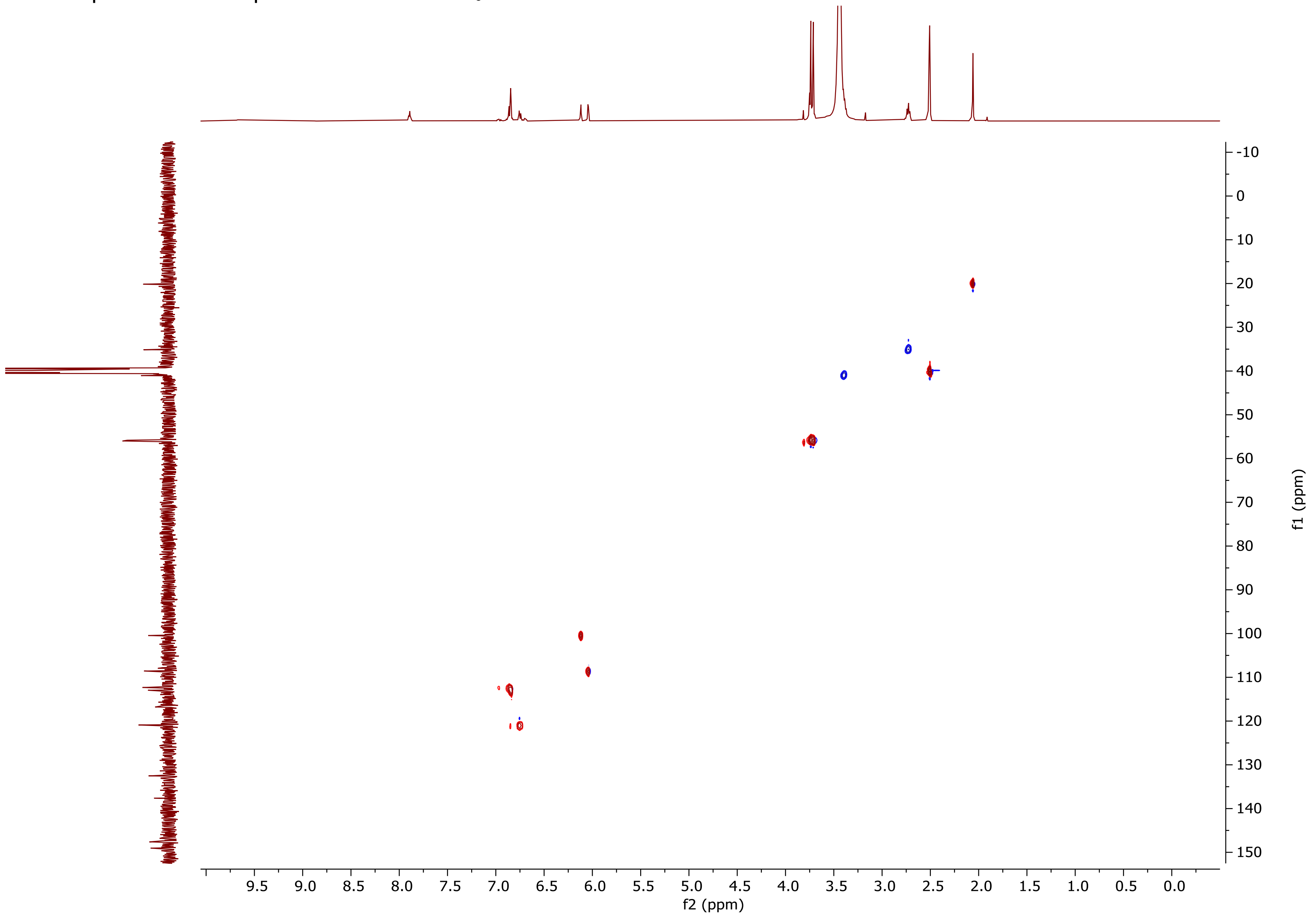

HMBC spectrum of compound **13** in DMSO-d_6

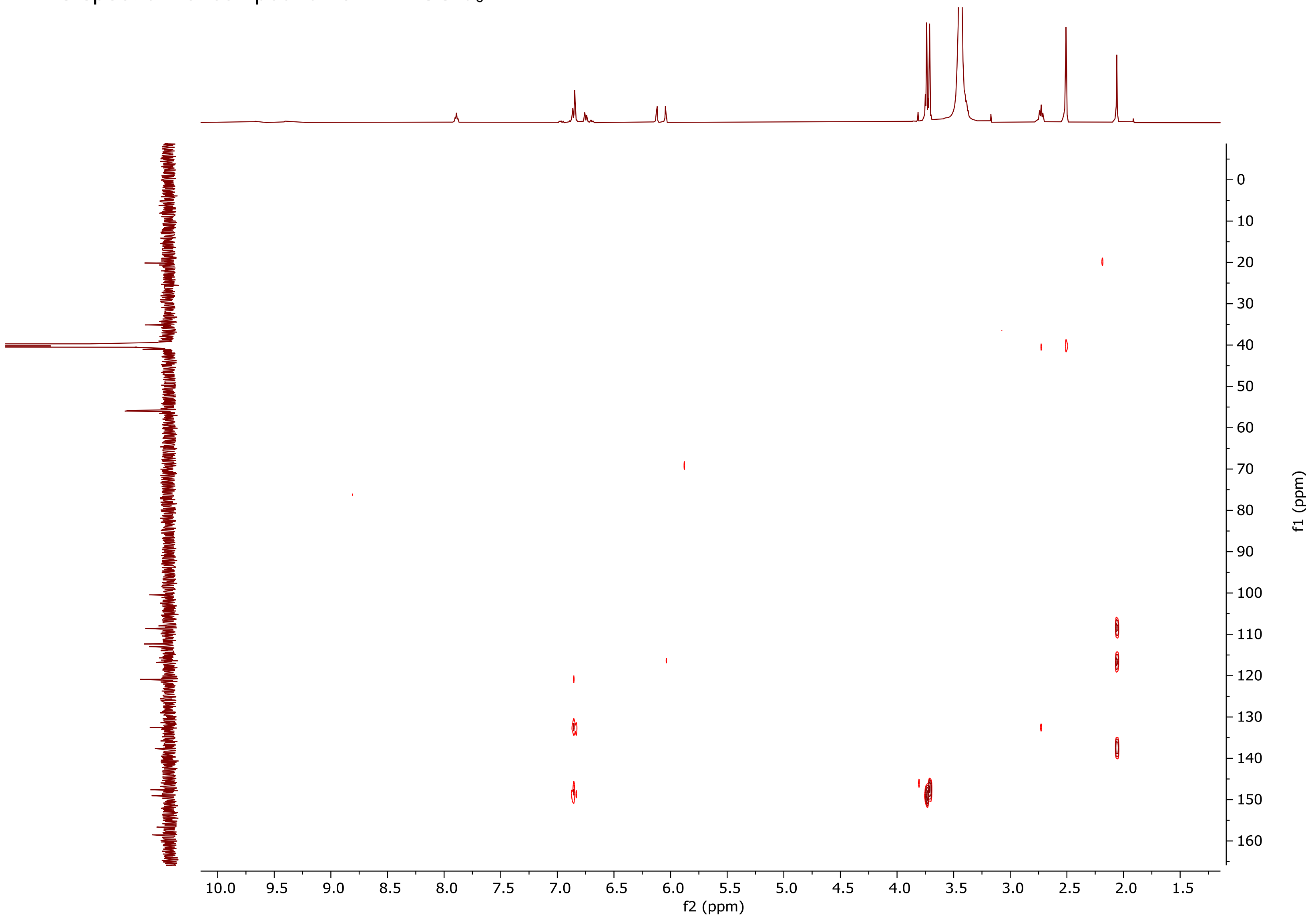

COSY spectrum of compound **13** in DMSO-d_6

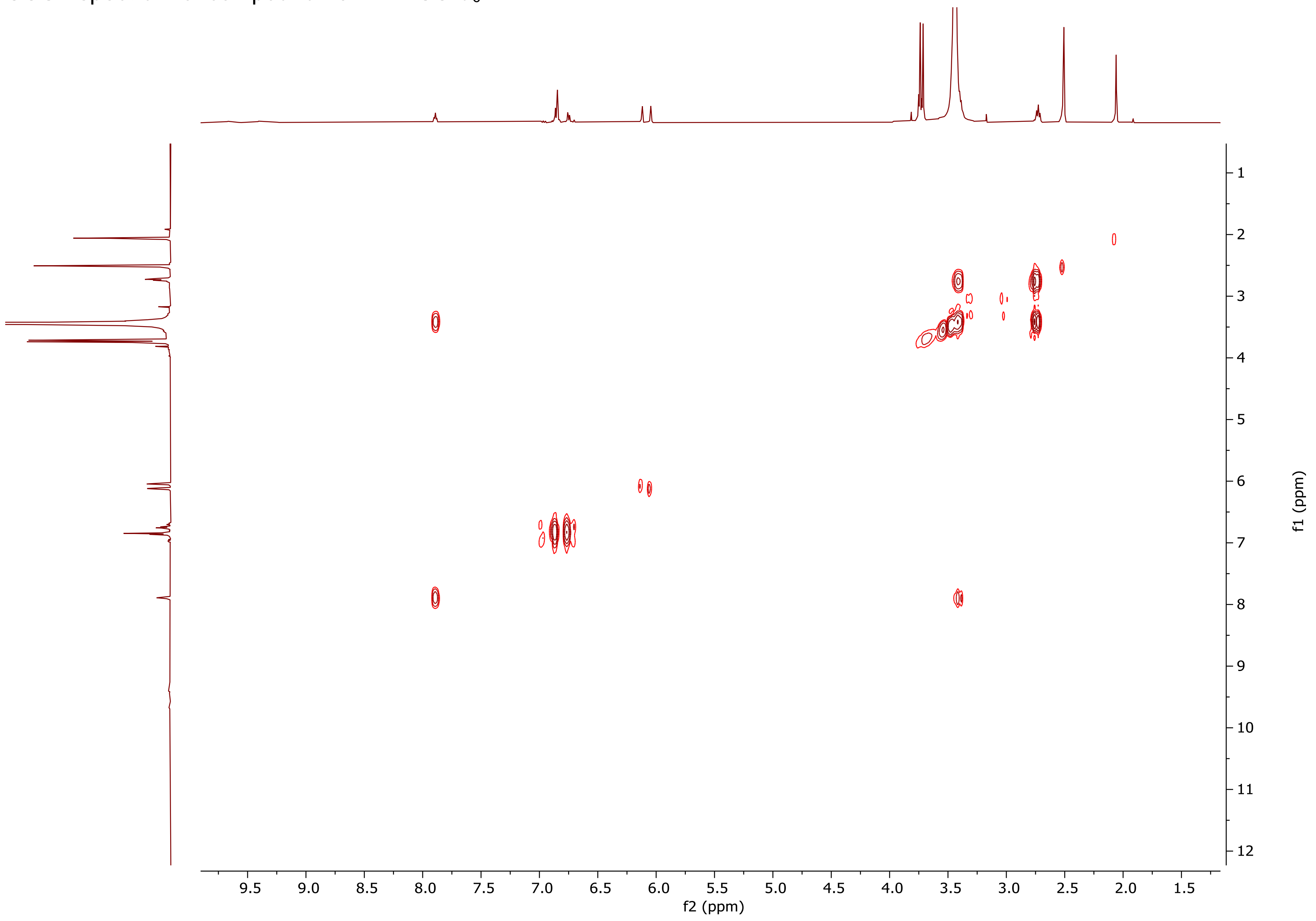

ROESY spectrum of compound **13** in DMSO-d_6

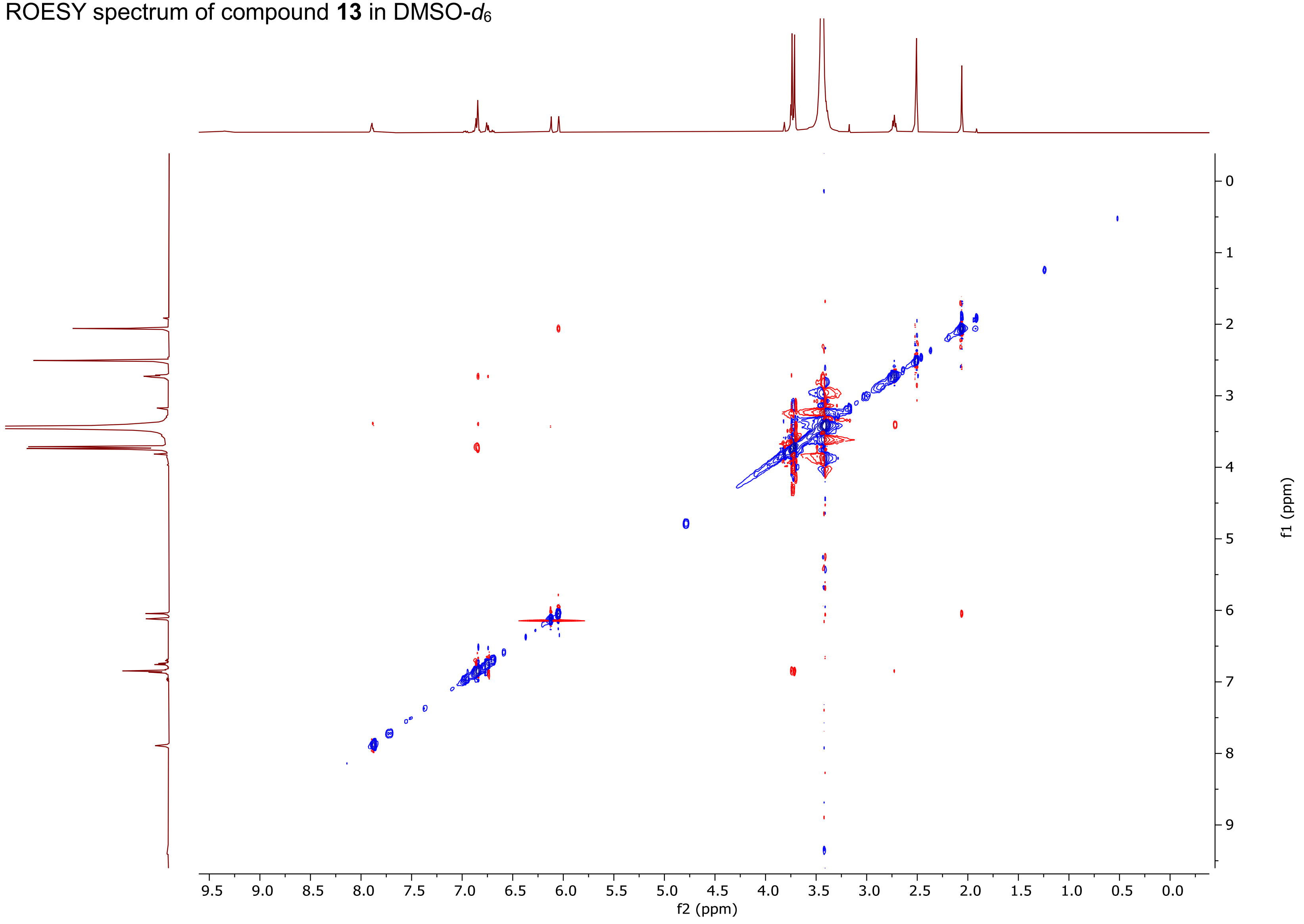

NMR data table for compound **13** in DMSO-d_6[a]

Position	δ_H (mult., *J* in Hz)	δ_C, type	COSY	HMBC	ROESY
1		116.8, C			
2		156.7, C			
2-OH	9.65 (brs)				
3	6.12 (d, 2.0)	100.5, CH	5,6-Me[w]	1	
4		158.5, C			
4-OH	9.35 (brs)				
5	6.04 (d, 2.0)	108.6, CH	3,6-Me[w]	1,6-Me[w]	6-Me
6		137.6, C			
6-Me	2.06 (s)	20.2, CH_3	5	1,5,6	5
7		169.0, C			
7-NH	7.89 (t, 5.5)	-	8		8
8	3.39 (dt, 5.5, 7.4)	41.1, CH_2	7-NH,9	9	7-NH,8,9,11,15
9	2.73 (t, 7.4)	35.1, CH_2	8	8,10,11[w],15	8,11,15
10		132.5, C			
11	6.84 (d, 2.0)	113.0, CH	9[w]	10,12,15	8,9,12-OMe
12		149.0, C			
12-OMe	3.71 (brs)	56.0, CH_3	11	12	11
13		147.6, C			
13-OMe	3.74 (brs)	55.8, CH_3	12-OMe	13	14
14	6.86 (d, 8.0)	112.3, CH	15	10,12[w]	13-OMe
15	6.75 (dd, 2.0, 8.0)	120.9, CH		14[w]	9

[a] Spectra recorded at 25 °C (500 MHz for ^{1}H NMR and 125 MHz for ^{13}C NMR); [w] Weak correlation.

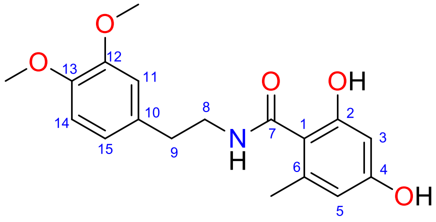

High resolution mass spectrum of compound **13**

Mass Spectrum SmartFormula Report

Analysis Info

Analysis Name	D:\Data\Sasha\20231004\RAD915I000001.d	Acquisition Date	10/4/2023 2:28:42 PM
Method	DirectInfusion_2018_pos.m	Operator	Demo User
Sample Name	RAD915I	Instrument	maXis II ETD
Comment			

Acquisition Parameter

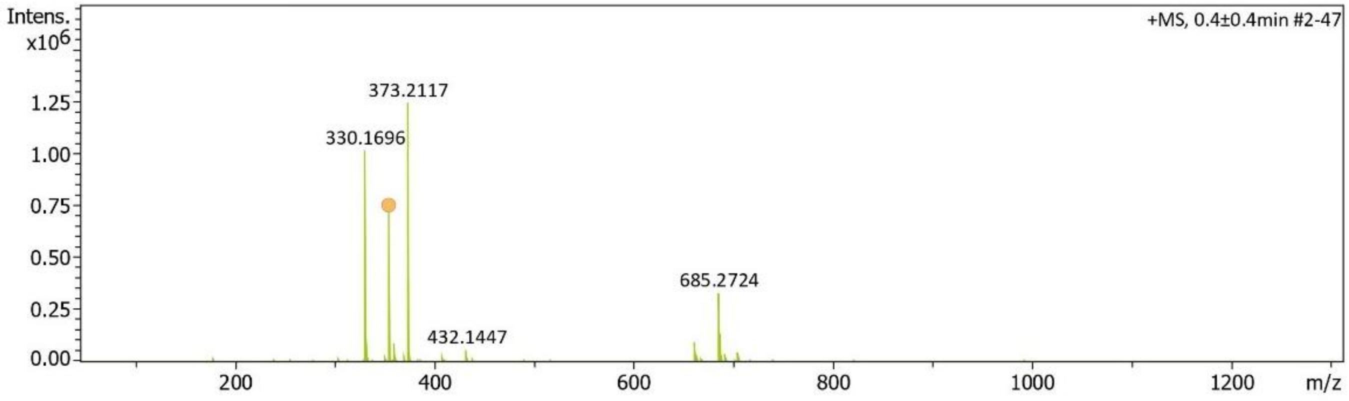

Meas. m/z	#	Ion Formula	m/z	err [ppm]	mSigma	# mSigma	Score	rdb	e¯ Conf	N-Rule
354.1308	1	C18H21NNaO5	354.1312	1.2	0.7	1	100.00	9.0	even	ok
	2	C19H17N5NaO	354.1325	5.0	14.1	2	35.81	14.0	even	ok
